# Reactivity of bromoselenophenes in palladium-catalyzed direct arylations

**DOI:** 10.3762/bjoc.13.278

**Published:** 2017-12-22

**Authors:** Aymen Skhiri, Ridha Ben Salem, Jean-François Soulé, Henri Doucet

**Affiliations:** 1 Institut des Sciences Chimiques de Rennes, UMR 6226 CNRS-Université de Rennes 1, "Organométalliques: Matériaux et Catalyse", Campus de Beaulieu, 35042 Rennes, France; 2 Laboratoire de Laboratoire de Chimie Organique LR 17ES08, Université de Sfax, Faculté des Sciences de Sfax, Route de la Soukra km 4, 3038 Sfax, Tunisia

**Keywords:** C–H bond activation, catalysis, heteroarenes, palladium, selenophene

## Abstract

The reactivity of 2-bromo- and 2,5-dibromoselenophenes in Pd-catalyzed direct heteroarylation was investigated. From 2-bromoselenophene, only the most reactive heteroarenes could be employed to prepare 2-heteroarylated selenophenes; whereas, 2,5-dibromoselenophene generally gave 2,5-di(heteroarylated) selenophenes in high yields using both thiazole and thiophene derivatives. Moreover, sequential catalytic C2 heteroarylation, bromination, catalytic C5 arylation reactions allowed the synthesis of unsymmetrical 2,5-di(hetero)arylated selenophene derivatives in three steps from selenophene.

## Introduction

**Scheme 1:**
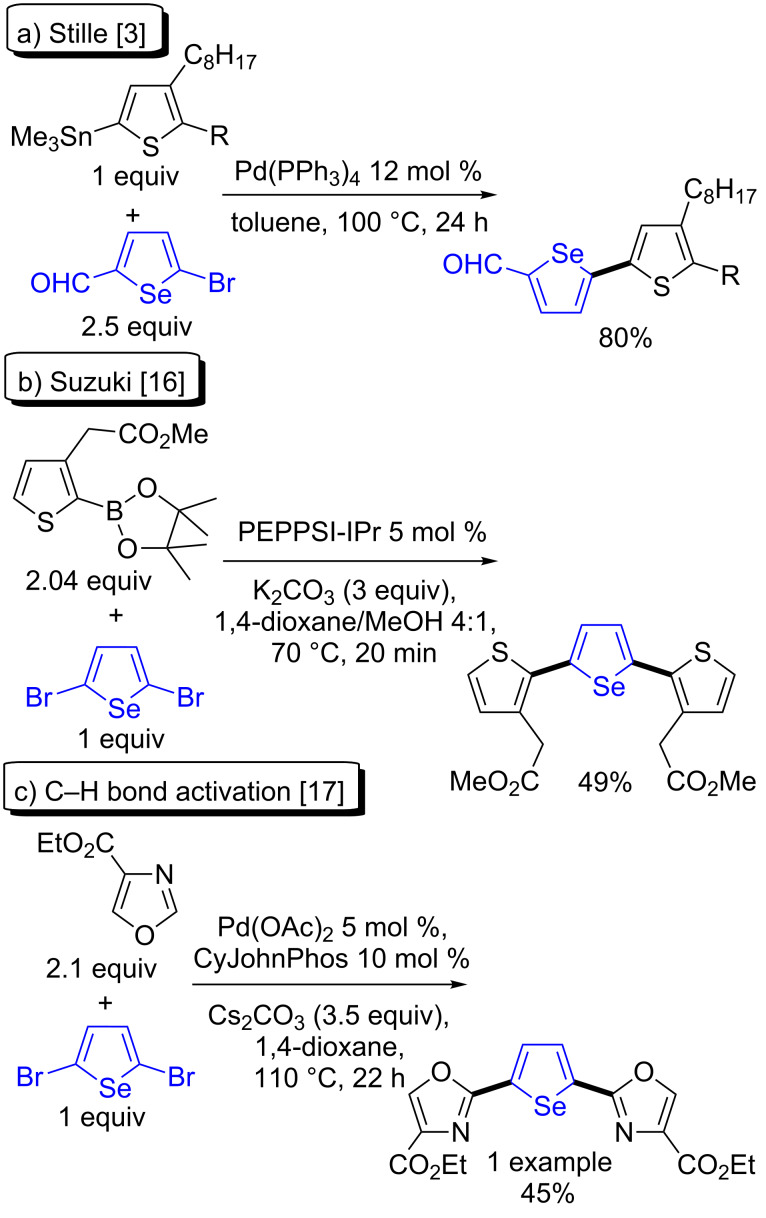
Reported Pd-catalyzed heteroarylations of bromoselenophenes.

(Hetero)aryl-substituted selenophenes represent a class of molecules which exhibit useful physical properties, especially for the preparation of artificial photosynthetic systems for solar energy conversion or for thin film transistor applications [1–3]. In most cases, these (hetero)arylated selenophenes are currently prepared through the use of transition-metal-mediated reactions such as Stille [3–9], Suzuki [10–19], or Kumada [20] couplings [21] (Scheme 1a and b). However, all these procedures require the preparation of an organometallic or a boron derivative of one of the coupling partners, and provide an organometallic salt (MX) as waste. In recent years, the Pd-catalyzed arylation, via a C–H bond activation, of a broad range of heteroaromatics using aryl halides as reaction partners was demonstrated to be particularly effective for the preparation of bi(hetero)aryls [22–31]. Among the reported results, a few examples of Pd-catalyzed direct arylations via the C–H bond activation of selenophenes using aryl halides as coupling partners have been reported [32–35]. Conversely, C–H bond activation methodology was employed in only in one case for the preparation of a heteroarylated selenophene from a haloselenophene. Wipf et al. reported in 2014 that using ethyl oxazole-4-carboxylate as reaction partner, the corresponding 2,5-bis(oxazol-2-yl)selenophene derivative was formed in 45% yield (Scheme 1c) [17]. Moreover, to our knowledge the Pd-catalyzed direct heteroarylation of 2-bromoselenophene has not yet been described. Therefore, the reactivity of 2-bromo- and 2,5-dibromoselenophenes in Pd-catalyzed direct couplings with heteroarenes needed to be investigated.

Herein, we wish to report on the reactivity of 2-bromoselenophene, 2,5-dibromoselenophene and 2-aryl-5-bromoselenophenes in palladium-catalyzed direct heteroarylations with a variety of heteroarenes using a phosphine-free palladium catalyst.

## Results and Discussion

First, we examined the influence of the reaction temperature, using DMA as solvent, KOAc as base and 2 mol % Pd(OAc)_2_ as catalyst (Table 1). We had previously observed that these reaction conditions allowed the coupling of several heteroaromatics such as thiazole, pyrrole, furan or imidazole derivatives with aryl bromides [36]. 2-Bromoselenophene, which was easily prepared by reaction of selenophene with *N*-bromosuccinimide [37], and 2-ethyl-4-methylthiazole were employed as model substrates for our study. Reactions performed at 130 °C or 110 °C gave the expected arylated selenophene **1** in 55% and 64% yields, respectively, with complete conversions of 2-bromoselenophene; whereas **1** was obtained in a higher yield of 80% when the reaction was conducted at 90 °C (Table 1, entries 1–3). At elevated temperature (110–130 °C), 2-bromoselenophene seems to afford larger amounts of selenophene oligomers as side-products. The use of PdCl_2_ or PdCl(C_3_H_5_)(dppb) as catalysts instead of Pd(OAc)_2_ or other bases such as K_2_CO_3_, Cs_2_CO_3_, CsOAc or NaOAc afforded **1** in lower yields (Table 1, entries 4–9). The influence of two other solvents in this cross-coupling reaction was also examined. We observed that both DMF and xylene in the presence of 2 mol % Pd(OAc)_2_ catalyst with KOAc gave **1** in moderate yields (Table 1, entries 10 and 11).

**Table 1:** Influence of the reaction conditions for the coupling of 2-ethyl-4-methylthiazole with 2-bromoselenophene.^a^

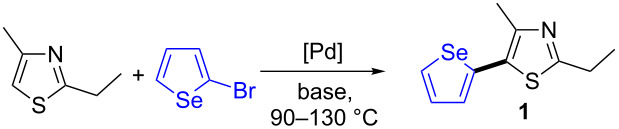				

Entry	Catalyst	Solvent	Base	Yield (%)

1	Pd(OAc)_2_	DMA	KOAc	55^b^
2	Pd(OAc)_2_	DMA	KOAc	64^c^
3	Pd(OAc)_2_	DMA	KOAc	80
4	PdCl_2_	DMA	KOAc	45
5	PdCl(C_3_H_5_)(dppb)	DMA	KOAc	48
6	Pd(OAc)_2_	DMA	K_2_CO_3_	41
7	Pd(OAc)_2_	DMA	Cs_2_CO_3_	9
8	Pd(OAc)_2_	DMA	CsOAc	52
9	Pd(OAc)_2_	DMA	NaOAc	17
10	Pd(OAc)_2_	DMF	KOAc	53
11	Pd(OAc)_2_	xylene	KOAc	48

^a^Conditions: catalyst (0.02 equiv), 2-bromoselenophene (1 equiv), 2-ethyl-4-methylthiazole (1.5 equiv), base (2 equiv), 24 h, 90 °C, isolated yields of **1**. ^b^130 °C, ^c^110 °C.

**Scheme 2:**
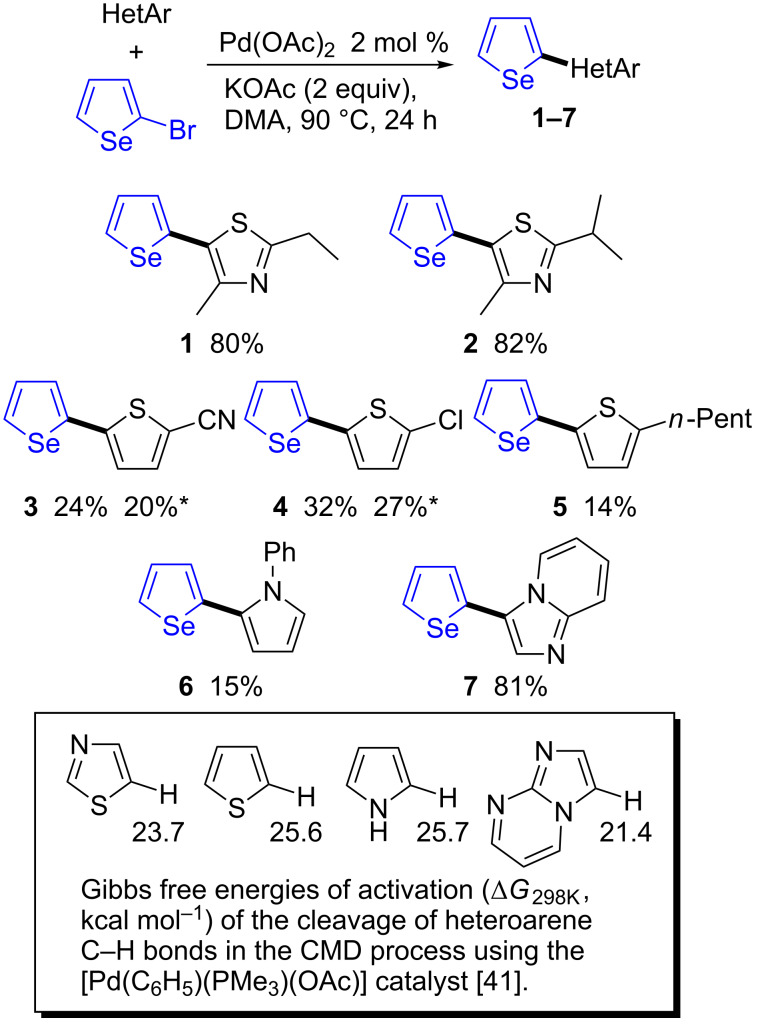
Palladium-catalyzed heteroarylations of 2-bromoselenophene. *: 110 °C

Then, we investigated the scope of the coupling of 2-bromoselenophene with a set of heteroarenes in the presence of 2 mol % Pd(OAc)_2_, KOAc as the base in DMA at 90 °C (Scheme 2). The reaction of 2-isopropyl-4-methylthiazole gave the desired product **2** in 82% yield. Conversely, low yields in the target products **3**–**5** were obtained for the reactions with thiophene-2-carbonitrile, 2-chlorothiophene and 2-pentylthiophene, although complete conversions of 2-bromoselenophene were observed. A similar result was obtained for the reaction with 1-phenylpyrrole. Reactions performed at a higher temperature with thiophene-2-carbonitrile, 2-chlorothiophene afforded **3** and **4** in slightly lower yields. Gorelsky calculated that the Gibbs free energies of activation for the cleavage of C–H bonds at C5-position of thiophene or pyrrole derivatives, for reaction which proceed via concerted metallation–deprotonation [38–40], are higher than that of thiazoles (see bottom of Scheme 2) [42]. We assume that, due to these higher energies of activation for reactions with thiophene or pyrrole, larger amounts of selenophene oligomers were formed in the presence of these less reactive heteroarenes. Then, the reactivity of imidazo[1,2-*a*]pyridine, which contains a very reactive C–H bond at C3-position was examined. The expected product **7** was obtained in a high yield of 81%. Thus, for Pd-catalyzed direct heteroarylations of 2-bromoselenophene, only the heteroarenes containing C–H bonds with low Gibbs free energies of activation [41] should be employed.

**Scheme 3:**
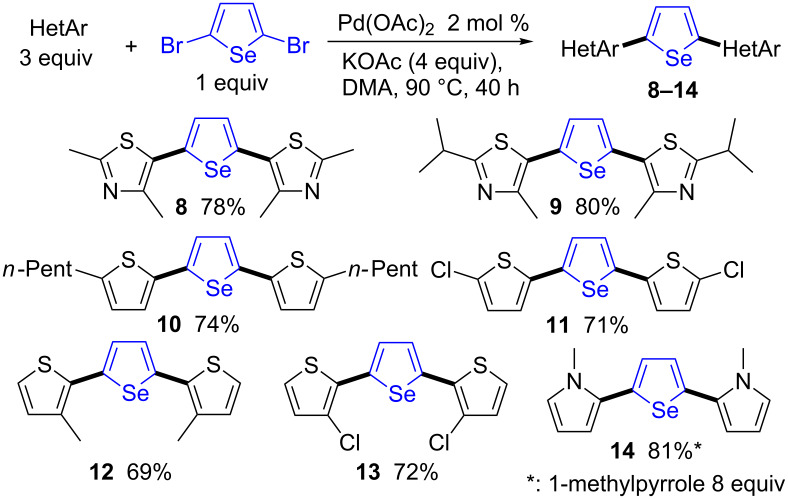
Palladium-catalyzed 2,5-diheteroarylation of 2,5-dibromoselenophene.

By contrast, the direct arylation reactions with 2,5-dibromoselenophene were found to tolerate both thiazole and thiophene derivatives (Scheme 3). The coupling of 3 equiv of thiazole derivatives with 2,5-dibromoselenophene in the presence of 2 mol % Pd(OAc)_2_ and KOAc as base gave the corresponding 2,5-diheteroarylated selenophenes **8** and **9** in 78% and 80% yields, respectively. The use of 2-pentyl- and 2-chlorothiophenes also gave the desired products **10** and **11** in high yields. In general, the Pd-catalyzed direct arylation of 3-substituted thiophenes with aryl halides afforded quite regioselectively the C2-arylated thiophenes [30]. A similar regioselectivity was oberved for the coupling of thiophene derivatives containing methyl- or chloro-substituents at the C3-position with 2,5-dibromoselenophene. In both cases, regioselective arylations at the C2-positions were observed, affording the 2,5-diarylated selenophenes **12** and **13** in 69% and 72% yields, respectively. From 2,5-dibromoselenophene and an excess of 1-methylpyrrole, the expected 2,5-diarylated selenophene **14** was obtained in 81% yield.

**Scheme 4:**
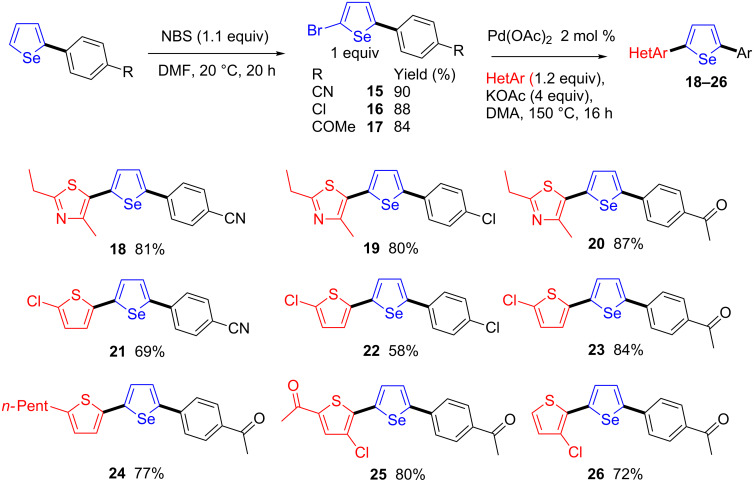
Synthesis of 2-aryl-5-(heteroaryl)selenophenes.

Finally, we show a sequential transformation leading to 2-aryl-5-(heteroaryl)selenophenes in three steps from commercially available compounds (Scheme 4). Bromination at the C5-position of 2-arylselenophenes containing nitrile, acetyl or chloro substituents on the aryl moiety, which could be easily obtained in good yields from selenophene and aryl bromides via a Pd-catalyzed direct arylation using a reported procedure [33], afforded the 2-aryl-5-bromoselenophenes **15**–**17** in 84–90% yields. Then, a second Pd-catalyzed direct arylation using heteroarenes and **15**–**17** as reaction partners, provided the target compounds **18**–**26** in high yields. Both thiazole and thiophene derivatives were successfully employed in this transformation. The reaction tolerates useful functional groups on both coupling partners such as nitrile, acetyl or chloro. It should be mentioned that again a regioselective arylation at the C2-position of 3-chlorothiophene was observed affording **26** in 72% yield.

**Scheme 5:**
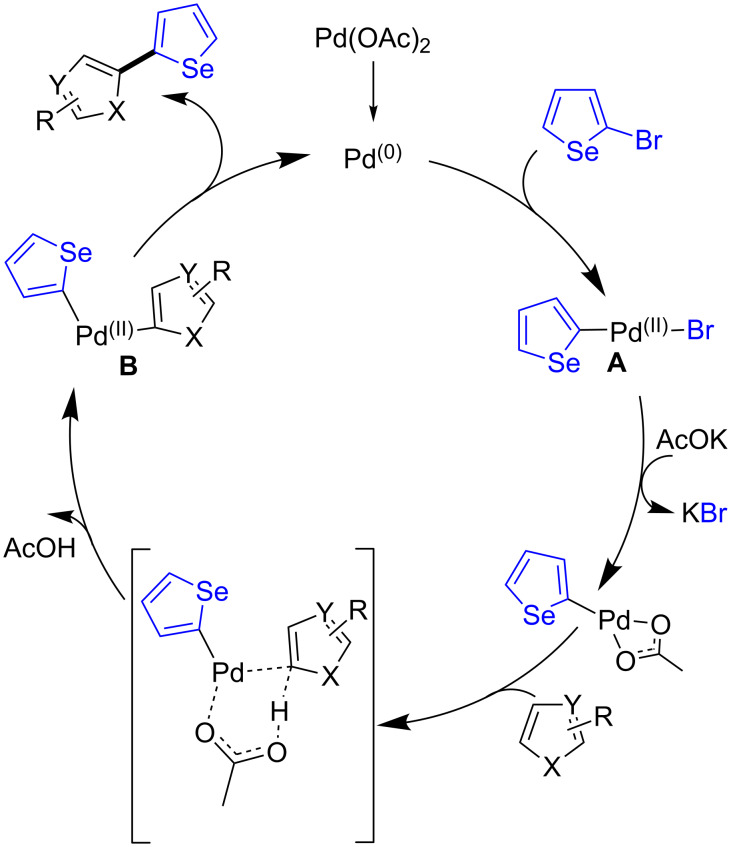
Proposed catalytic cycle.

Although the mechanism of these reactions was not elucidated, the catalytic cycle shown on Scheme 5 can be proposed. The first step is probably the oxidative addition of the 2-bromoselenophene to Pd(0) to afford the Pd(II) intermediate **A**. Then, after elimination of KBr with KOAc, a concerted metalation–deprotonation pathway involving an heteroarene gives **B**. Reductive elimination affords the 2-heteroarylated selenophene with regeneration of the Pd(0) species.

## Conclusion

In summary, the reactivity of 2-bromoselenophenes was investigated and revealed that the C2-heteroarylation of 2-bromoselenophene in high yields is only possible with specific heteroarenes such as thiazoles and imidazopyridines, whereas thiophene or pyrroles gave the desired products in low yields. Conversely, 2,5-dibromoselenophene was successfully coupled with both thiazoles and thiophenes in the presence of phosphine-free Pd(OAc)_2_ catalyst precursor and KOAc as inexpensive base, affording the desired 2,5-diheteroarylated selenophenes in high yields. We also described that the sequential catalytic C2-arylation, bromination, and catalytic C5-arylation of selenophene provides the controled double (hetero)arylation at the C2 and C5 positions of selenophene in good yields.

## Experimental

### General procedure for palladium-catalyzed direct mono-heteroarylations of 2-bromoselenophene

The reaction of the heteroarene (1.5 mmol), 2-bromoselenophene (0.210 g, 1 mmol) and KOAc (0.196 g, 2 mmol) at 90 °C during 24 h in DMA (4 mL) in the presence of Pd(OAc)_2_ (4.5 mg, 0.02 mmol), under argon affords the coupling products **1**–**7** after evaporation of the solvent and purification on silica gel. Eluents: Pentane for compounds **3** and **4**. EtOAc/pentane 2:98 for compounds **1** and **6**. EtOAc/pentane 10:90 for compound **2** and **5** and EtOAc/pentane 40:60 for compound **7**.

### General procedure for palladium-catalyzed direct diheteroarylations

The reaction of the heteroarene (3 mmol), 2,5-dibromoselenophene (0.289 g, 1 mmol) and KOAc (0.392 g, 4 mmol) at 90 °C during 40 h in DMA (4 mL) in the presence of Pd(OAc)_2_ (4.5 mg, 0.02 mmol), under argon affords the coupling products **8**–**14** after evaporation of the solvent and purification on silica gel. Eluents: Pentane for compounds **10**–**13**. EtOAc/pentane 5:95 for compounds **8, 9** and **14**.

### General procedure for the synthesis of 5-bromo-2-arylselenophenes **15–17**

To a mixture of the 2-arylselenophene [2] (2 mmol) in DMF (5 mL) at 0 °C, *N*-bromosuccinimide (0.392 g, 2.2 mmol) was slowly added. Then, the mixture was allowed to increase to room temperature and stirred during 20 h. After addition of water, the extraction was carried out with diethyl ether. Then, the organic phase was dried over magnesium sulphate. Finally, evaporation of the solvent and purification on silica gel afforded the 5-bromo-2-arylselenophenes **15**–**17**. Eluents: Pentane for compounds **15** and **16**. EtOAc:pentane 5:95 for compound **17**.

### General procedure for palladium-catalyzed direct mono-heteroarylations of 2-bromo-5-arylselenophenes

The reaction of the heteroarene (1.2 mmol), 2-bromo-5-arylselenophene **15**–**17** (1 mmol) and KOAc (0.392 g, 4 mmol) at 150 °C during 16 h in DMA (4 mL) in the presence of Pd(OAc)_2_ (4.5 mg, 0.02 mmol), under argon affords the coupling products **18**–**26** after evaporation of the solvent and purification on silica gel. Eluents: Pentane for compounds **21** and **22**. EtOAc/pentane 5:95 for compounds **18** and **19**. EtOAc/pentane 10:90 for compounds **20**, **23**, **24** and **26**. EtOAc/pentane 20:80 for compound **25**.

## Supporting Information

File 1 Additional experimental and analytical data and copies of NMR spectra.
