# Supplementary material for: Reactivity of bromoselenophenes in palladium-catalyzed direct arylations
Source: Beilstein J Org Chem. 2017 Dec 22;13:2862–8. doi: 10.3762/bjoc.13.278 (PMC5753141; doi:10.3762/bjoc.13.278)

Supporting Information

for

Reactivity of bromoselenophenes in palladium-catalyzed direct arylations

Aymen Skhiri[1,2], Ridha Ben Salem*[2], Jean-François Soulé*[1] and Henri Doucet*[1]

Address: [1]Institut des Sciences Chimiques de Rennes, UMR 6226 CNRS-Université de Rennes 1, "Organométalliques: Matériaux et Catalyse", Campus de Beaulieu, 35042 Rennes, France and [2]Laboratoire de Laboratoire de Chimie Organique LR 17ES08, Université de Sfax, Faculté des Sciences de Sfax, Route de la Soukra km 4, 3038 Sfax, Tunisia

Email: Ridha Ben Salem - ridhabensalem@yahoo.fr; Jean-François Soulé - jean-francois.soule@univ-rennes1.fr; Henri Doucet - henri.doucet@univ-rennes1.fr

*Corresponding author

Additional experimental and analytical data and copies of NMR spectra

General procedure for palladium-catalyzed direct mono-heteroarylations of 2-bromoselenophene

The reaction of the heteroarene (1.5 mmol), 2-bromoselenophene (0.210 g, 1 mmol) and KOAc (0.196 g, 2 mmol) at 90 °C during 24 h in DMA (4 mL) in the presence of $Pd(OAc)_2$ (4.5 mg, 0.02 mmol), under argon affords the coupling products **1**–**7** after evaporation of the solvent and purification on silica gel. Eluents: pentane for compounds **3** and **4**. EtOAc/pentane 2:98 for compounds **1** and **6**. EtOAc/pentane 10:90 for compound **2** and **5**. EtOAc/pentane 40:60 for compound **7**.

2-Ethyl-4-methyl-5-(selenophen-2-yl)thiazole (1)

From 2-bromoselenophene (0.210 g, 1 mmol) and 2-ethyl-4-methylthiazole (0.191 g, 1.5 mmol), **1** was obtained in 80% (0.205 g) yield; yellow oil.

^{1}H NMR (400 MHz, $CDCl_3$): δ 8.01 (dd, J = 5.6, 1.1 Hz, 1H), 7.29 (dd, J = 5.6, 3.8 Hz, 1H), 7.23 (dd, J = 3.8, 1.1 Hz, 1H), 2.97 (q, J = 7.6 Hz, 2H), 2.52 (s, 3H), 1.39 (t, J = 7.6 Hz, 3H).

^{13}C NMR (100 MHz, $CDCl_3$): δ 170.0, 147.4, 138.8, 131.5, 130.1, 128.9, 127.1, 27.0, 16.6, 14.3.

Elemental analysis: calcd (%) for $C_{10}H_{11}NSSe$ (256.23): C 46.88, H 4.33; found: C 46.99, H 4.17.

2-Isopropyl-4-methyl-5-(selenophen-2-yl)thiazole (2)

From 2-bromoselenophene (0.210 g, 1 mmol) and 2-isopropyl-4-methylthiazole (0.212 g, 1.5 mmol), **2** was obtained in 82% (0.221 g) yield; yellow oil.

^{1}H NMR (400 MHz, $CDCl_3$): δ 8.00 (dd, J = 5.6, 1.1 Hz, 1H), 7.29 (dd, J = 5.6, 3.8 Hz, 1H), 7.23 (dd, J = 3.8, 1.1 Hz, 1H), 3.25 (sept., J = 7.6 Hz, 1H), 2.53 (s, 3H), 1.39 (d, J = 7.6 Hz, 6H).

^{13}C NMR (100 MHz, $CDCl_3$): δ 175.1, 147.3, 138.9, 131.5, 130.1, 128.9, 126.7, 33.5, 23.3, 16.7.

Elemental analysis: calcd (%) for $C_{11}H_{13}NSSe$ (270.25): C 48.89, H 4.85; found: C 48.80, H 4.82.

5-(Selenophen-2-yl)thiophene-2-carbonitrile (3)

From 2-bromoselenophene (0.210 g, 1 mmol) and thiophene-2-carbonitrile (0.164 g, 1.5 mmol), **3** was obtained in 24% (0.057 g) yield; amorphous brown solid; mp 56–58 °C.

^{1}H NMR (400 MHz, $CDCl_3$): δ 8.04 (d, J = 5.6 Hz, 1H), 7.51 (d, J = 3.9 Hz, 1H), 7.45 (d, J = 3.8 Hz, 1H), 7.29 (dd, J = 5.6, 3.8 Hz, 1H), 7.08 (d, J = 3.9 Hz, 1H).

^{13}C NMR (100 MHz, $CDCl_3$): δ 147.0, 139.8, 138.4, 132.7, 130.8, 128.5, 124.2, 114.3, 107.8.

Elemental analysis: calcd (%) for C_9H_5NSSe (238.17): C 45.39, H 2.12; found: C 45.19, H 2.17.

2-Chloro-5-(selenophen-2-yl)thiophene (4)

From 2-bromoselenophene (0.210 g, 1 mmol) and 2-chlorothiophene (0.178 g, 1.5 mmol), **4** was obtained in 32% (0.079 g) yield; amorphous yellow solid; mp 38–40 °C.

^{1}H NMR (400 MHz, $CDCl_3$): δ 7.91 (dd, J = 5.6, 1.6 Hz, 1H), 7.30-7.23 (m, 2H), 6.91 (d, J = 3.9 Hz, 1H), 6.83 (d, J = 3.9 Hz, 1H).

^{13}C NMR (100 MHz, $CDCl_3$): δ 141.7, 138.4, 130.4, 130.1, 129.0, 127.0, 126.2, 123.6.

Elemental analysis: calcd (%) for C_8H_5ClSSe (247.60): C 38.81, H 2.04; found: C 38.99, H 1.88.

2-Pentyl-5-(selenophen-2-yl)thiophene (5)

From 2-bromoselenophene (0.210 g, 1 mmol) and 2-pentylthiophene (0.231 g, 1.5 mmol), **5** was obtained in 14% (0.099 g) yield; yellow oil.

^{1}H NMR (400 MHz, $CDCl_3$): δ 7.81 (dd, *J* = 5.4, 1.2 Hz, 1H), 7.26-7.18 (m, 2H), 6.93 (d, *J* = 3.6 Hz, 1H), 6.66 (d, *J* = 3.6 Hz, 1H), 2.77 (t, *J* = 7.6 Hz, 2H), 1.76-1.70 (m, 2H), 1.43-1.35 (m, 4H), 0.94 (t, *J* = 7.6 Hz, 3H).

^{13}C NMR (100 MHz, $CDCl_3$): δ 145.8, 143.2, 137.2, 130.3, 128.9, 125.1, 124.9, 124.2, 31.4, 30.3, 22.6, 14.1.

Elemental analysis: calcd (%) for $C_{13}H_{16}SSe$ (283.29): C 55.12, H 5.69; found: C 55.00, H 5.47.

1-Phenyl-2-(selenophen-2-yl)-pyrrole (6)

From 2-bromoselenophene (0.210 g, 1 mmol) and 1-phenylpyrrole (0.286 g, 2 mmol), **6** was obtained in 15% (0.041g) yield; amorphous brown solid; mp 74–76 °C.

^{1}H NMR (400 MHz, $CDCl_3$): δ 7.77 (dd, *J* = 5.6, 1.1 Hz, 1H), 7.44-7.35 (m, 3H), 7.33-7.27 (m, 2H), 7.09 (dd, *J* = 5.6, 3.8 Hz, 1H), 6.88 (dd, *J* = 2.8, 1.8 Hz, 1H), 6.81 (dd, *J* = 3.8, 1.1 Hz, 1H), 6.48 (dd, *J* = 3.6, 1.8 Hz, 1H), 6.31 (dd, *J* = 3.6, 2.8 Hz, 1H).

^{13}C NMR (100 MHz, $CDCl_3$): δ 140.2, 140.0, 129.9, 129.7, 129.6, 129.2, 127.8, 127.1, 126.8, 124.7, 111.0, 109.3.

Elemental analysis: calcd (%) for $C_{14}H_{11}NSe$ (272.21): C 61.77, H 4.07; found: C 61.49, H 4.31.

3-(Selenophen-2-yl)imidazo[1,2-*a*]pyridine (7)

From 2-bromoselenophene (0.210 g, 1 mmol) and imidazo[1,2-*a*]pyridine (0.177 g, 1.5 mmol), **7** was obtained in 81% (0.201 g) yield; yellow oil.

^{1}H NMR (400 MHz, $CDCl_3$): δ 8.41 (dd, *J* = 6.9 Hz, 1H), 8.12 (dd, *J* = 5.6, 1.2 Hz, 1H), 7.75 (s, 1H), 7.66 (d, *J* = 8.9 Hz, 1H), 7.45-7.37 (m, 2H), 7.22 (ddd, *J* = 8.9, 6.9, 1.1 Hz, 1H), 6.88 (td, *J* = 6.8, 1.2 Hz, 1H).

^{13}C NMR (100 MHz, $CDCl_3$): δ 146.5, 134.9, 133.8, 131.8, 130.4, 128.4, 124.7, 123.9, 121.3, 118.3, 113.1.

Elemental analysis: calcd (%) for $C_{11}H_8N_2Se$ (247.16): C 53.46, H 3.26; found: C 53.50, H 3.27.

General procedure for palladium-catalyzed direct di-heteroarylations

The reaction of the heteroarene (3 mmol), 2,5-dibromoselenophene (0.289 g, 1 mmol) and KOAc (0.392 g, 4 mmol) at 90 °C during 40 h in DMA (4 mL) in the presence of $Pd(OAc)_2$ (4.5 mg, 0.02 mmol), under argon

affords the coupling products **8**–**14** after evaporation of the solvent and purification on silica gel. Eluents: Pentane for compounds **10**–**13**. EtOAc/pentane 5:95 for compounds **8, 9** and **14**.

2,5-Bis(2,4-dimethylthiazol-5-yl)selenophene (8)

From 2,5-dibromoselenophene (0.289 g, 1 mmol) and 2,4-dimethylthiazole (0.336 g, 3 mmol), **8** was obtained in 78% (0.275 g) yield; amorphous orange solid; mp 112–114°C.

^{1}H NMR (400 MHz, $CDCl_3$): δ 7.14 (s, 2H), 2.64 (s, 6H), 2.51 (s, 6H).

^{13}C NMR (100 MHz, $CDCl_3$): δ 163.2, 147.7, 139.1, 129.0, 127.2, 19.2, 16.7.

Elemental analysis: calcd (%) for $C_{14}H_{14}N_2S_2Se$ (353.36): C 47.59, H 3.99; found: C 47.64, H 4.11.

2,5-Bis(2-isopropyl-4-methylthiazol-5-yl)selenophene (9)

From 2,5-dibromoselenophene (0.289 g, 1 mmol) and 2-isoproyl-4-methylthiazole (0.423 g, 3 mmol), **9** was obtained in 80% (0.327 g) yield; green oil.

^{1}H NMR (400 MHz, $CDCl_3$): δ 7.16 (s, 2H), 3.24 (sept., *J* = 7.6 Hz, 2H), 2.53 (s, 6H), 1.38 (d, *J* = 7.6 Hz, 12H).

^{13}C NMR (100 MHz, $CDCl_3$): δ 175.2, 147.5, 139.3, 128.9, 126.4, 33.5, 23.2, 16.8.

Elemental analysis: calcd (%) for $C_{18}H_{22}N_2S_2Se$ (409.47): C 52.80, H 5.42; found: C 52.74, H 5.19.

2,5-Bis(5-pentylthiophen-2-yl)selenophene (10)

From 2,5-dibromoselenophene (0.289 g, 1 mmol) and 2-pentylthiophene (0.462 g, 3 mmol), **10** was obtained in 74% (0.322 g) yield; amorphous brown solid; mp 112–114 °C.

^{1}H NMR (400 MHz, $CDCl_3$): δ 7.13 (s, 2H), 6.92 (d, *J* = 3.5 Hz, 2H), 6.68 (d, *J* = 3.5 Hz, 2H), 2.80 (t, *J* = 7.6 Hz, 4H), 1.76-1.70 (m, 4H), 1.43-1.35 (m, 8H), 0.94 (t, *J* = 7.6 Hz, 6H).

^{13}C NMR (100 MHz, $CDCl_3$): δ 145.8, 141.0, 137.1, 125.6, 125.0, 123.9, 31.4 (2C), 30.3, 22.6, 14.1.

Elemental analysis: calcd (%) for $C_{22}H_{28}S_2Se$ (435.55): C 60.67, H 6.48; found: C 60.80, H 6.37.

2,5-Bis(5-chlorothiophen-2-yl)selenophene (11)

From 2,5-dibromoselenophene (0.289 g, 1 mmol) and 2-chlorothiophene (0.355 g, 3 mmol), **11** was obtained in 71% (0.258 g) yield; amorphous yellow solid; mp 194–196 °C.

^{1}H NMR (400 MHz, $CDCl_3$): δ 7.12 (s, 2H), 6.86 (d, *J* = 3.5 Hz, 2H), 6.82 (d, *J* = 3.5 Hz, 2H).

^{13}C NMR (100 MHz, $CDCl_3$): δ 140.6, 137.8, 129.4, 127.2, 126.6, 123.7.

Elemental analysis: calcd (%) for $C_{12}H_6Cl_2S_2Se$ (364.16): C 39.58, H 1.66; found: C 39.40, H 1.80.

2,5-Bis(3-methylthiophen-2-yl)selenophene (12) [1]

From 2,5-dibromoselenophene (0.289 g, 1 mmol) and 3-methylthiophene (0.294 g, 3 mmol), **12** was obtained in 69% (0.223 g) yield; amorphous brown solid; mp 78–80 °C.

^{1}H NMR (400 MHz, $CDCl_3$): δ 7.26 (s, 2H), 7.13 (d, *J* = 5.2 Hz, 2H), 6.88 (d, *J* = 5.2 Hz, 2H), 2.40 (s, 6H).

^{13}C NMR (100 MHz, $CDCl_3$): δ 141.5, 133.8, 133.0, 131.7, 127.8, 123.4, 15.7.

2,5-Bis(3-chlorothiophen-2-yl)selenophene (13)

From 2,5-dibromoselenophene (0.289 g, 1 mmol) and 3-chlorothiophene (0.355 g, 3 mmol), **13** was obtained in 72% (0.262 g) yield; amorphous yellow solid; mp 150–152 °C.

^{1}H NMR (400 MHz, $CDCl_3$): δ 7.49 (s, 2H), 7.17 (d, *J* = 5.4 Hz, 2H), 6.96 (d, *J* = 5.4 Hz, 2H).

^{13}C NMR (100 MHz, $CDCl_3$): δ 138.9, 133.0, 129.4, 127.8, 123.2, 121.7.

Elemental analysis: calcd (%) for $C_{12}H_6Cl_2S_2Se$ (364.16): C 39.58, H 1.66; found: C 39.71, H 1.48.

2,5-Bis(1-methylpyrrol-2-yl)selenophene (14)

From 2,5-dibromoselenophene (0.289 g, 1 mmol) and 1-methylpyrrole (0.648 g, 8 mmol), **14** was obtained in 81% (0.235 g) yield; yelow oil.

^{1}H NMR (500 MHz, $CDCl_3$): δ 7.13 (s, 2H), 6.74-6.71 (m, 2H), 6.35 (dd, *J* = 3.7, 1.8 Hz, 2H), 6.18 (dd, *J* = 3.7, 2.7 Hz, 2H), 3.78 (s, 6H).

^{13}C NMR (125 MHz, $CDCl_3$): δ 139.3, 129.0, 127.0, 124.2, 110.3, 108.0, 35.3.

Elemental analysis: calcd (%) for $C_{14}H_{14}N_2Se$ (290.24): C 58.14, H 4.88; found: C 58.29, H 5.12.

General procedure for the synthesis of 5-bromo-2-arylselenophenes 15–17

To a mixture of the 2-arylselenophene [2] (2 mmol) in DMF (5 mL) at 0 °C, *N*-bromosuccinimide (0.392 g, 2.2 mmol) was slowly added. Then, the mixture was allowed to warm to room temperature and stirred for 20 h. After addition of water, the extraction was carried out with diethyl ether. Then, the organic phase was dried over magnesium sulfate. Finally, evaporation of the solvent and purification on silica gel afforded the 5-bromo-2-arylselenophenes **15–17**. Eluents: pentane for compounds **15** and **16**. EtOAc/pentane 5:95 for compound **17**.

4-(5-Bromoselenophen-2-yl)benzonitrile (15) [3]

From 4-(selenophen-2-yl)benzonitrile (0.464 g, 2 mmol) and *N*-bromosuccinimide (0.392 g, 2.2 mmol), **15** was obtained in 90% (0.560 g) yield; amorphous white solid; mp 116–118 °C.

^{1}H NMR (400 MHz, $CDCl_3$): δ 7.63 (d, *J* = 8.5 Hz, 2H), 7.53 (d, *J* = 8.5 Hz, 2H), 7.28 (d, *J* = 4.1 Hz, 1H), 7.27 (d, *J* = 4.1 Hz, 1H).

^{13}C NMR (100 MHz, $CDCl_3$): δ 150.0, 140.0, 134.5, 133.0, 127.4, 126.4, 118.8, 117.7, 111.2.

2-Bromo-5-(4-chlorophenyl)selenophene (16)

From 2-(4-chlorophenyl)selenophene (0.481 g, 2 mmol) and *N*-bromosuccinimide (0.392 g, 2.2 mmol), **16** was obtained in 88% (0.564 g) yield; amorphous white solid; mp 113–115 °C.

^{1}H NMR (400 MHz, $CDCl_3$): δ 7.37 (d, *J* = 8.5 Hz, 2H), 7.32 (d, *J* = 8.5 Hz, 2H), 7.23 (d, *J* = 4.1 Hz, 1H), 7.12 (d, *J* = 4.1 Hz, 1H).

^{13}C NMR (100 MHz, $CDCl_3$): δ 151.2, 134.4, 134.2, 133.9, 129.3, 127.3, 125.7, 115.2.

1-(4-(5-Bromoselenophen-2-yl)phenyl)ethan-1-one (17)

From 1-(4-(selenophen-2-yl)phenyl)ethan-1-one (0.498 g, 2 mmol) and *N*-bromosuccinimide (0.392 g, 2.2 mmol), **17** was obtained in 84% (0.550 g) yield; amorphous white solid; mp 155–157 °C.

^{1}H NMR (400 MHz, $CDCl_3$): δ 7.93 (d, *J* = 8.5 Hz, 2H), 7.52 (d, *J* = 8.5 Hz, 2H), 7.28 (s, 2H), 2.60 (s, 3H).

^{13}C NMR (100 MHz, $CDCl_3$): δ 197.3, 151.0, 140.1, 136.3, 134.4, 129.4, 126.8, 126.0, 116.8, 26.7.

General procedure for palladium-catalyzed direct mono-heteroarylations of 2-bromo-5-arylselenophenes

The reaction of the heteroarene (1.2 mmol), 2-bromo-5-arylselenophene **15**–**17** (1 mmol) and KOAc (0.392 g, 4 mmol) at 150 °C during 16 h in DMA (4 mL) in the presence of $Pd(OAc)_2$ (4.5 mg, 0.02 mmol), under argon affords the coupling products **18**–**26** after evaporation of the solvent and purification on silica gel. Eluents: Pentane for compounds **21** and **22**. EtOAc/pentane 5:95 for compounds **18** and **19**. EtOAc/pentane 10:90 for compounds **20**, **23**, **24** and **26**. EtOAc/pentane 20:80 for compound **25**.

4-(5-(2-Ethyl-4-methylthiazol-5-yl)selenophen-2-yl)benzonitrile (18)

From 4-(5-bromoselenophen-2-yl)benzonitrile (**15**, 0.311 g, 1 mmol) and 2-ethyl-4-methylthiazole (0.152 g, 1.2 mmol), **18** was obtained in 81% (0.289 g) yield; amorphous yellow solid; mp 132–134 °C.

^{1}H NMR (400 MHz, $CDCl_3$): δ 7.64 (d, *J* = 8.5 Hz, 2H), 7.60 (d, *J* = 8.5 Hz, 2H), 7.52 (d, *J* = 4.1 Hz, 1H), 7.24 (d, *J* = 4.1 Hz, 1H), 2.98 (q, *J* = 7.6 Hz, 2H), 2.56 (s, 3H), 1.39 (t, *J* = 7.6 Hz, 3H).

^{13}C NMR (100 MHz, $CDCl_3$): δ 170.5, 148.3, 148.2, 140.7, 140.3, 132.9, 129.9, 127.9, 126.8, 126.3, 118.9, 110.9, 27.1, 16.9, 14.2.

Elemental analysis: calcd (%) for $C_{17}H_{14}N_2SSe$ (357.33): C 57.14, H 3.65; found: C 57.01, H 3.87.

5-(5-(4-Chlorophenyl)selenophen-2-yl)-2-ethyl-4-methylthiazole (19)

From 2-bromo-5-(4-chlorophenyl)selenophene (**16**, 0.320 g, 1 mmol) and 2-ethyl-4-methylthiazole (0.152 g, 1.2 mmol), **19** was obtained in 80% (0.293 g) yield; amorphous yellow solid; mp 110–112 °C.

^{1}H NMR (400 MHz, $CDCl_3$): δ 7.46 (d, *J* = 8.5 Hz, 2H), 7.38 (d, *J* = 4.1 Hz, 1H), 7.33 (d, *J* = 8.5 Hz, 2H), 7.20 (d, *J* = 4.1 Hz, 1H), 2.97 (q, *J* = 7.6 Hz, 2H), 2.55 (s, 3H), 1.39 (t, *J* = 7.6 Hz, 3H).

^{13}C NMR (100 MHz, $CDCl_3$): δ 169.9, 149.6, 147.6, 138.3, 134.5, 133.6, 129.7, 129.2, 127.2, 127.0, 125.9, 26.9, 16.7, 14.2.

Elemental analysis: calcd (%) for $C_{16}H_{14}ClNSSe$ (366.77): C 52.40, H 3.85; found: C 52.17, H 3.87.

1-(4-(5-(2-Ethyl-4-methylthiazol-5-yl)selenophen-2-yl)phenyl)ethan-1-one (20)

From 1-(4-(5-bromoselenophen-2-yl)phenyl)ethan-1-one (**17**, 0.328 g, 1 mmol) and 2-ethyl-4-methylthiazole (0.152 g, 1.2 mmol), **20** was obtained in 87% (0.325 g) yield; amorphous yellow solid; mp 90-96°C.

^{1}H NMR (400 MHz, $CDCl_3$): δ 7.94 (d, *J* = 8.5, Hz, 2H), 7.60 (d, *J* = 8.5, Hz, 2H), 7.52 (d, *J* = 4.0 Hz, 1H), 7.23 (d, *J* = 4.0 Hz, 1H), 2.97 (q, *J* = 7.6 Hz, 2H), 2.59 (s, 3H), 2.55 (s, 3H), 1.39 (t, *J* = 7.6 Hz, 3H).

^{13}C NMR (100 MHz, $CDCl_3$): δ 197.3, 170.3, 149.4, 147.9, 140.4, 139.8, 136.0, 129.9, 129.3, 127.3, 127.0, 125.9, 27.0, 26.7, 16.8, 14.3.

Elemental analysis: calcd (%) for $C_{18}H_{17}NOSSe$ (374.36): C 57.75, H 4.58; found: C 57.89, H 4.41.

4-(5-(5-Chlorothiophen-2-yl)selenophen-2-yl)benzonitrile (21)

From 4-(5-bromoselenophen-2-yl)benzonitrile (**15**, 0.311 g, 1 mmol) and 2-chlorothiophene (0.142 g, 1.2 mmol), **21** was obtained in 69% (0.240 g) yield; amorphous yellow solid; mp 192-194°C.

^{1}H NMR (400 MHz, $CDCl_3$): δ 7.65 (d, *J* = 8.2 Hz, 2H), 7.57 (d, *J* = 8.2 Hz, 2H), 7.48 (d, *J* = 4.0 Hz, 1H), 7.24 (d, *J* = 4.0 Hz, 1H), 6.94 (d, *J* = 3.9 Hz, 1H), 6.85 (d, *J* = 3.9 Hz, 1H).

^{13}C NMR (100 MHz, $CDCl_3$): δ 146.9, 143.3, 140.2, 137.7, 130.0, 128.2, 127.3, 127.2, 126.4, 126.3, 124.2, 118.9, 110.9.

Elemental analysis: calcd (%) for $C_{15}H_8ClNSSe$ (348.71): C 51.67, H 2.31; found: C 51.41, H 2.17.

2-Chloro-5-(5-(4-chlorophenyl)selenophen-2-yl)thiophene (22)

From 2-bromo-5-(4-chlorophenyl)selenophene (**16**, 0.320 g, 1 mmol) and 2-chlorothiophene (0.142 g, 1.2 mmol), **22** was obtained in 58% (0.207 g) yield; amorphous yellow solid; mp 208–210 °C.

^{1}H NMR (400 MHz, $CDCl_3$): δ 7.45 (d, *J* = 8.2 Hz, 2H), 7.38-7.31 (m, 3H), 7.20 (d, *J* = 4.0 Hz, 1H), 6.90 (d, *J* = 3.9 Hz, 1H), 6.83 (d, *J* = 3.9 Hz, 1H).

^{13}C NMR (100 MHz, $CDCl_3$): δ 148.4, 141.2, 138.2, 134.6, 133.7, 129.3, 127.3, 127.2, 127.1, 126.4, 123.6.

Elemental analysis: calcd (%) for $C_{14}H_8Cl_2SSe$ (358.14): C 46.95, H 2.25; found: C 46.78, H 2.03.

1-(4-(5-(5-Chlorothiophen-2-yl)selenophen-2-yl)phenyl)ethan-1-one (23)

From 1-(4-(5-bromoselenophen-2-yl)phenyl)ethan-1-one (**17**, 0.328 g, 1 mmol) and 2-chlorothiophene (0.142 g, 1.2 mmol), **23** was obtained in 84% (0.307 g) yield; amorphous yellow solid; mp 204–206 °C.

^{1}H NMR (400 MHz, $CDCl_3$): δ 7.99 (d, *J* = 8.5, Hz, 2H), 7.60 (d, *J* = 8.5, Hz, 2H), 7.49 (d, *J* = 4.0 Hz, 1H), 7.24 (d, *J* = 4.0 Hz, 1H), 6.93 (d, *J* = 3.9 Hz, 1H), 6.84 (d, *J* = 3.9 Hz, 1H), 2.61 (s, 3H).

^{13}C NMR (100 MHz, $CDCl_3$): δ 197.3, 148.0, 142.5, 140.3, 138.0, 136.1, 129.7, 129.3, 127.6, 127.3, 127.2, 126.0, 123.9, 26.7.

Elemental analysis: calcd (%) for $C_{16}H_{11}ClOSSe$ (365.73): C 52.55, H 3.03; found: C 52.70, H 3.20.

1-(4-(5-(5-Pentylthiophen-2-yl)selenophen-2-yl)phenyl)ethan-1-one (24)

From 1-(4-(5-bromoselenophen-2-yl)phenyl)ethan-1-one (**17**, 0.328 g, 1 mmol) and 2-pentylthiophene (0.185 g, 1.2 mmol), **24** was obtained in 77% (0.309 g) yield; amorphous yellow solid; mp 199–201 °C.

^{1}H NMR (400 MHz, $CDCl_3$): δ 7.94 (d, *J* = 8.5, Hz, 2H), 7.59 (d, *J* = 8.5, Hz, 2H), 7.48 (d, *J* = 4.0 Hz, 1H), 7.23 (d, *J* = 4.0 Hz, 1H), 6.98 (d, *J* = 3.9 Hz, 1H), 6.69 (d, *J* = 3.9 Hz, 1H), 2.80 (t, *J* = 7.6 Hz, 2H), 2.61 (s, 3H), 1.75-1.65 (m, 2H), 1.42-1.32 (m, 4H), 0.91 (t, *J* = 7.6 Hz, 3H).

^{13}C NMR (100 MHz, $CDCl_3$): δ 197.3, 146.6, 146.5, 144.3, 140.7, 136.8, 135.8, 129.3, 127.7, 126.2, 125.8, 125.1, 124.6, 31.4, 31.3, 30.4, 26.7, 22.5, 14.1.

Elemental analysis: calcd (%) for $C_{21}H_{22}OSSe$ (401.43): C 62.83, H 5.52; found: C 62.98, H 5.67.

1-(4-(5-(5-Acetyl-3-chlorothiophen-2-yl)selenophen-2-yl)phenyl)ethan-1-one (25)

From 1-(4-(5-bromoselenophen-2-yl)phenyl)ethan-1-one (**17**, 0.328 g, 1 mmol) and 2-acetyl-4-chlorothiophene (0.193 g, 1.2 mmol), **25** was obtained in 80% (0.326 g) yield; amorphous yellow solid; mp 175–177 °C.

^{1}H NMR (400 MHz, $CDCl_3$): δ 7.95 (d, *J* = 8.5, Hz, 2H), 7.68 (d, *J* = 4.0 Hz, 1H), 7.64 (d, *J* = 8.5, Hz, 2H), 7.56 (d, *J* = 4.0 Hz, 1H), 7.51 (s, 1H), 2.61 (s, 3H), 2.53 (s, 3H).

^{13}C NMR (100 MHz, $CDCl_3$): δ 197.3, 189.6, 152.0, 140.8, 140.0, 139.5, 137.8, 136.4, 133.7, 131.1, 129.3, 126.9, 126.1, 121.8, 26.7, 26.4.

Elemental analysis: calcd (%) for $C_{18}H_{13}ClO_2SSe$ (407.77): C 53.02, H 3.21; found: C 52.80, H 3.04.

1-(4-(5-(3-Chlorothiophen-2-yl)selenophen-2-yl)phenyl)ethan-1-one (26)

From 1-(4-(5-bromoselenophen-2-yl)phenyl)ethan-1-one (**17**, 0.328 g, 1 mmol) and 3-chlorothiophene (0.142 g, 1.2 mmol), **26** was obtained in 72% (0.263 g) yield; amorphous yellow solid; mp 126–128 °C.

^{1}H NMR (400 MHz, $CDCl_3$): δ 7.95 (d, *J* = 8.5, Hz, 2H), 7.64 (d, *J* = 8.5, Hz, 2H), 7.55 (d, *J* = 4.1 Hz, 1H), 7.52 (d, *J* = 4.1 Hz, 1H), 7.18 (d, *J* = 5.4 Hz, 1H), 6.97 (d, *J* = 5.4 Hz, 1H), 2.61 (s, 3H).

^{13}C NMR (100 MHz, $CDCl_3$): δ 197.3, 149.4, 140.5, 139.2, 136.0, 132.8, 129.4, 129.3, 129.2, 126.8, 126.0, 123.4, 122.1, 26.7.

Elemental analysis: calcd (%) for $C_{16}H_{11}ClOSSe$ (365.73): C 52.55, H 3.03; found: C 52.31, H 2.84.

References

1. Zimmer, H.; Shabana, R.; Galal, A.; Mark, H. B., Jr.; Gronowitz, S.; Hoernfeldt, A. B. *Phosphorus, Sulfur Silicon Relat. Elem.* **1989**, *42*, 171-176.

2. Rampon, D. S.; Wessjohann, L. A.; Schneider, P. H. *J. Org. Chem.* **2014**, *79*, 5987-5992.

3. Ismail, M. A.; Boykin, D. W.; Stephens, C. E. *Tetrahedron Lett.* **2006**, *4*, 795-797.

2-Ethyl-4-methyl-5-(selenophen-2-yl)thiazole (1)

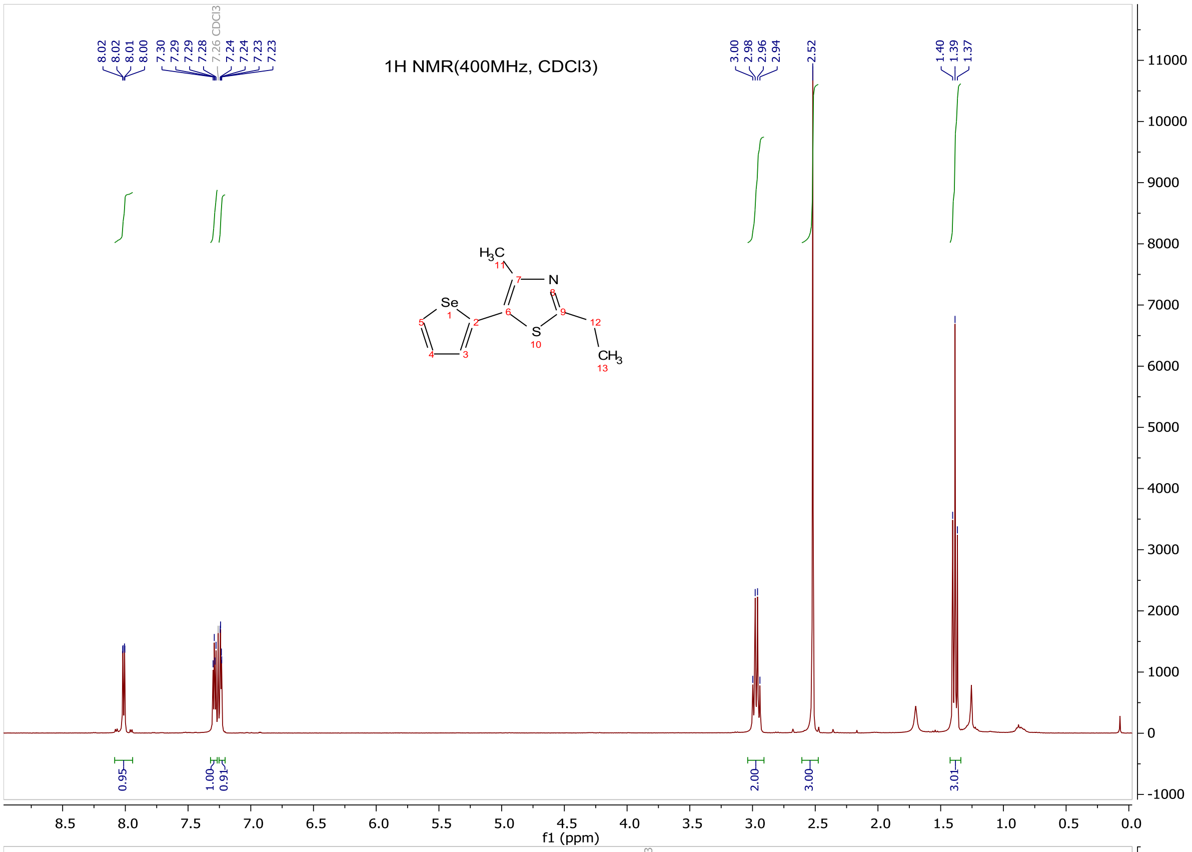

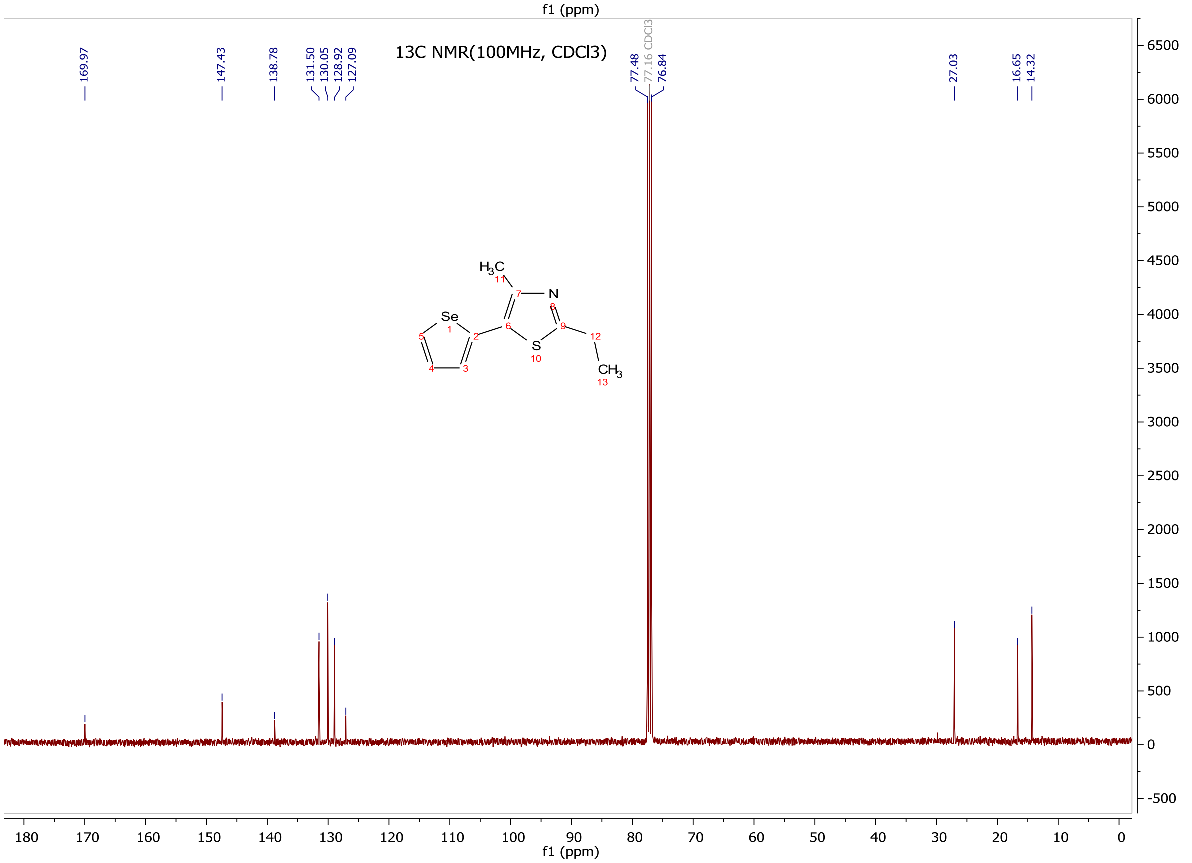

2-Isopropyl-4-methyl-5-(selenophen-2-yl)thiazole (2)

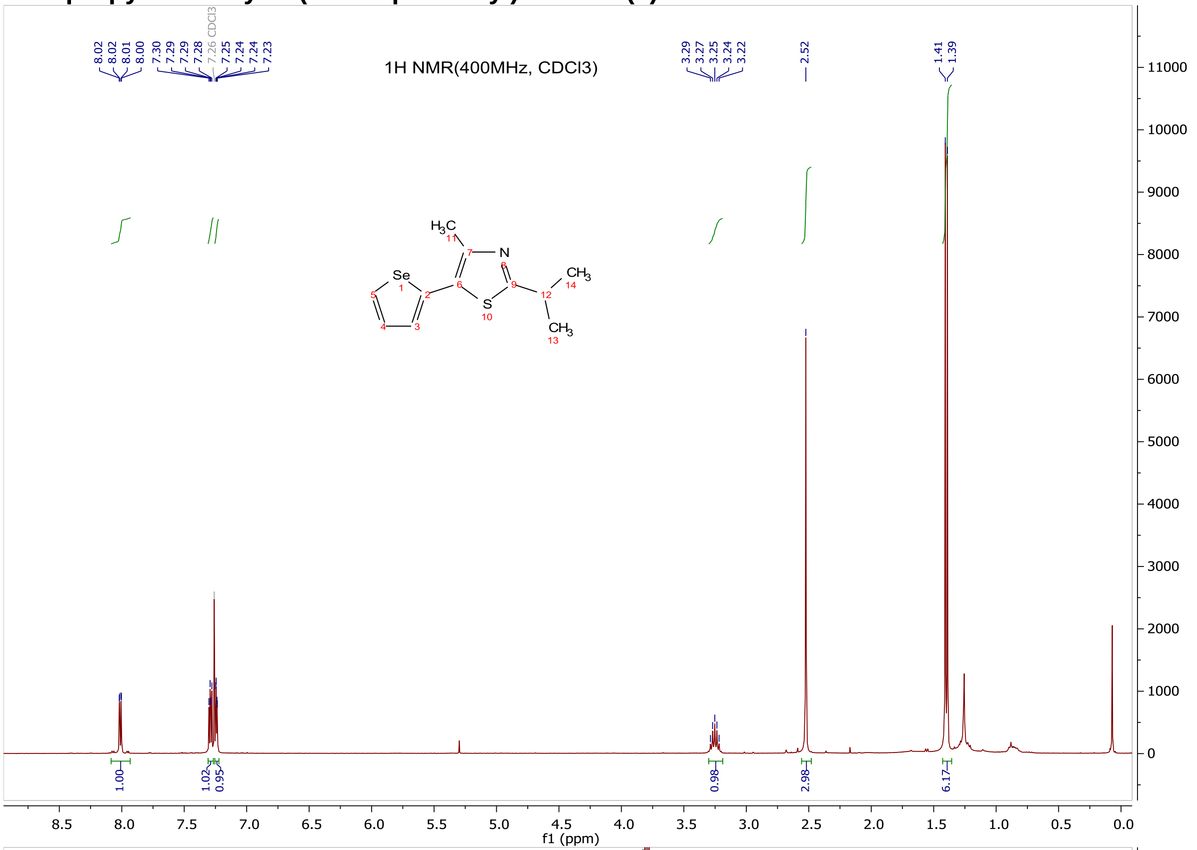

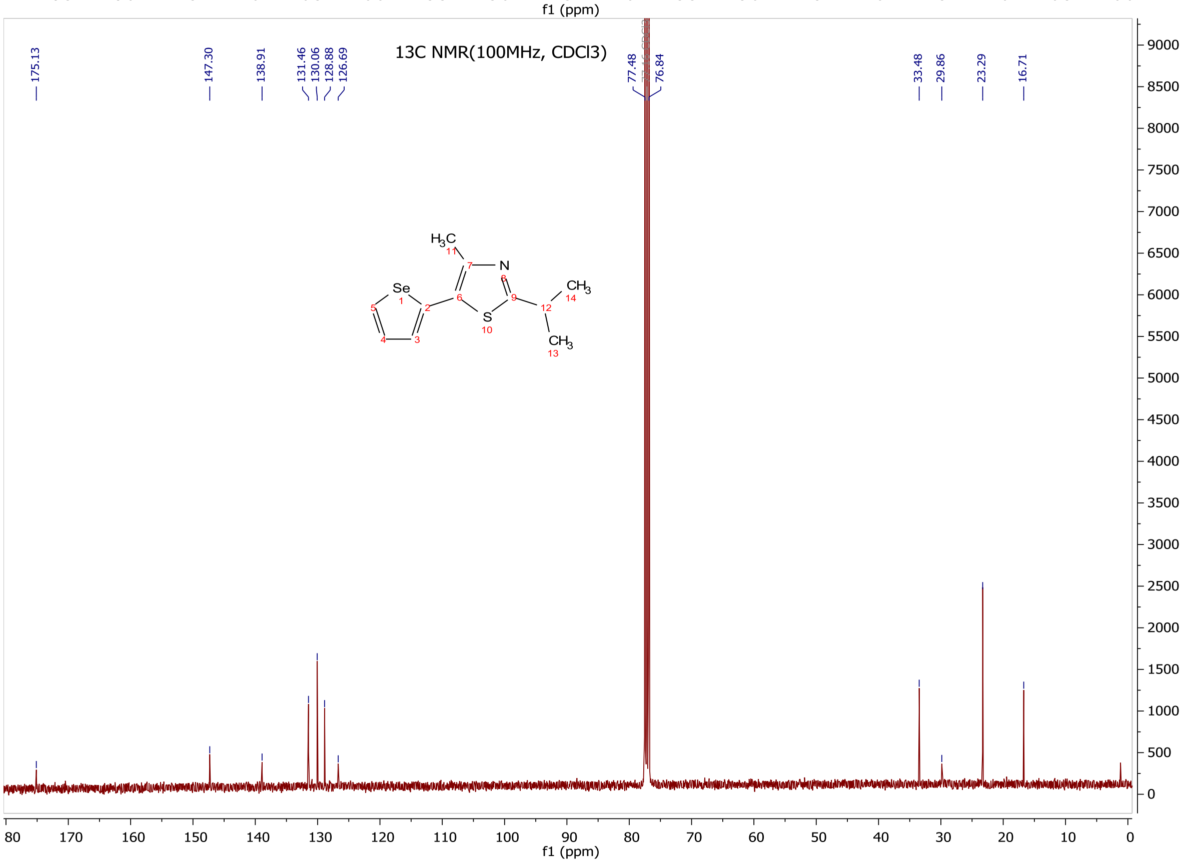

5-(Selenophen-2-yl)thiophene-2-carbonitrile (3)

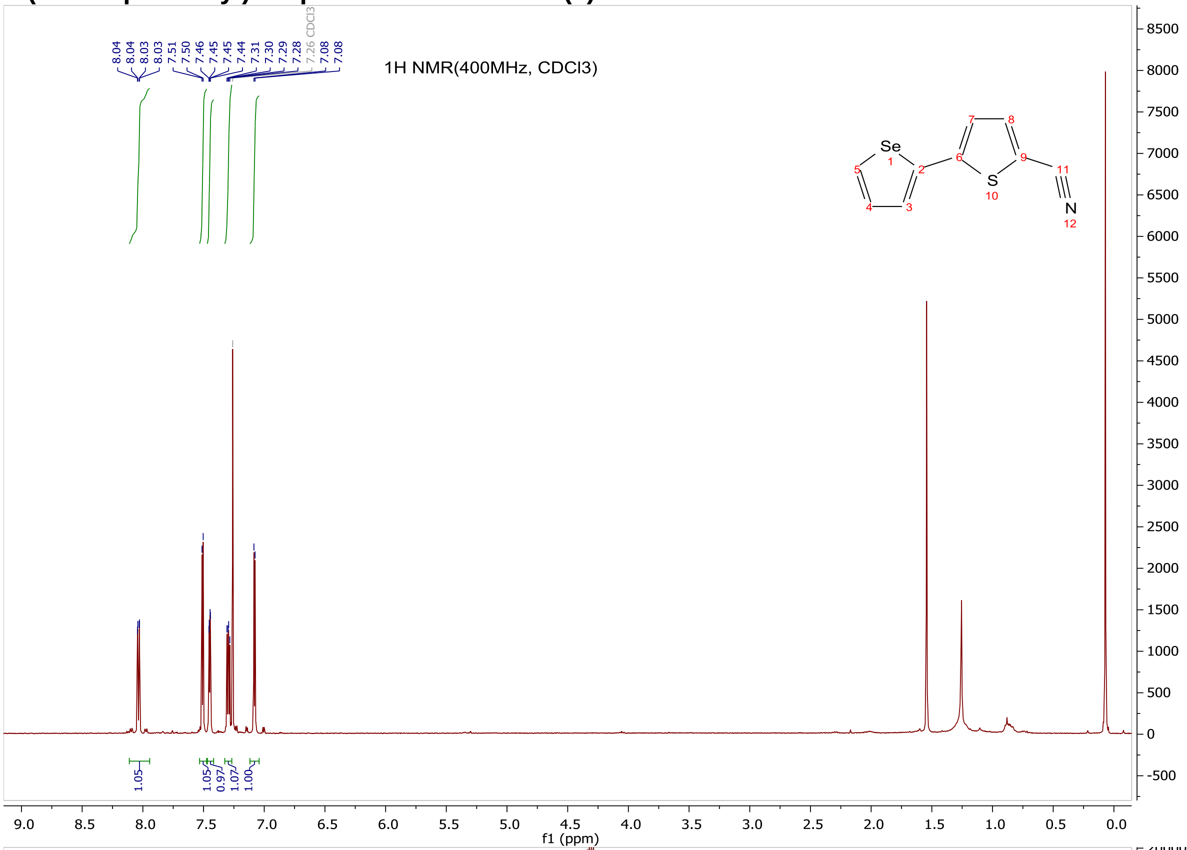

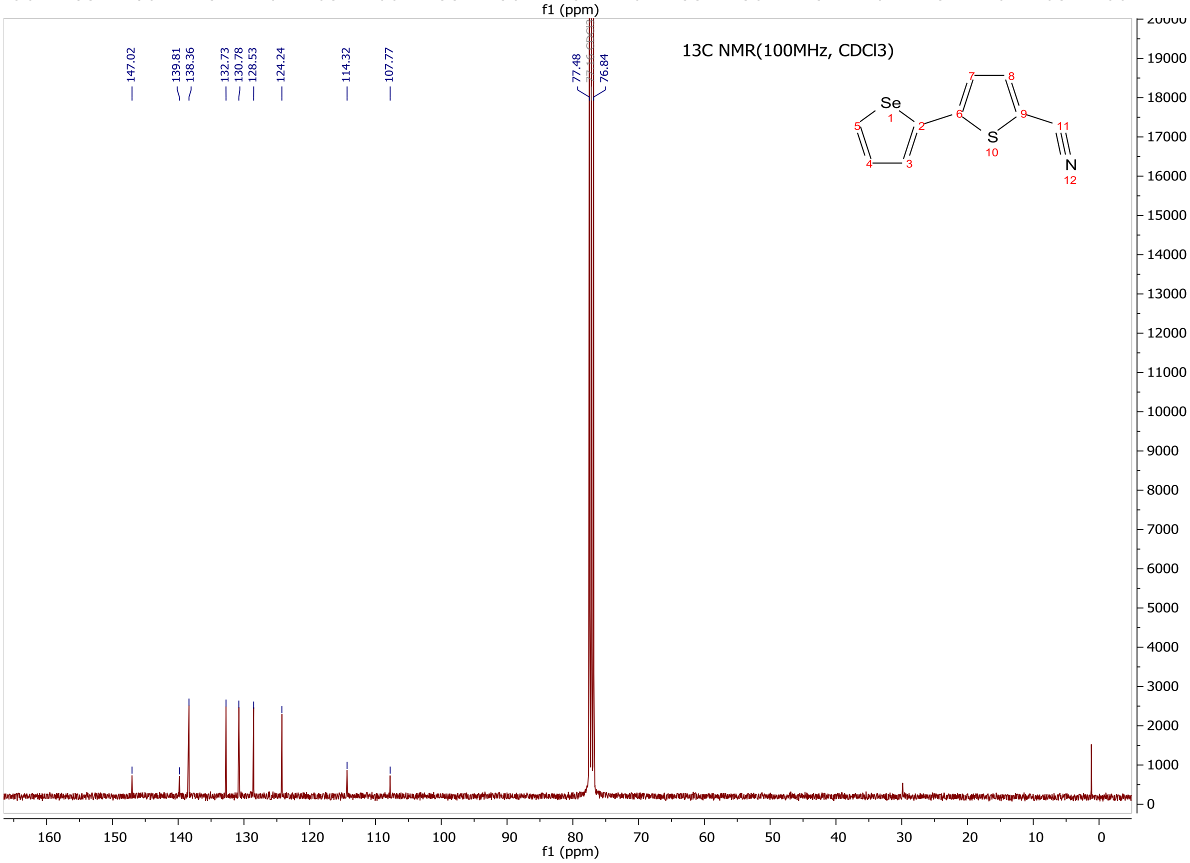

2-Chloro-5-(selenophen-2-yl)thiophene (4)

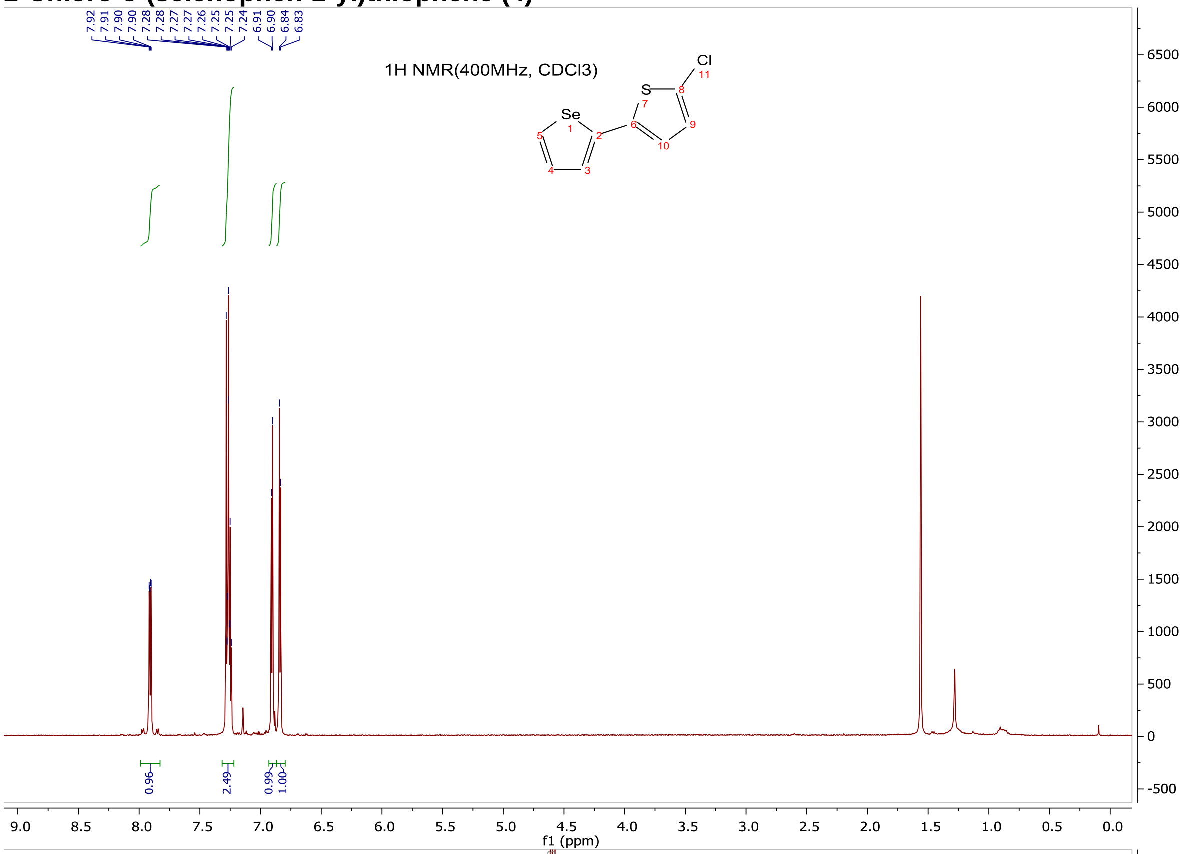

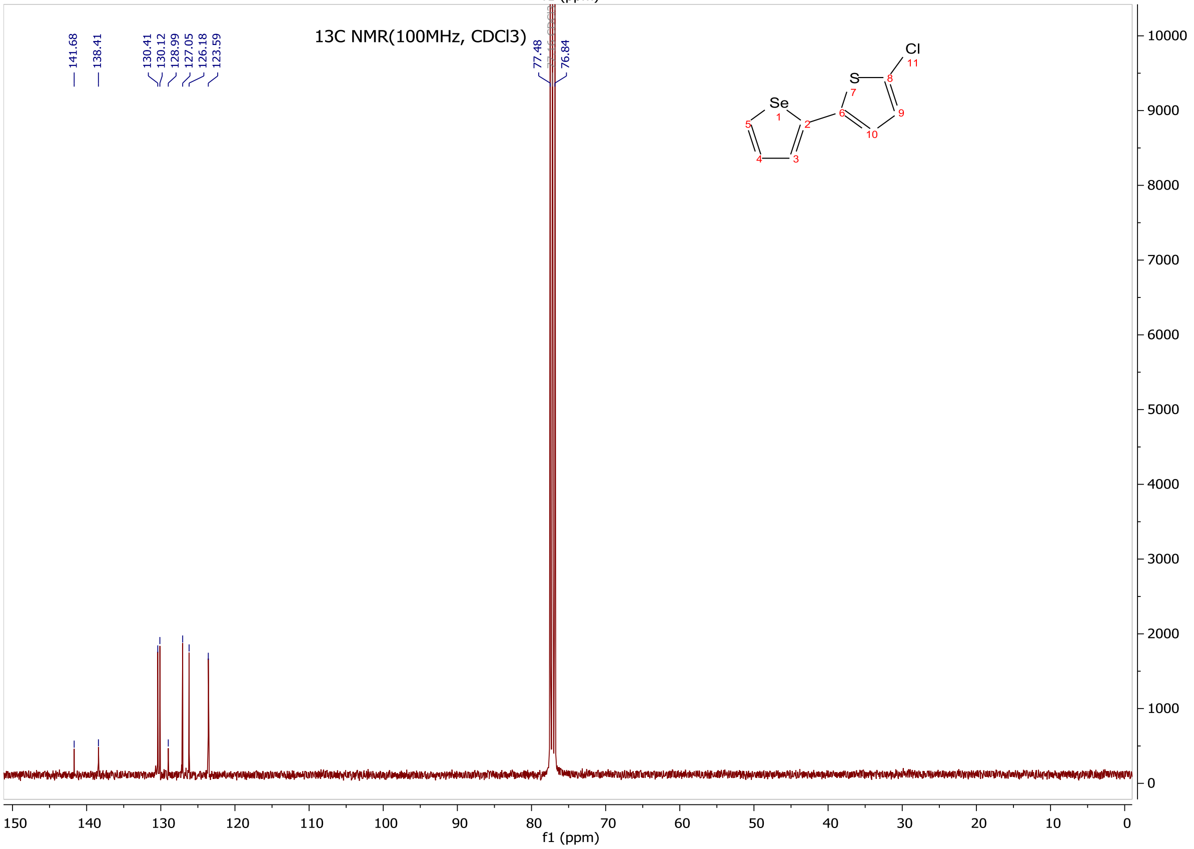

2-Pentyl-5-(selenophen-2-yl)thiophene (5)

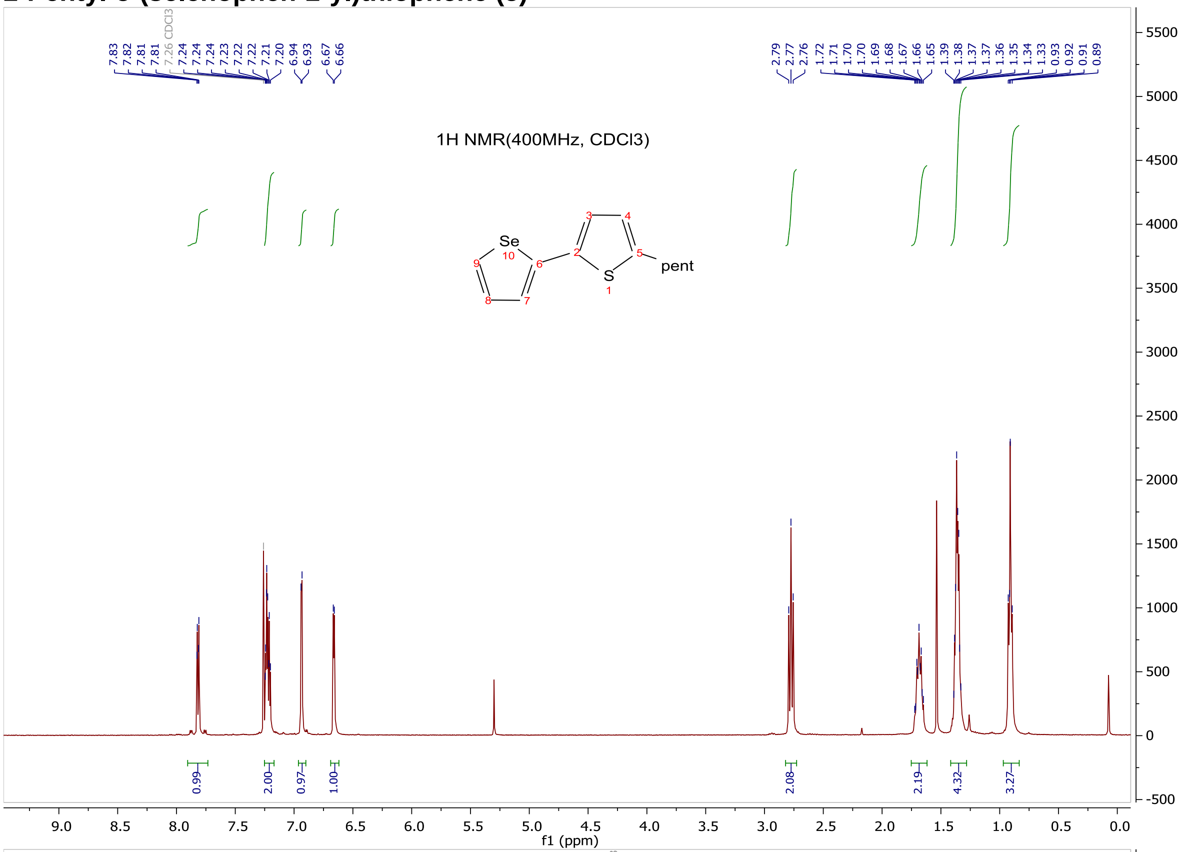

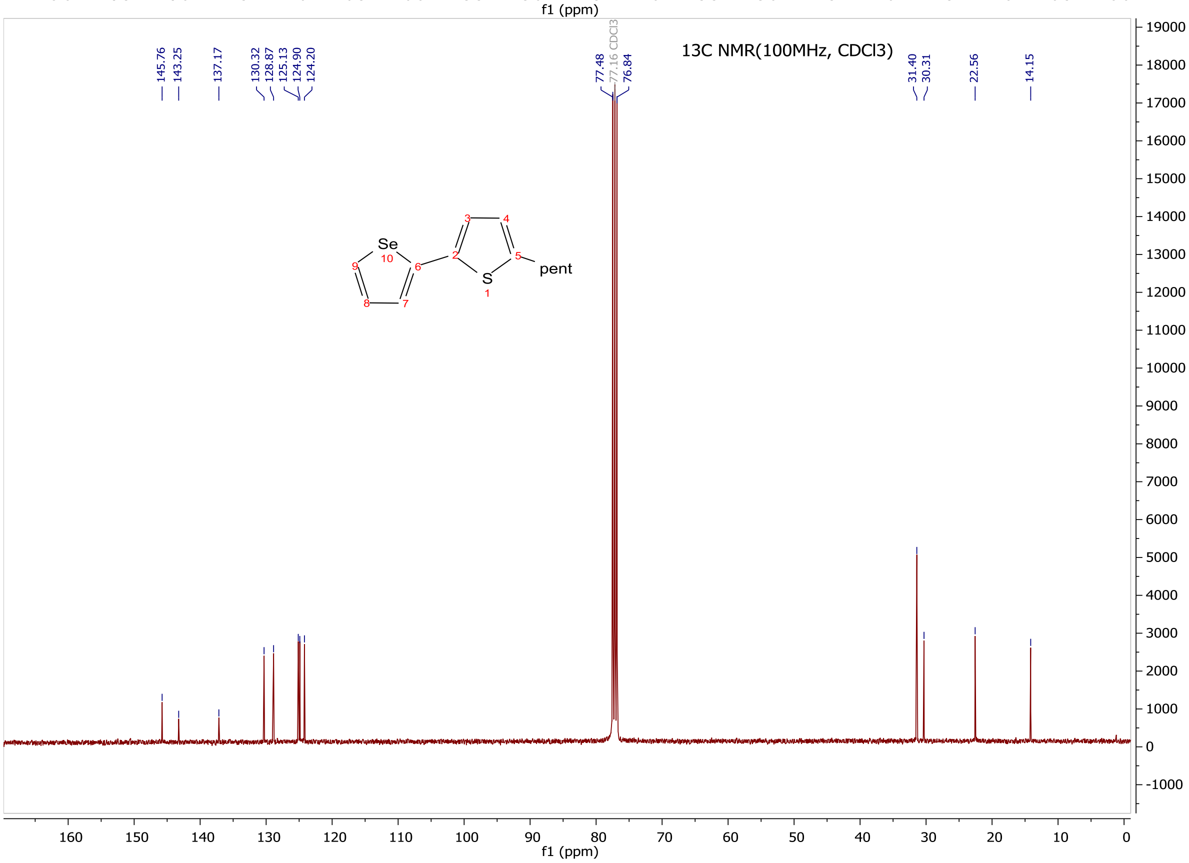

1-Phenyl-2-(selenophen-2-yl)-pyrrole (6)

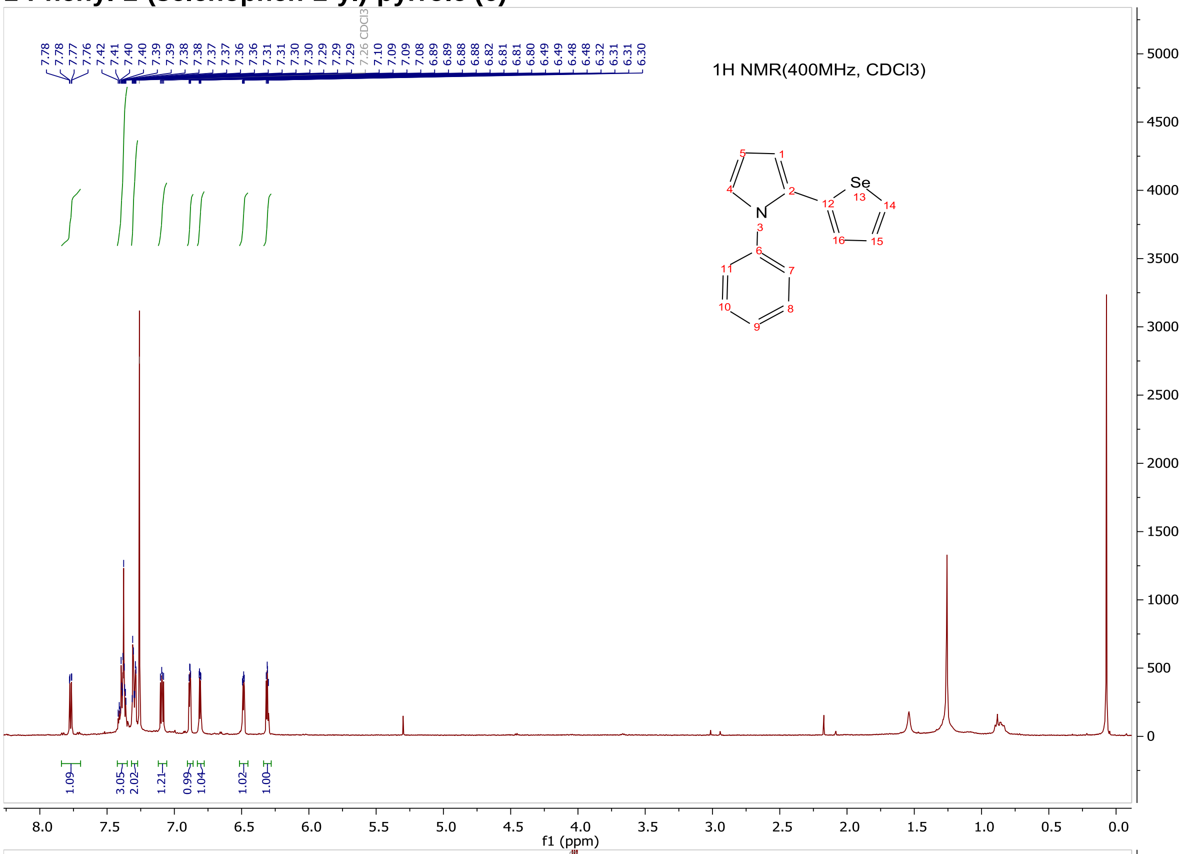

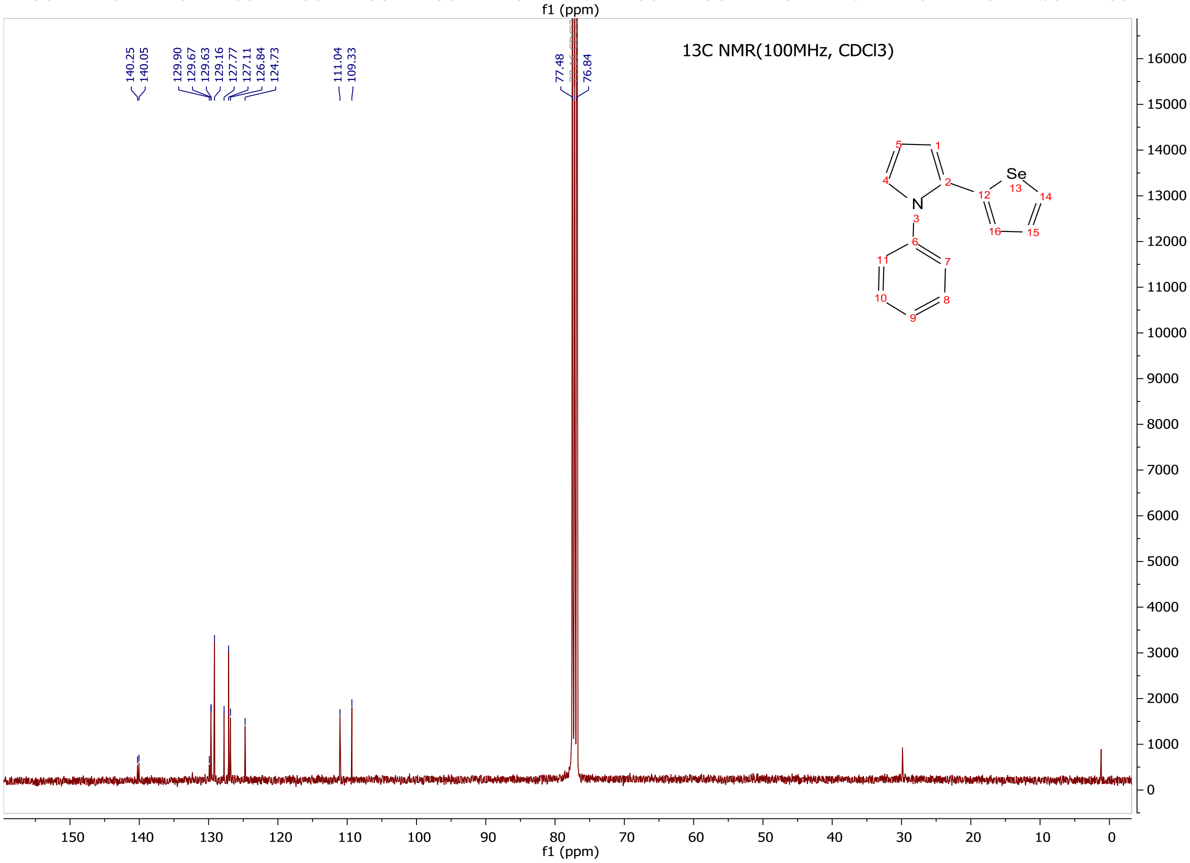

3-(Selenophen-2-yl)imidazo[1,2-*a*]pyridine (7)

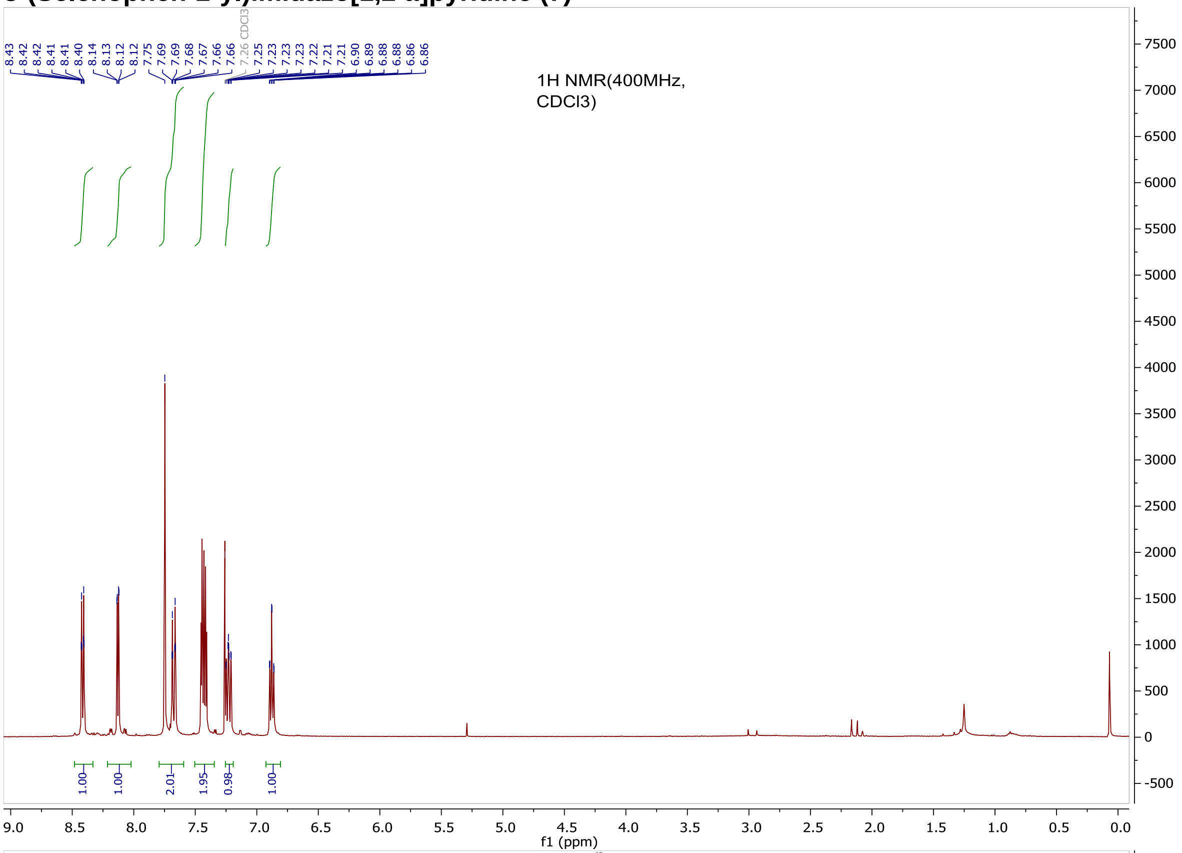

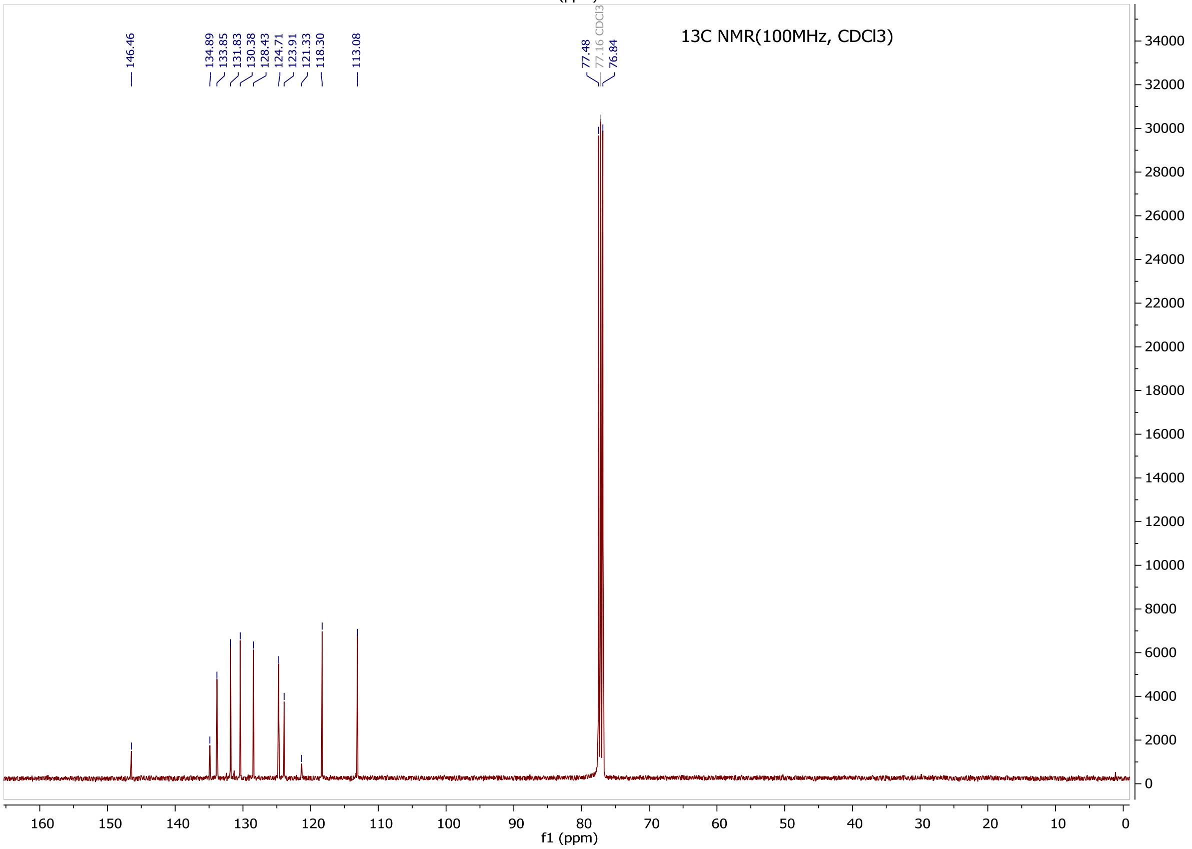

2,5-Bis(2,4-dimethylthiazol-5-yl)selenophene (8)

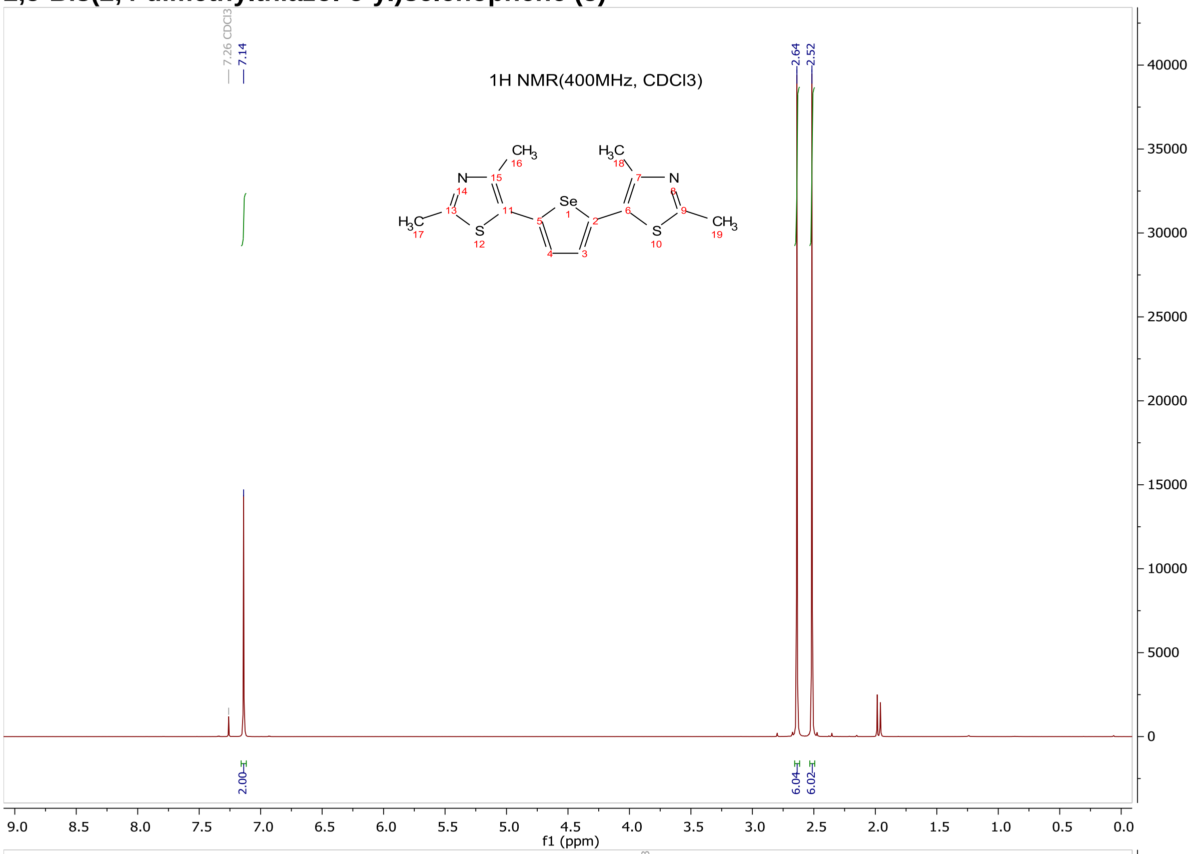

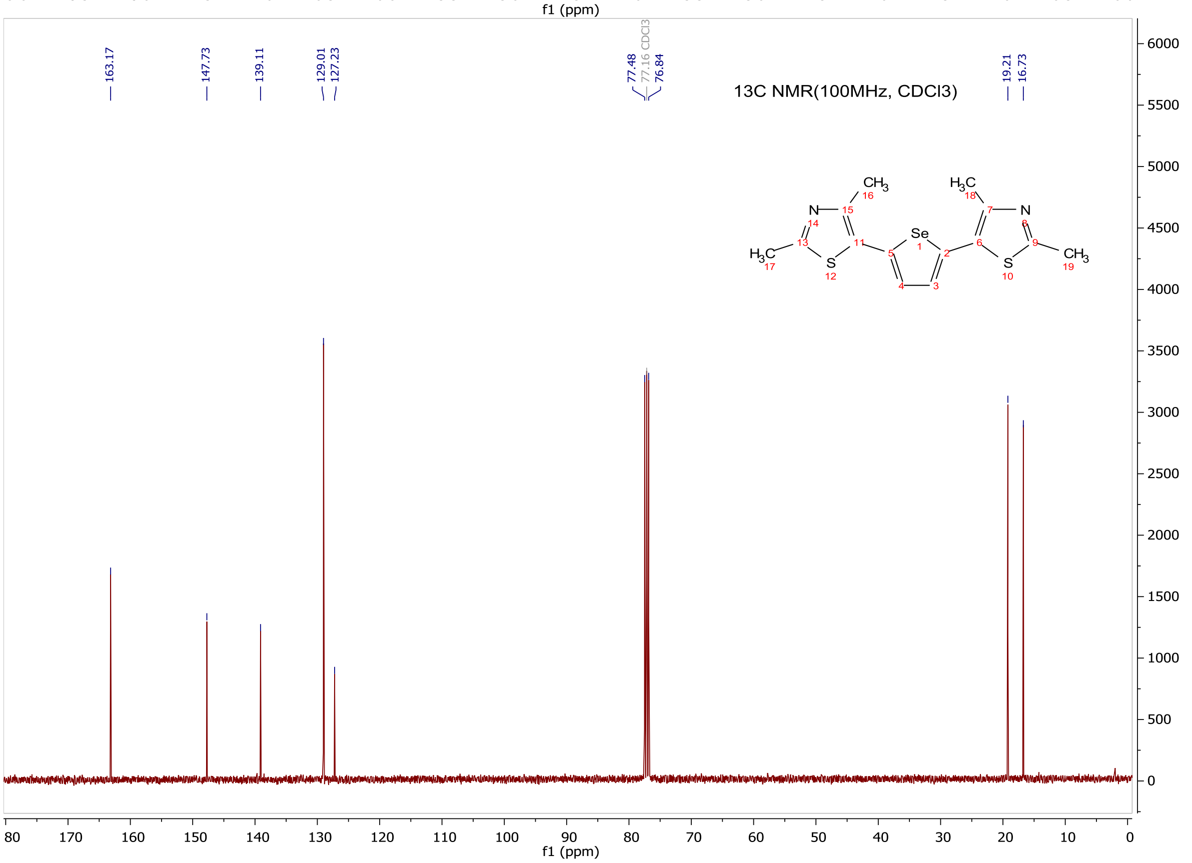

2,5-Bis(2-isopropyl-4-methylthiazol-5-yl)selenophene (9)

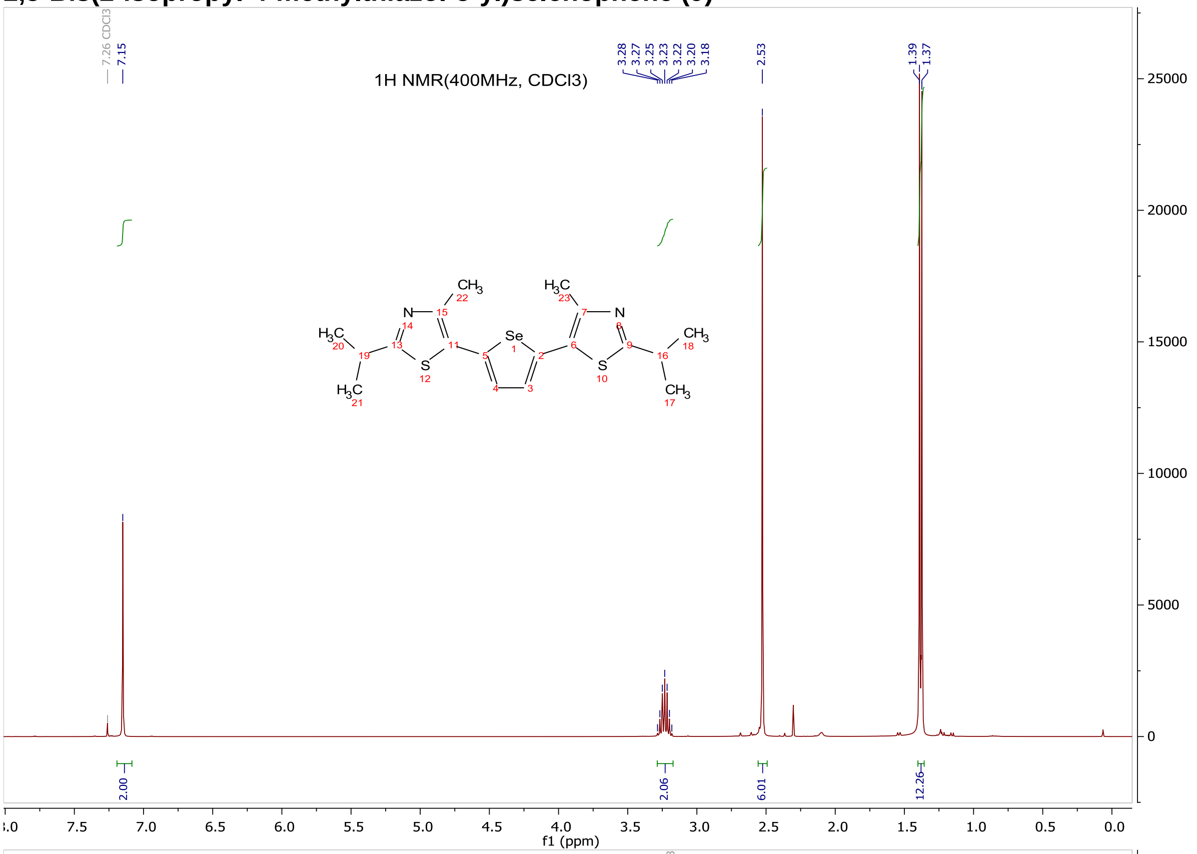

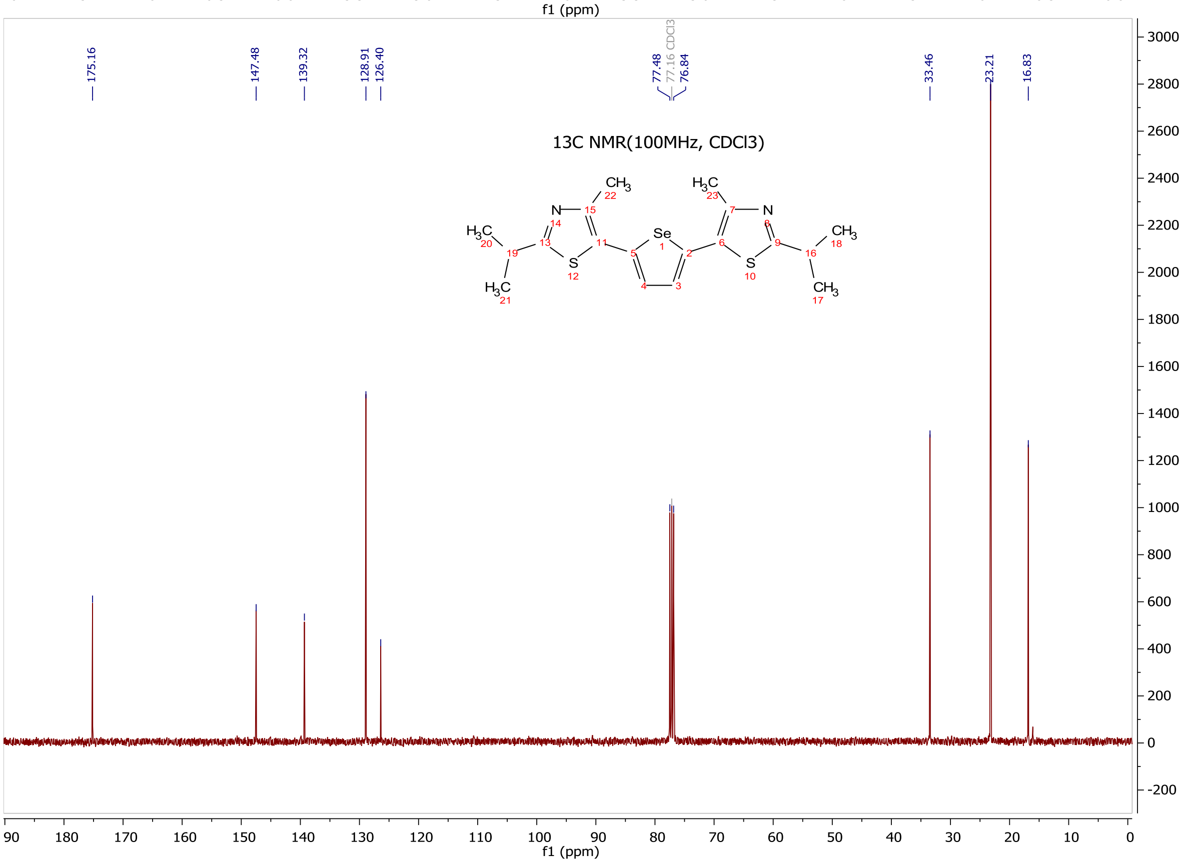

2,5-Bis(5-pentylthiophen-2-yl)selenophene (10)

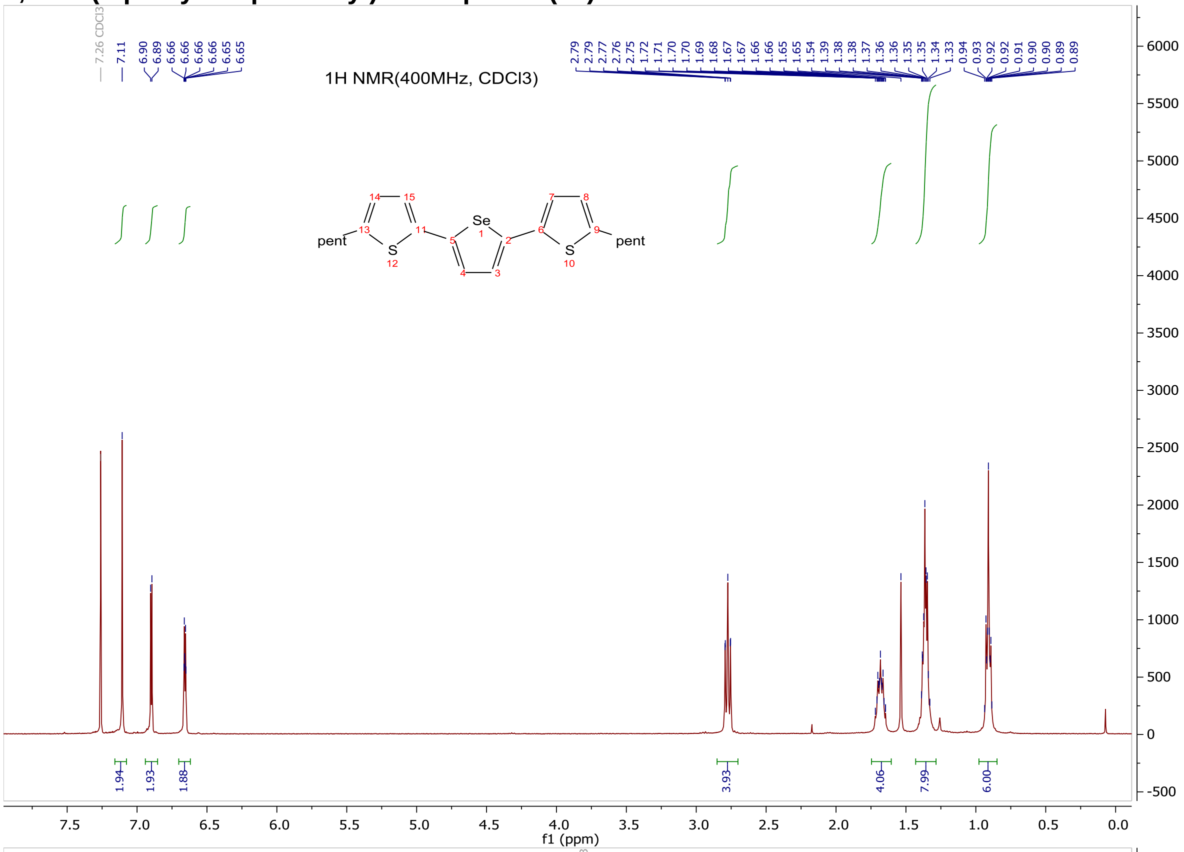

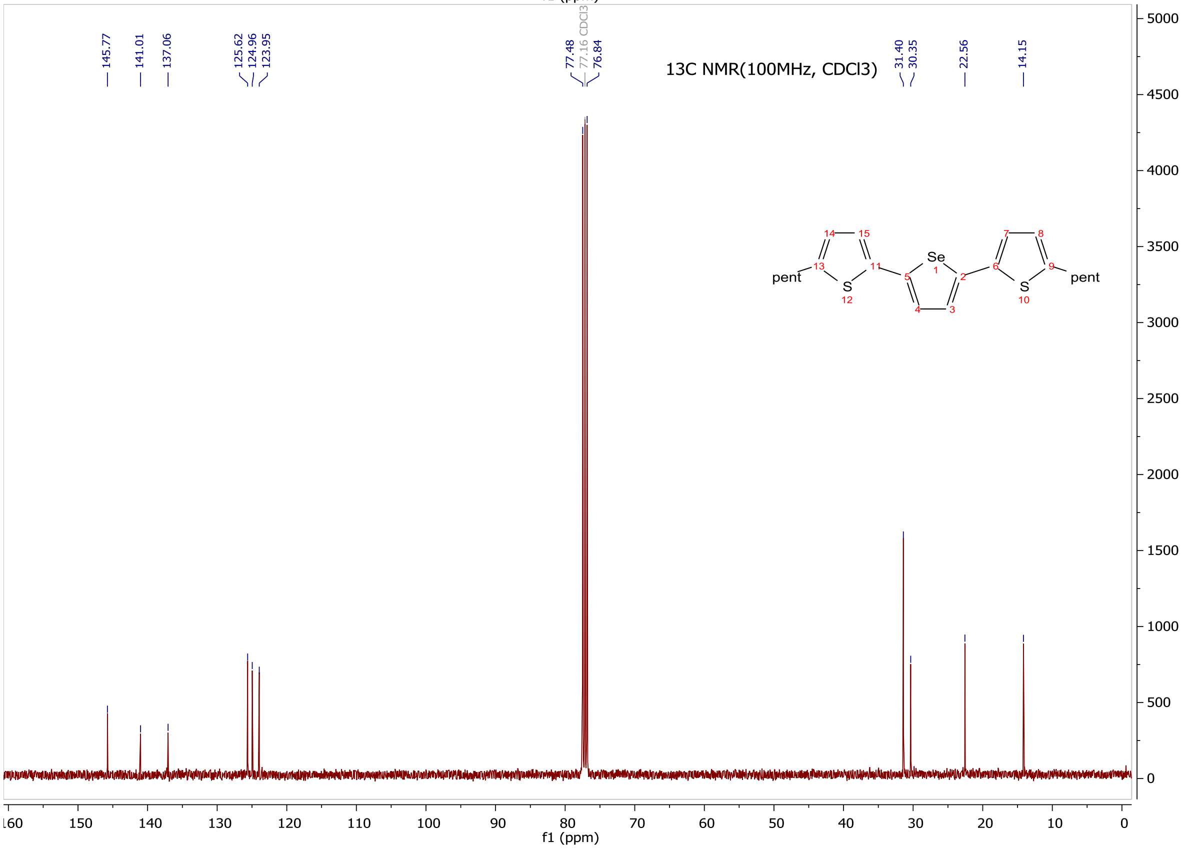

2,5-Bis(5-chlorothiophen-2-yl)selenophene (11)

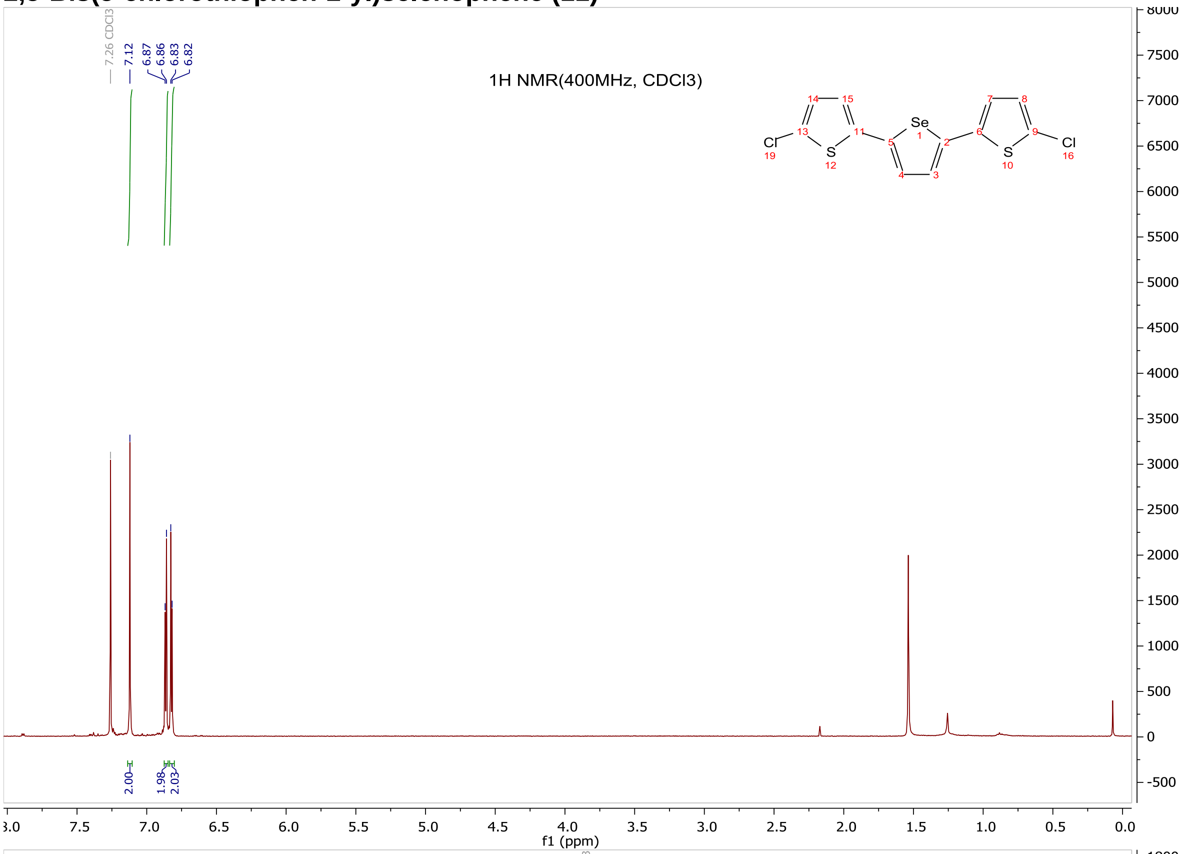

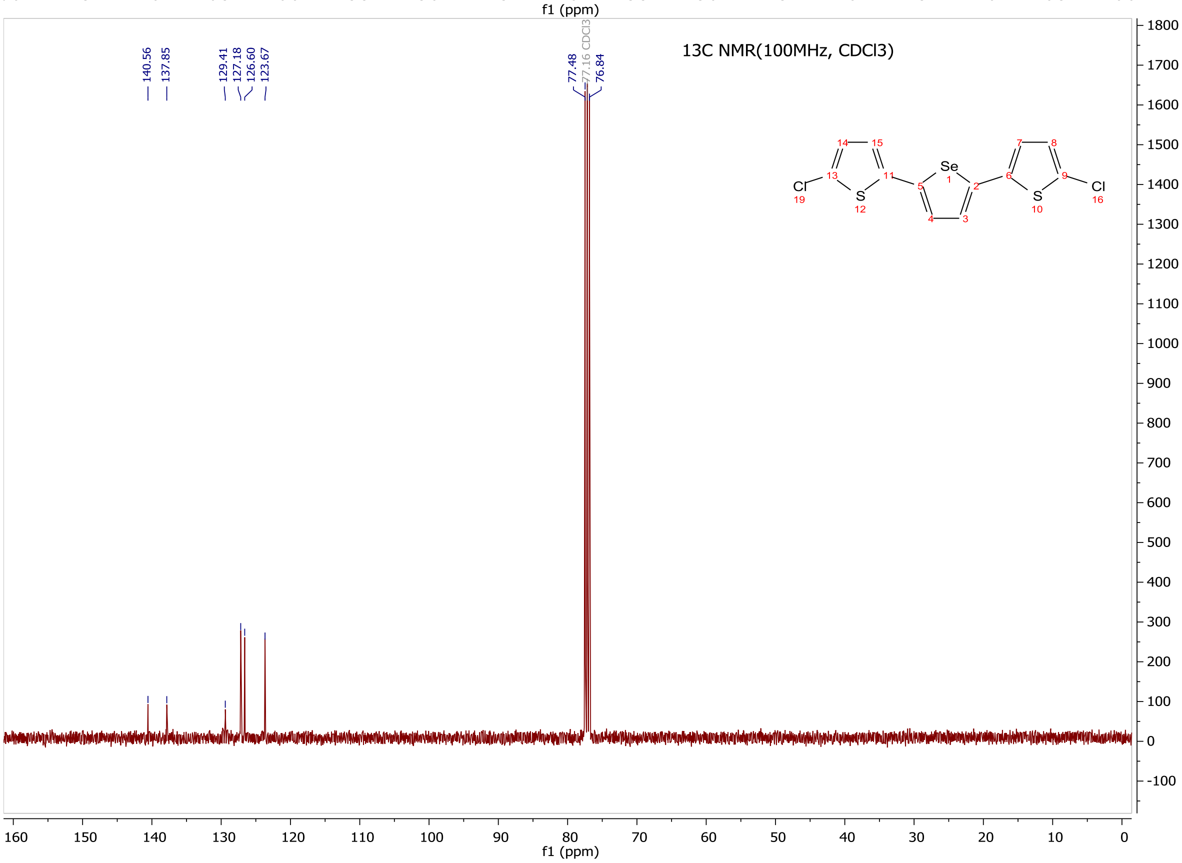

2,5-Bis(3-methylthiophen-2-yl)selenophene (12)

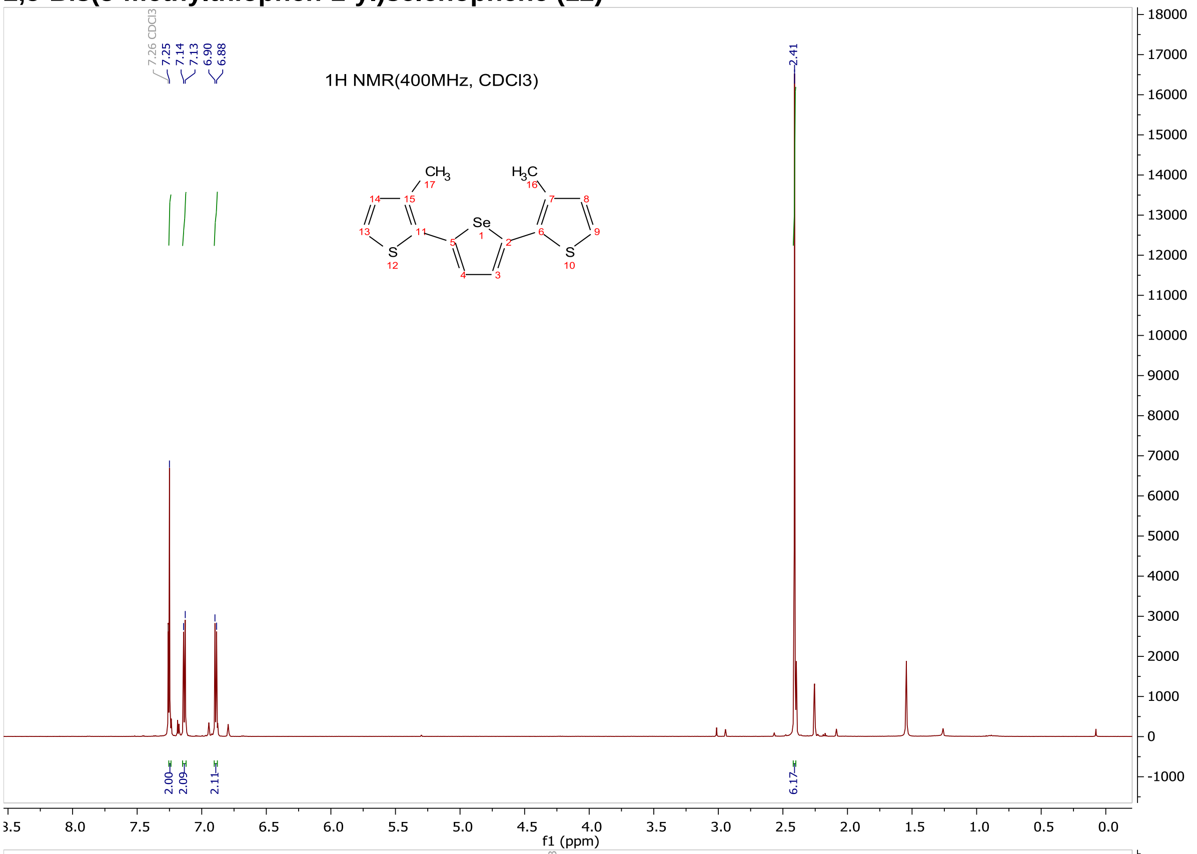

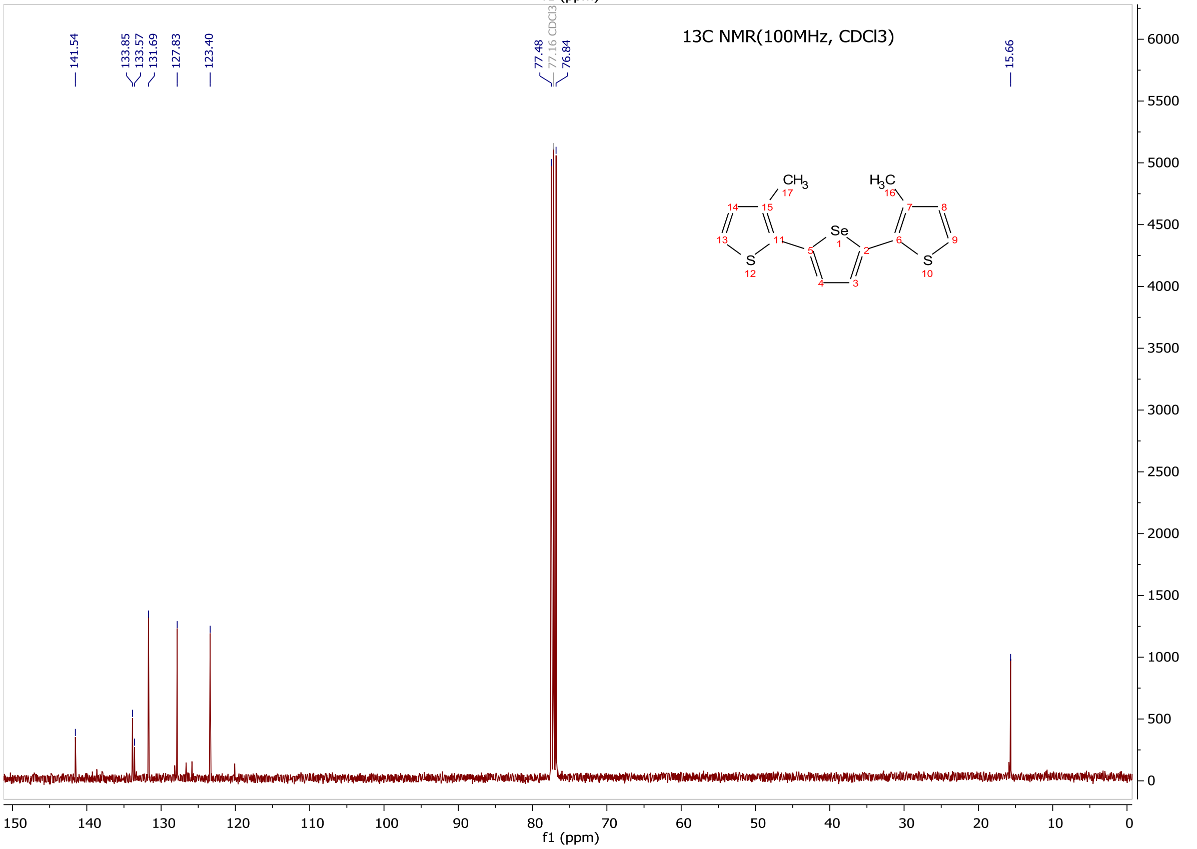

2,5-Bis(3-chlorothiophen-2-yl)selenophene (13)

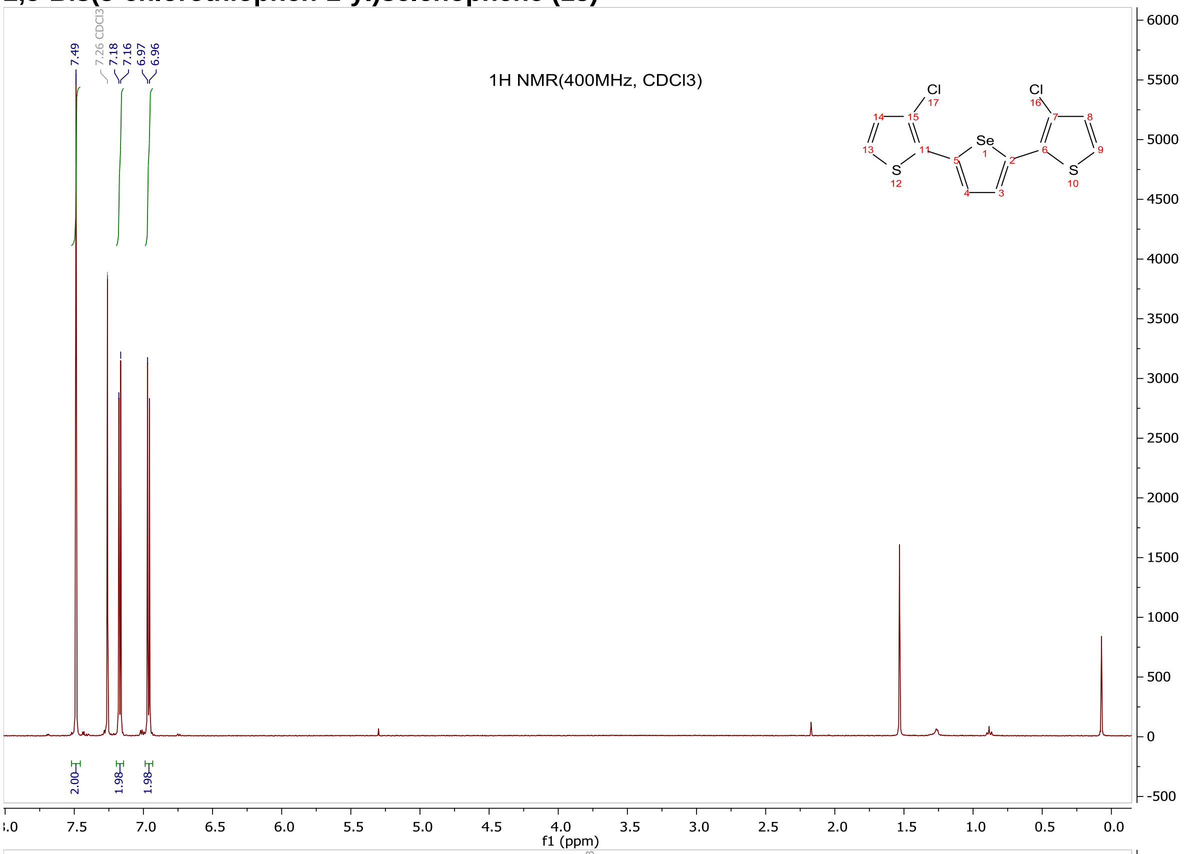

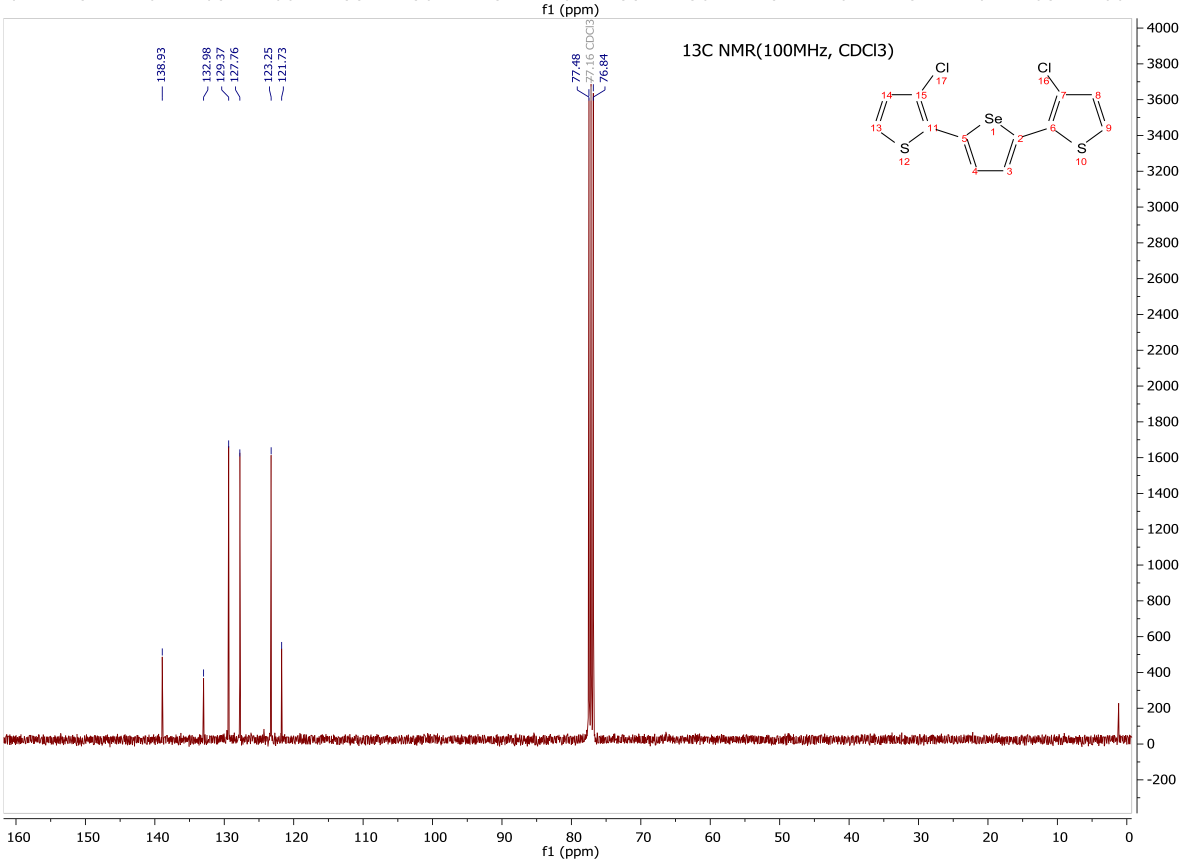

2,5-Bis(1-methylpyrrol-2-yl)selenophene (14)

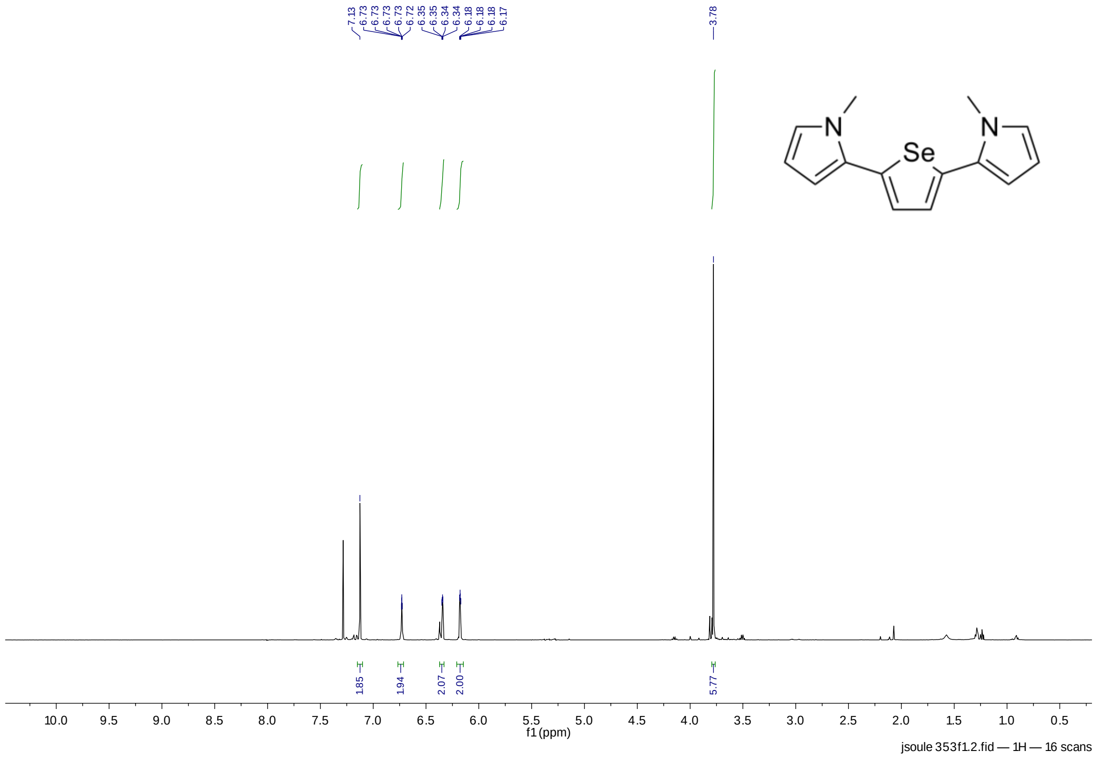

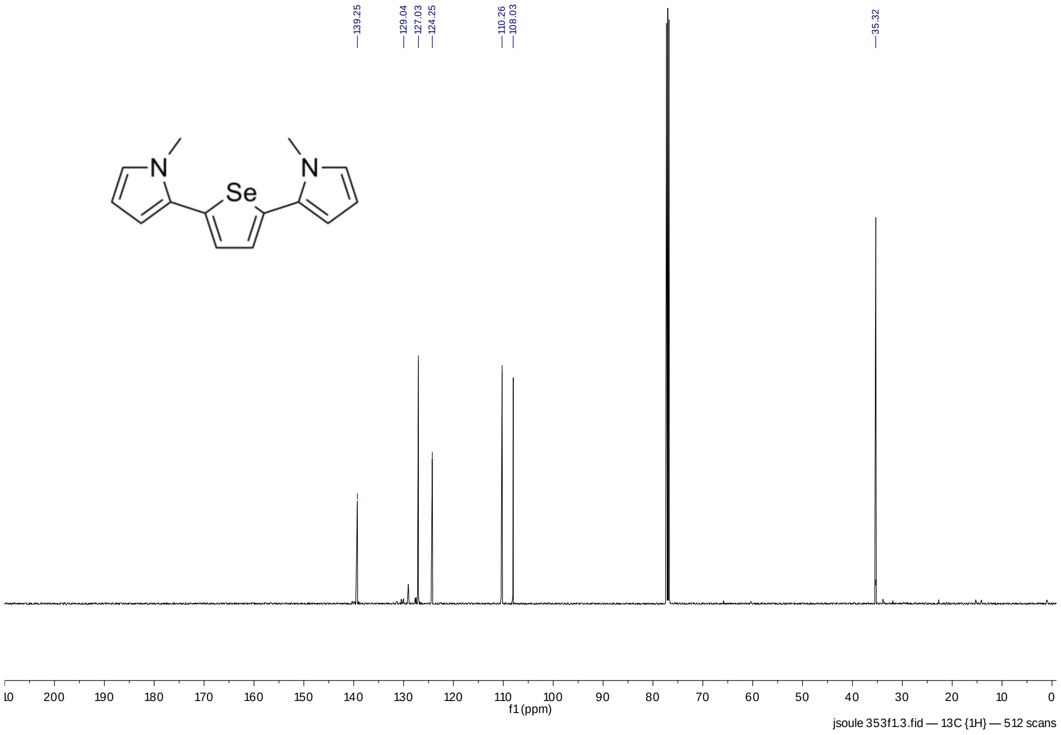

4-(5-Bromoselenophen-2-yl)benzonitrile (15)

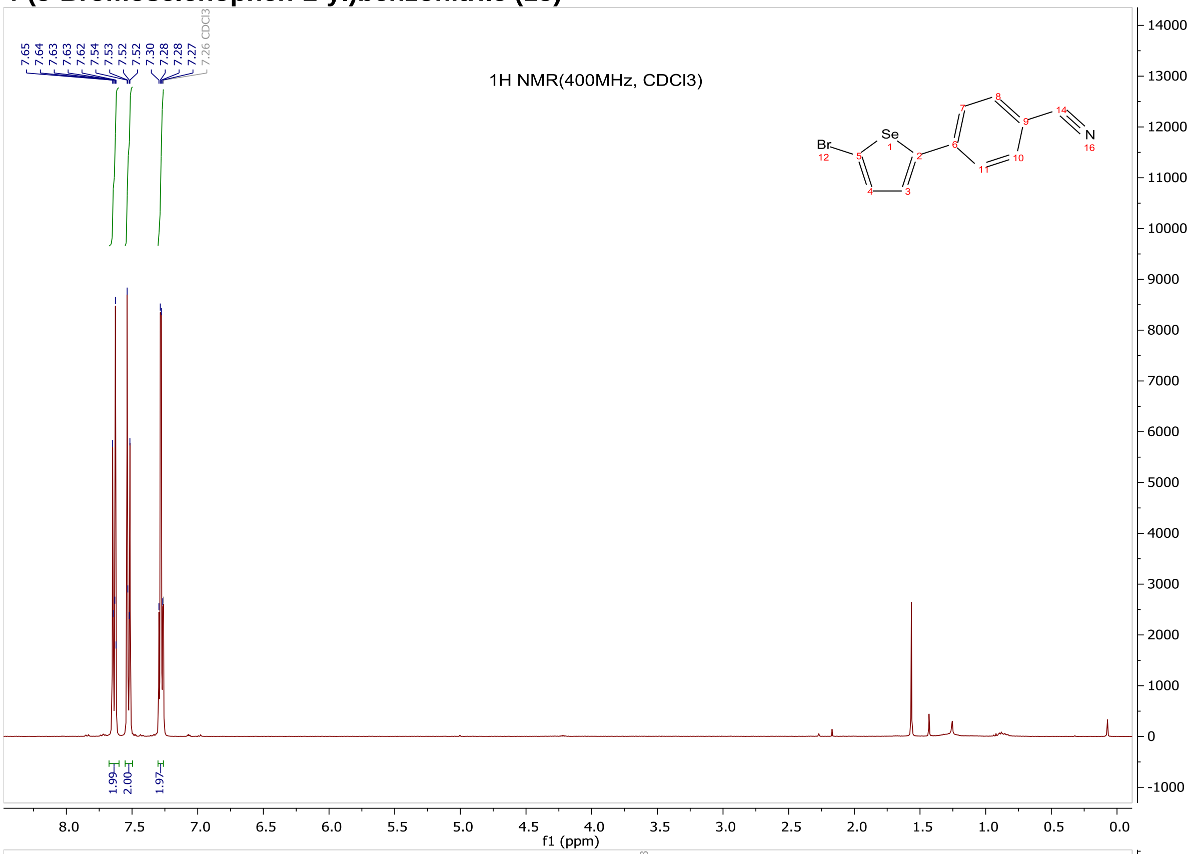

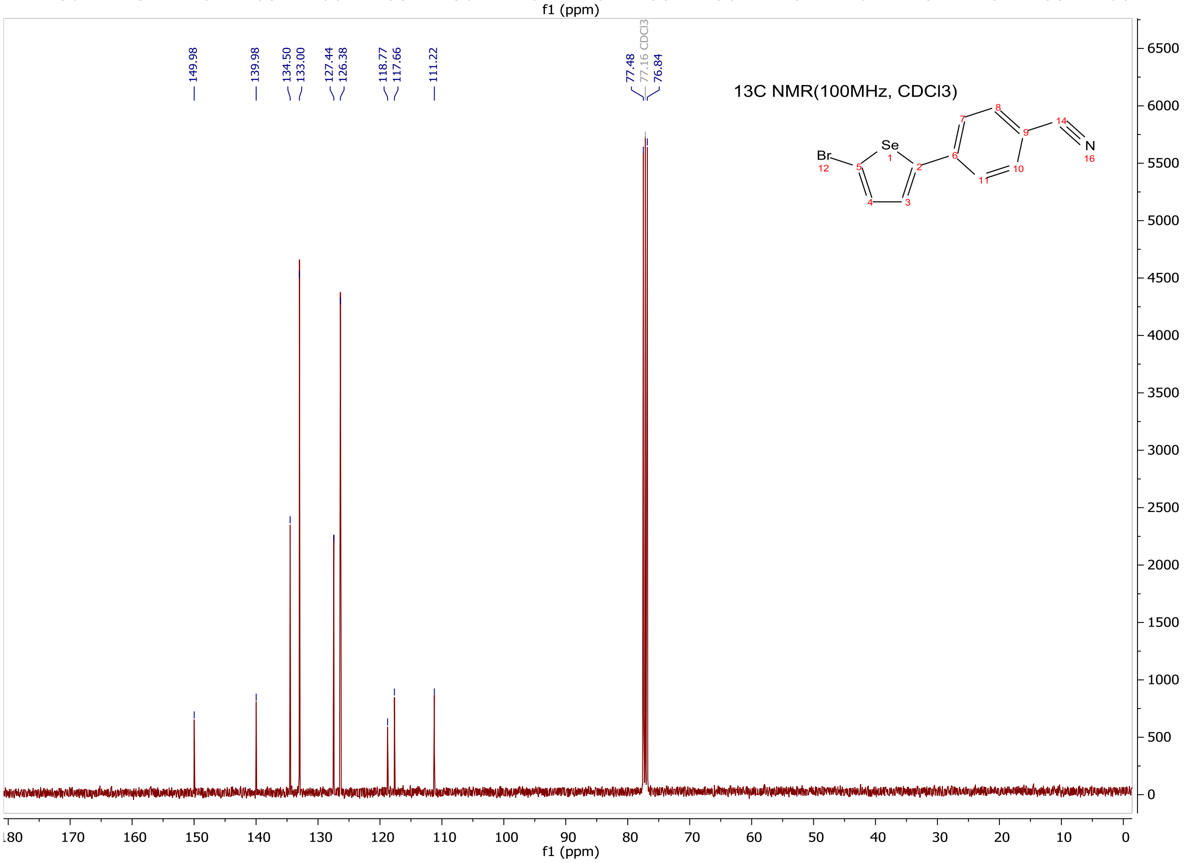

2-Bromo-5-(4-chlorophenyl)selenophene (16)

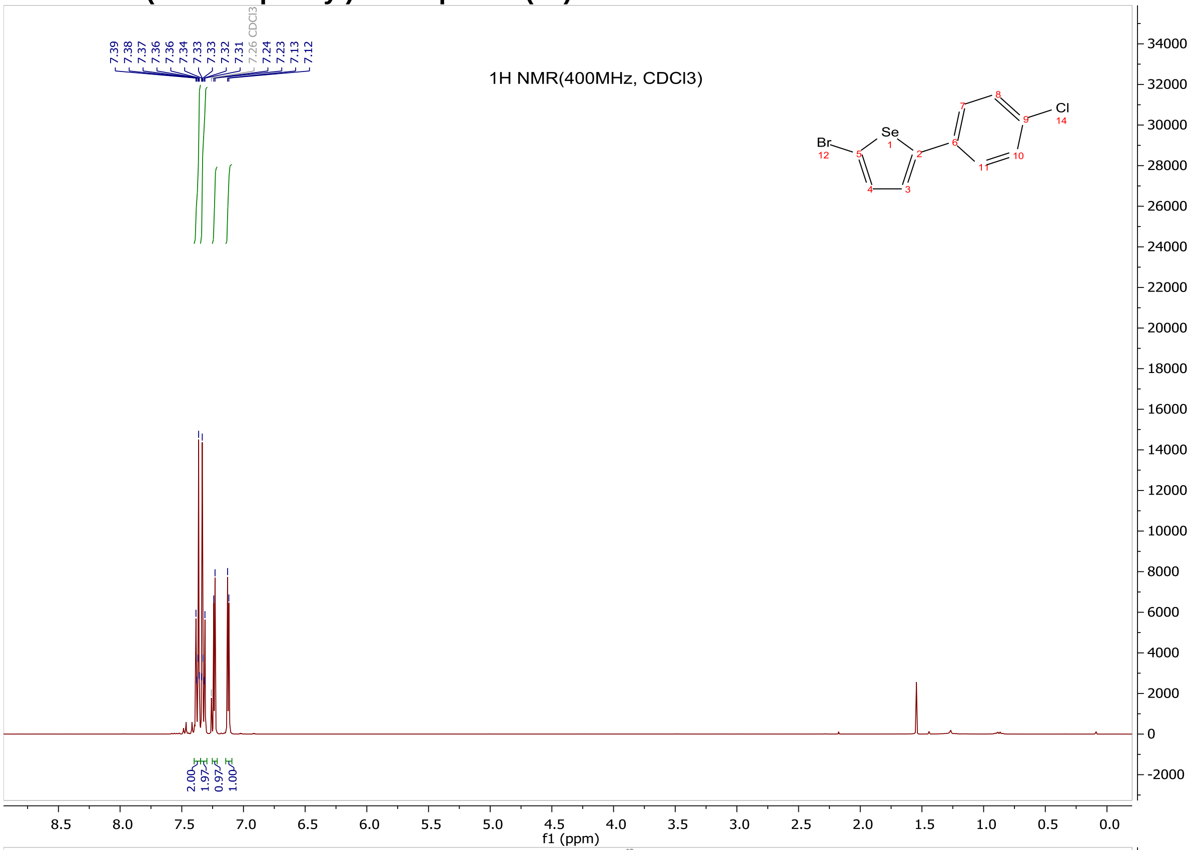

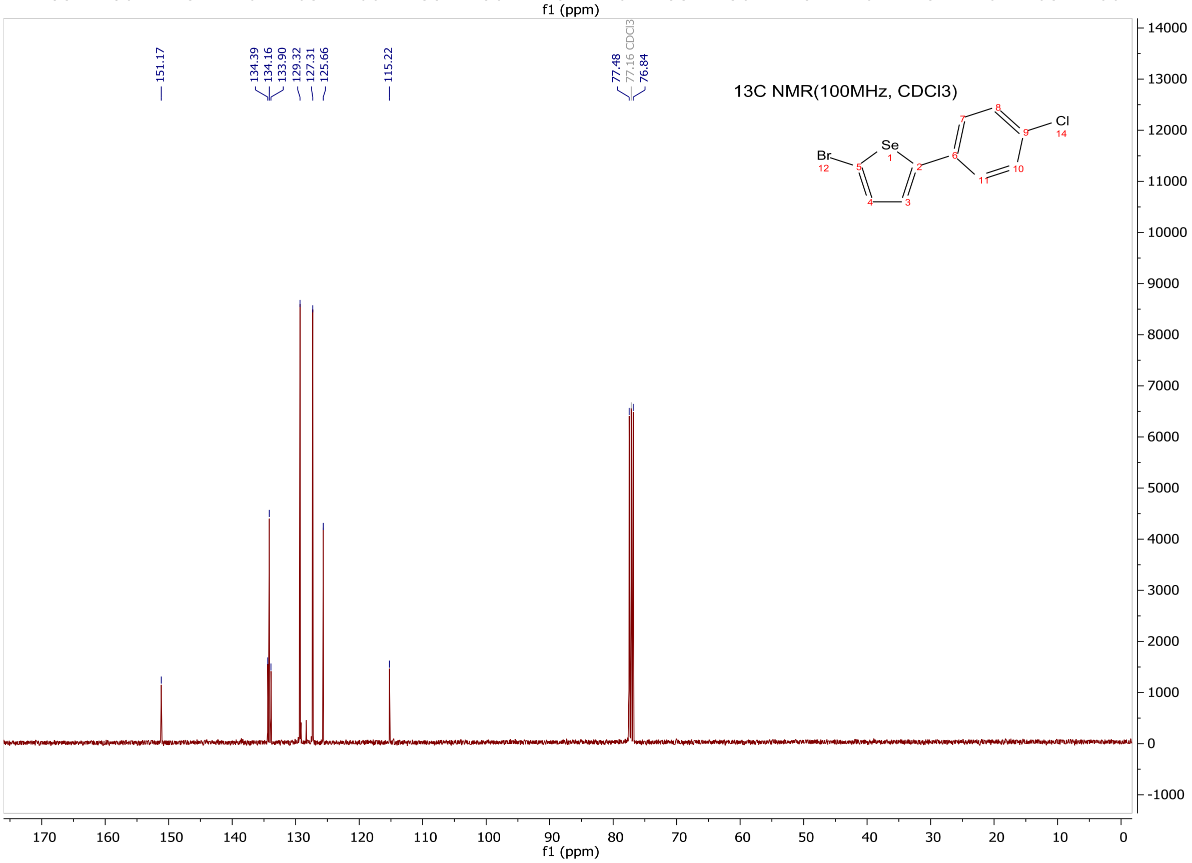

1-(4-(5-Bromoselenophen-2-yl)phenyl)ethan-1-one (17)

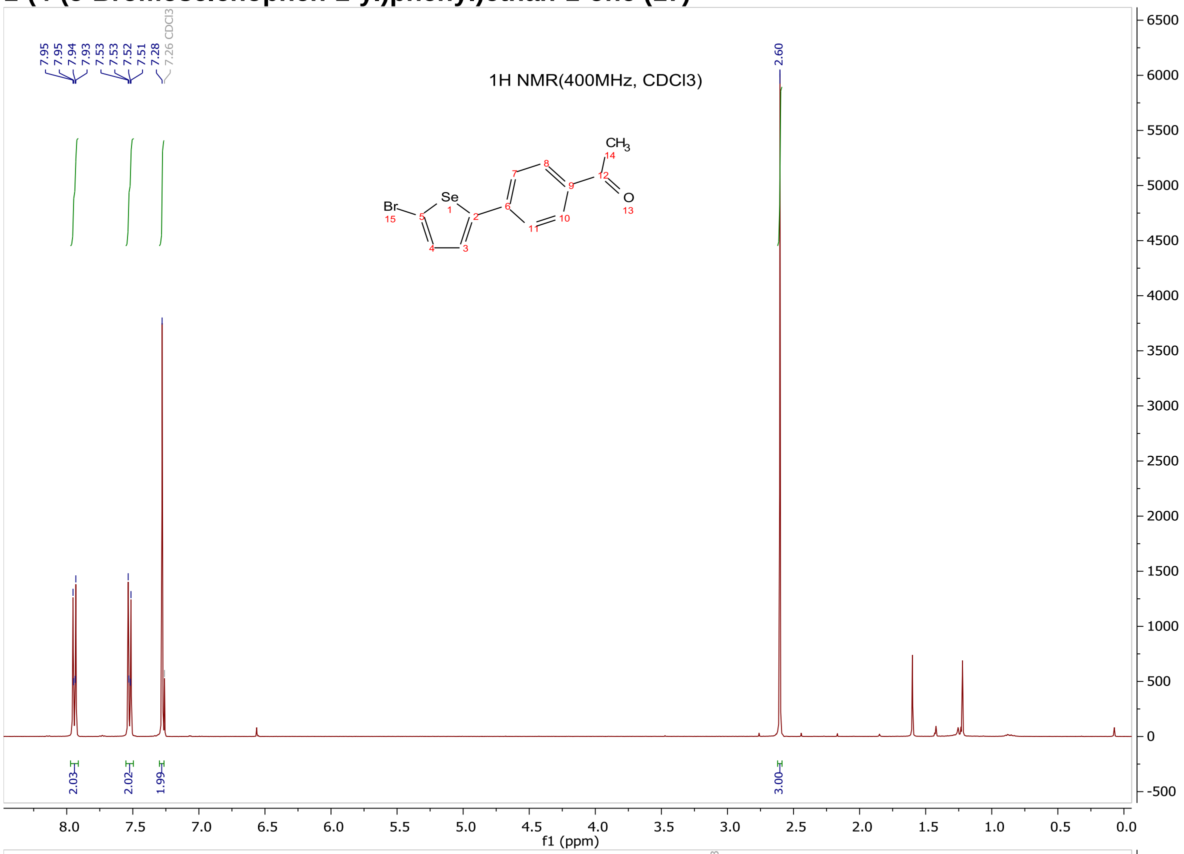

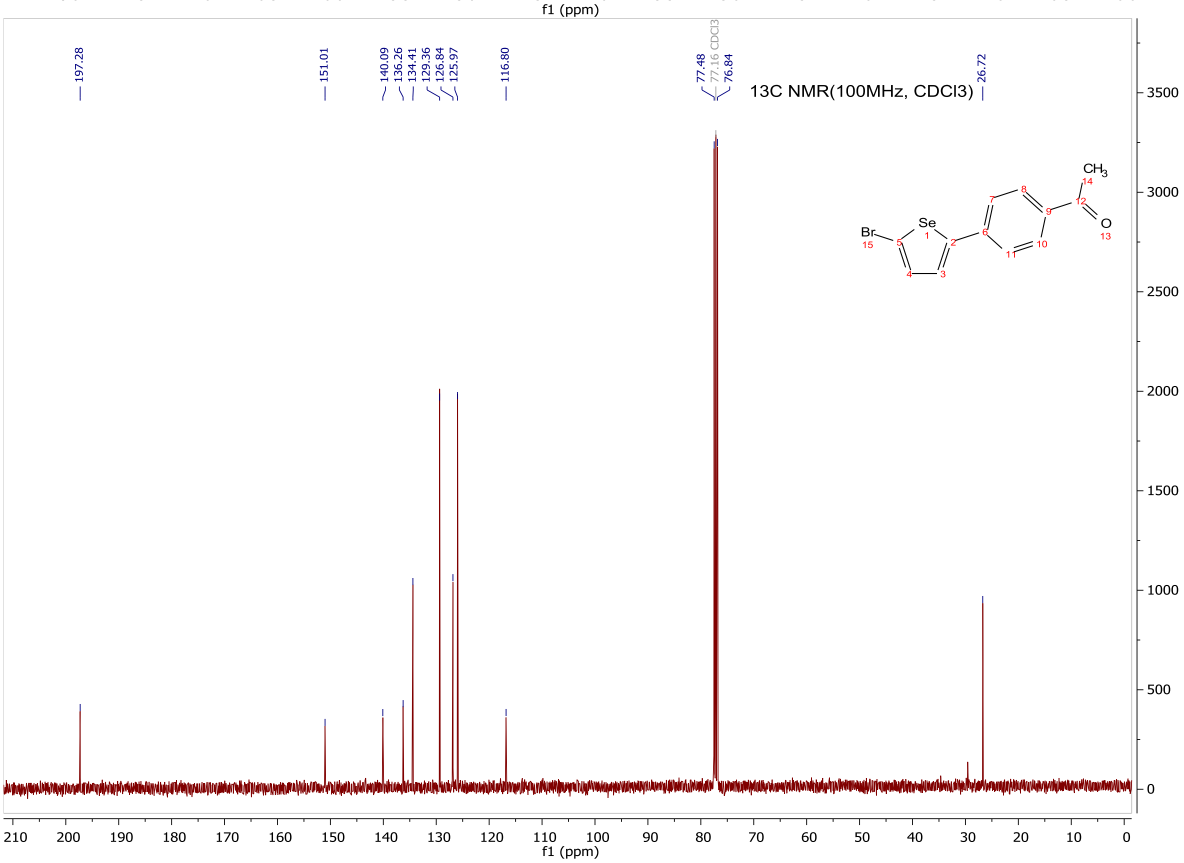

4-(5-(2-Ethyl-4-methylthiazol-5-yl)selenophen-2-yl)benzonitrile (18)

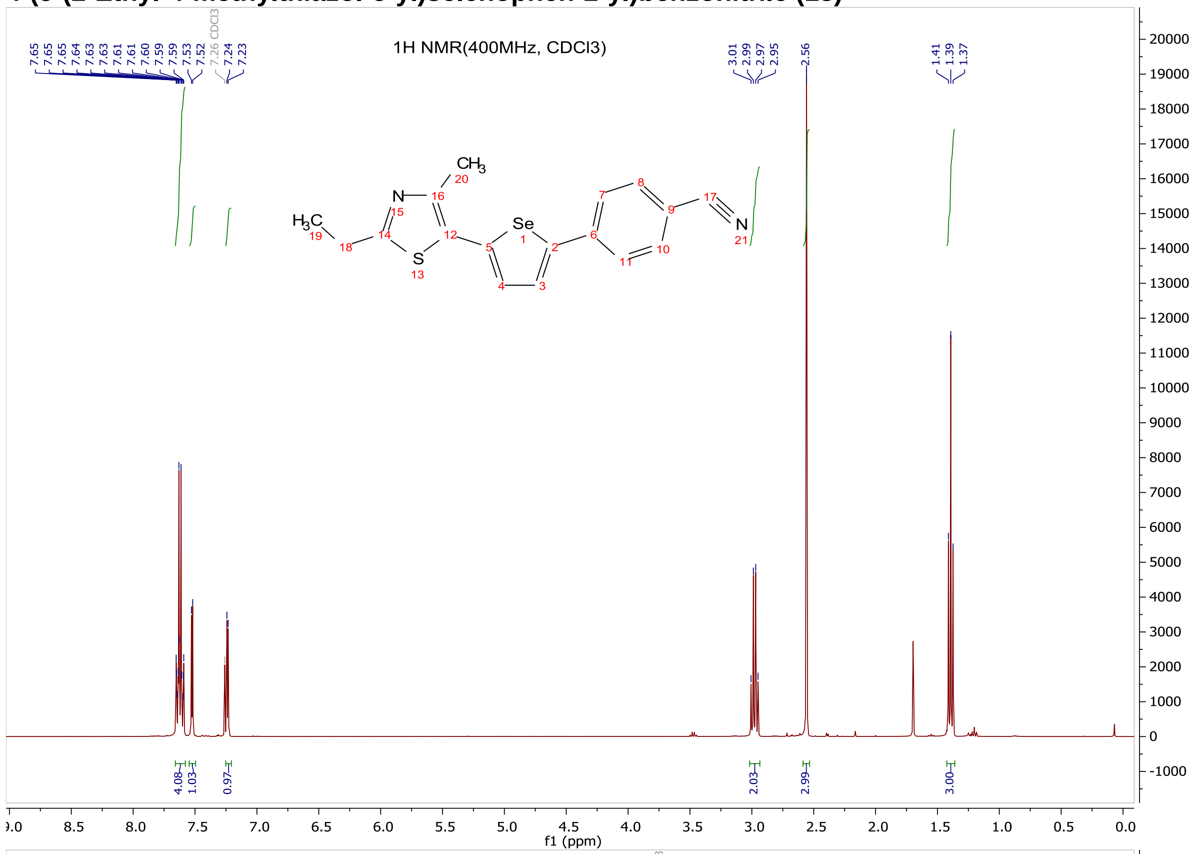

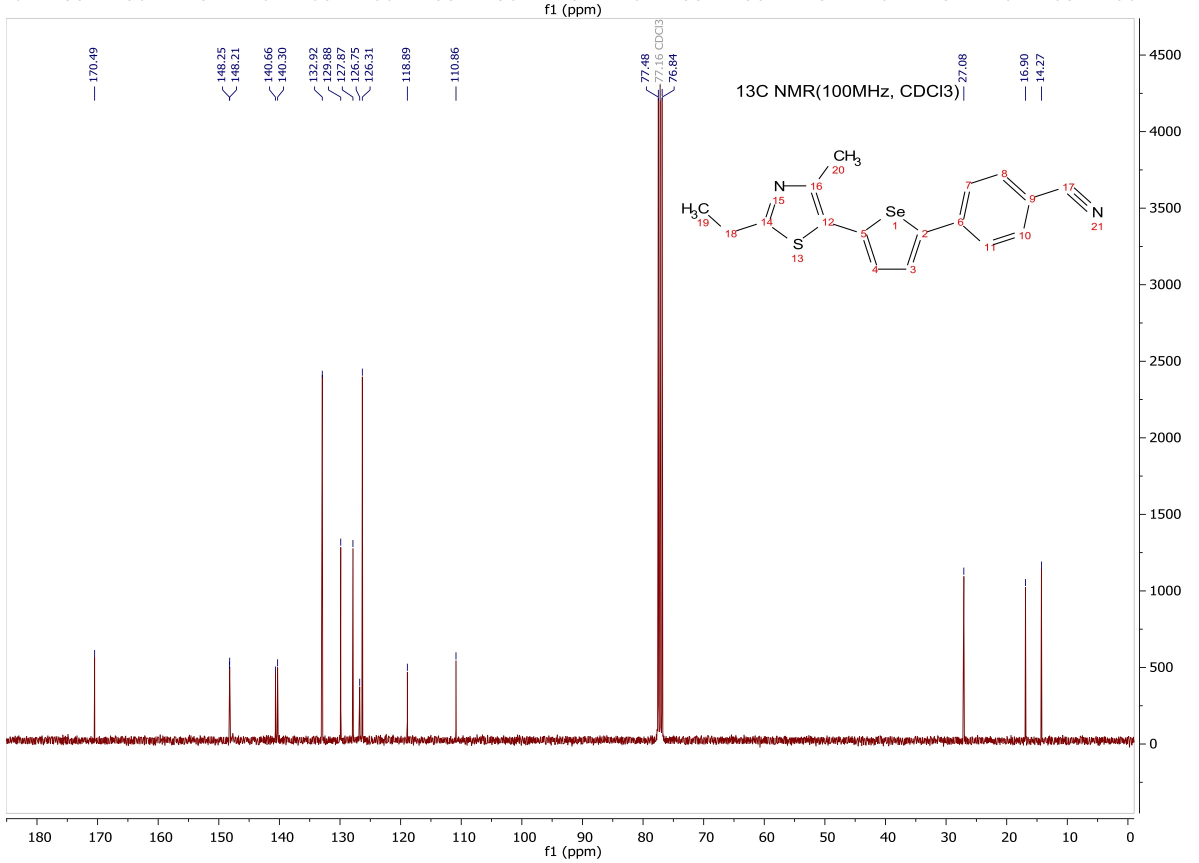

5-(5-(4-Chlorophenyl)selenophen-2-yl)-2-ethyl-4-methylthiazole (19)

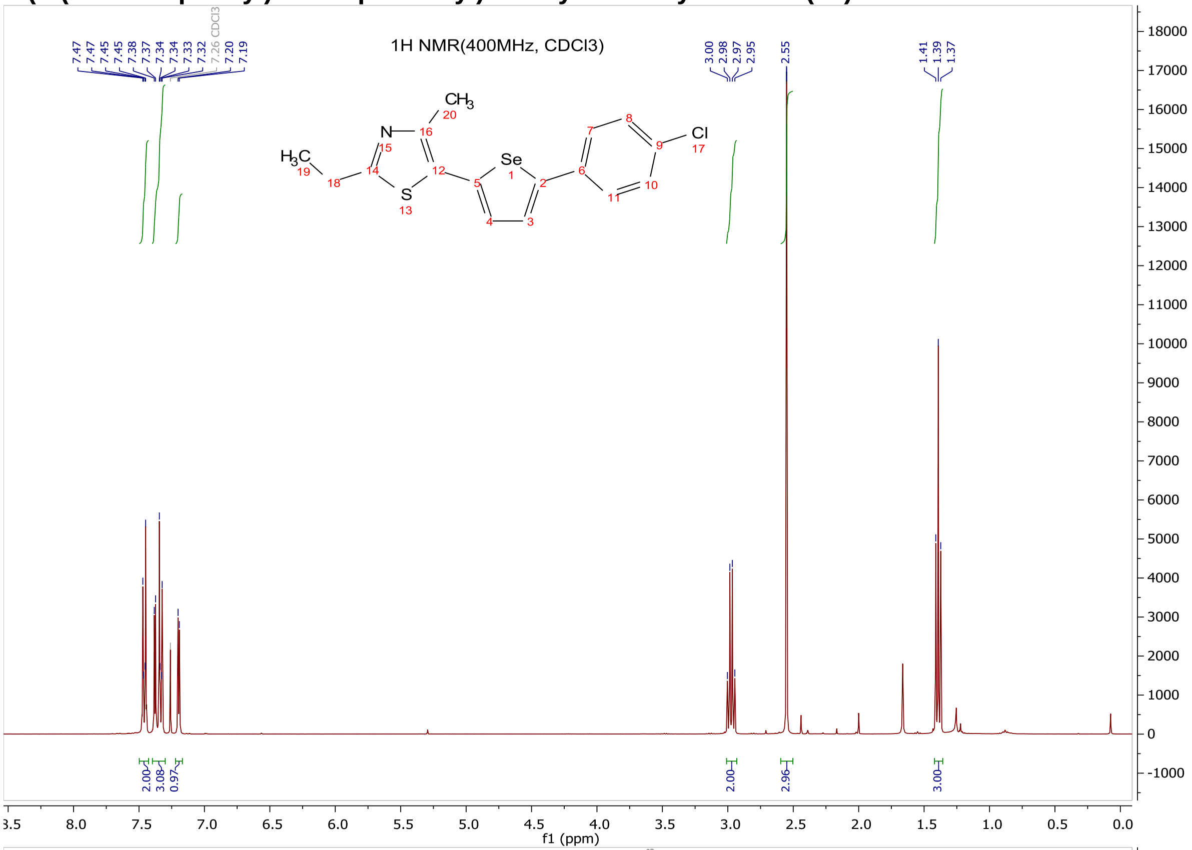

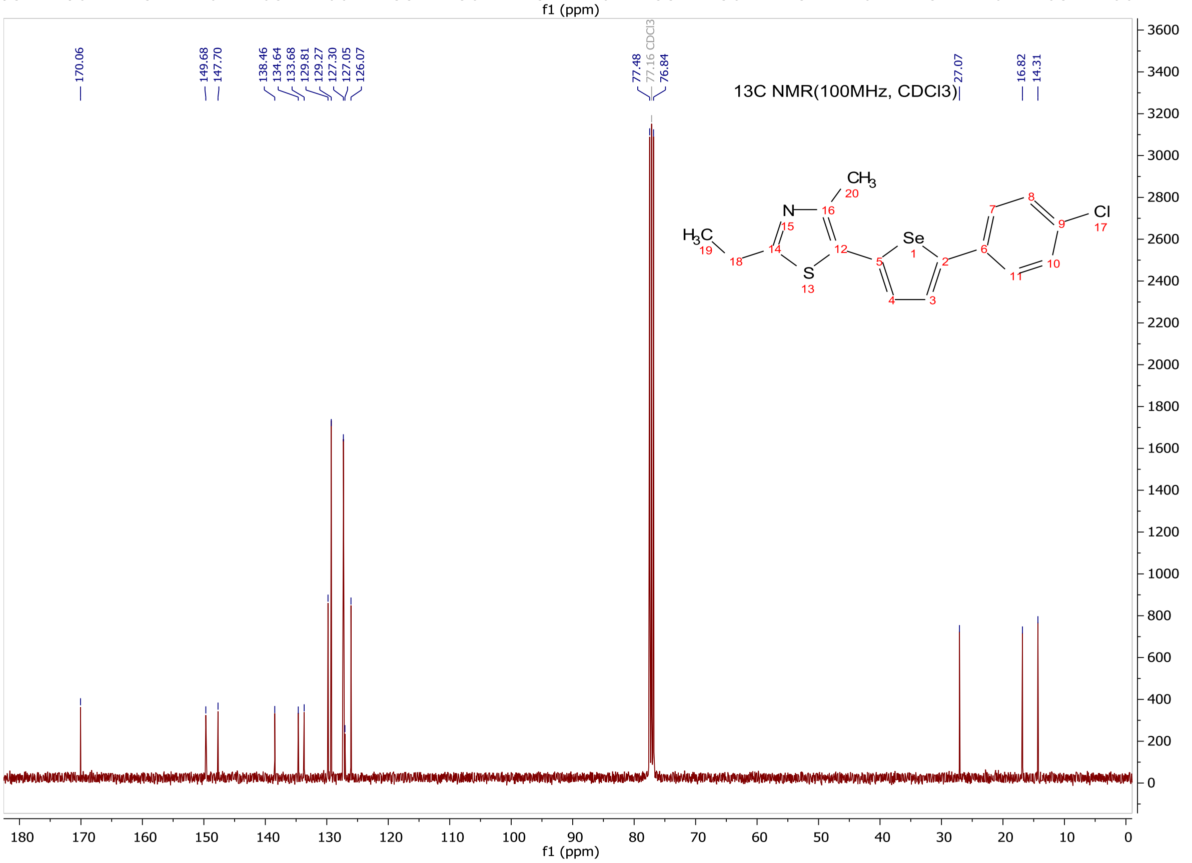

1-(4-(5-(2-Ethyl-4-methylthiazol-5-yl)selenophen-2-yl)phenyl)ethan-1-one (20)

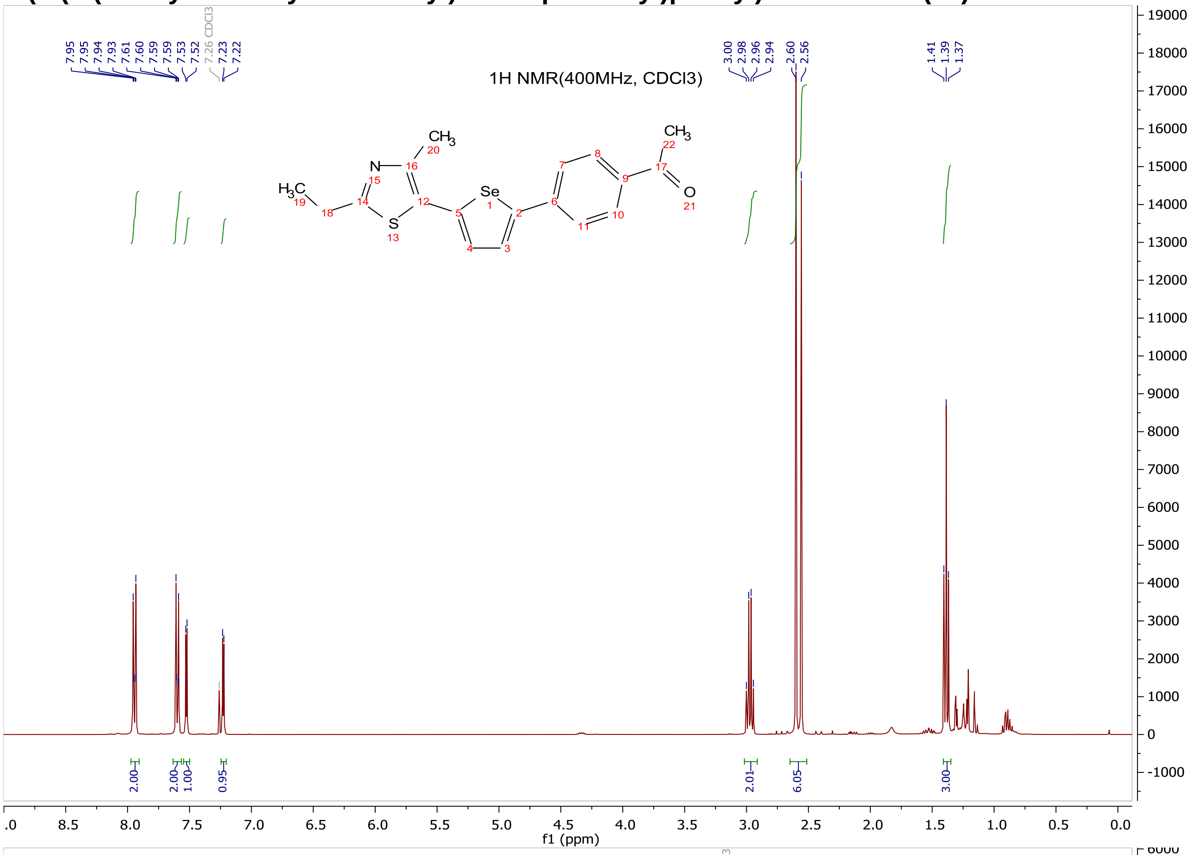

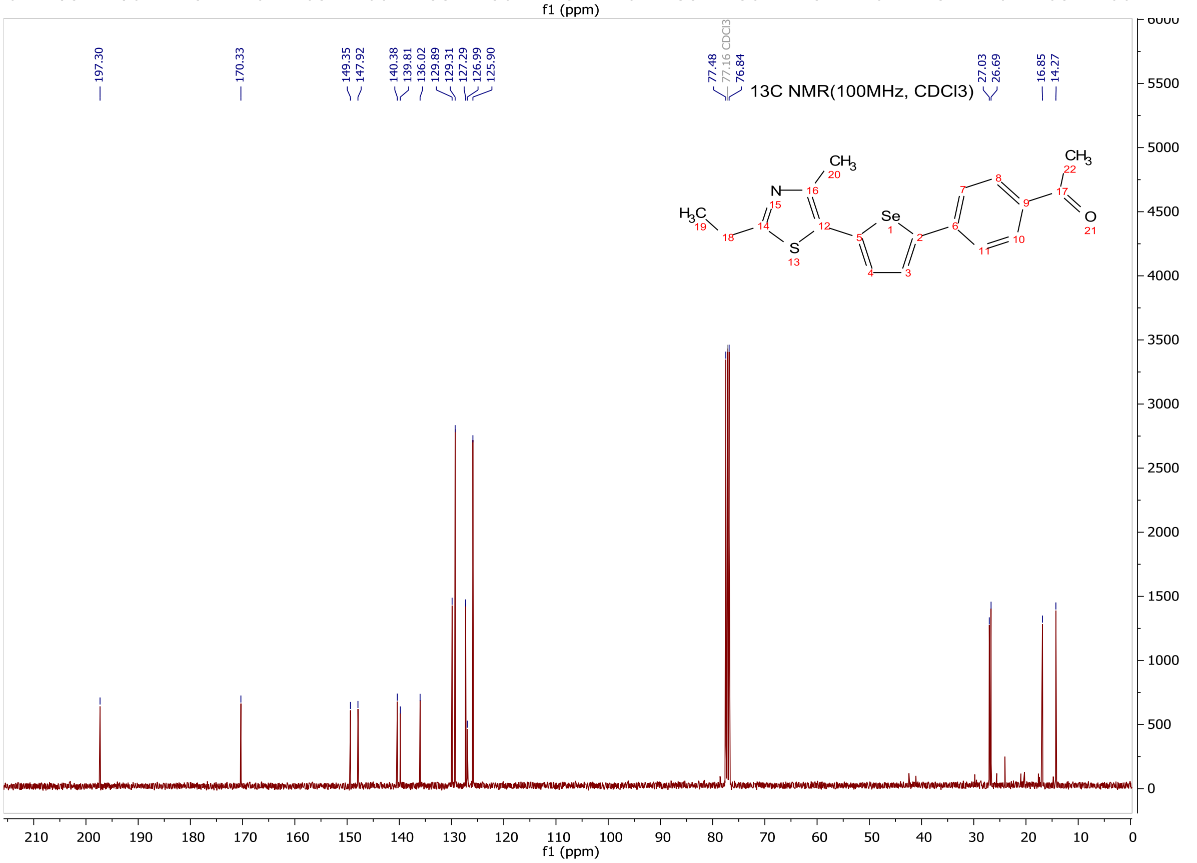

4-(5-(5-Chlorothiophen-2-yl)selenophen-2-yl)benzonitrile (21)

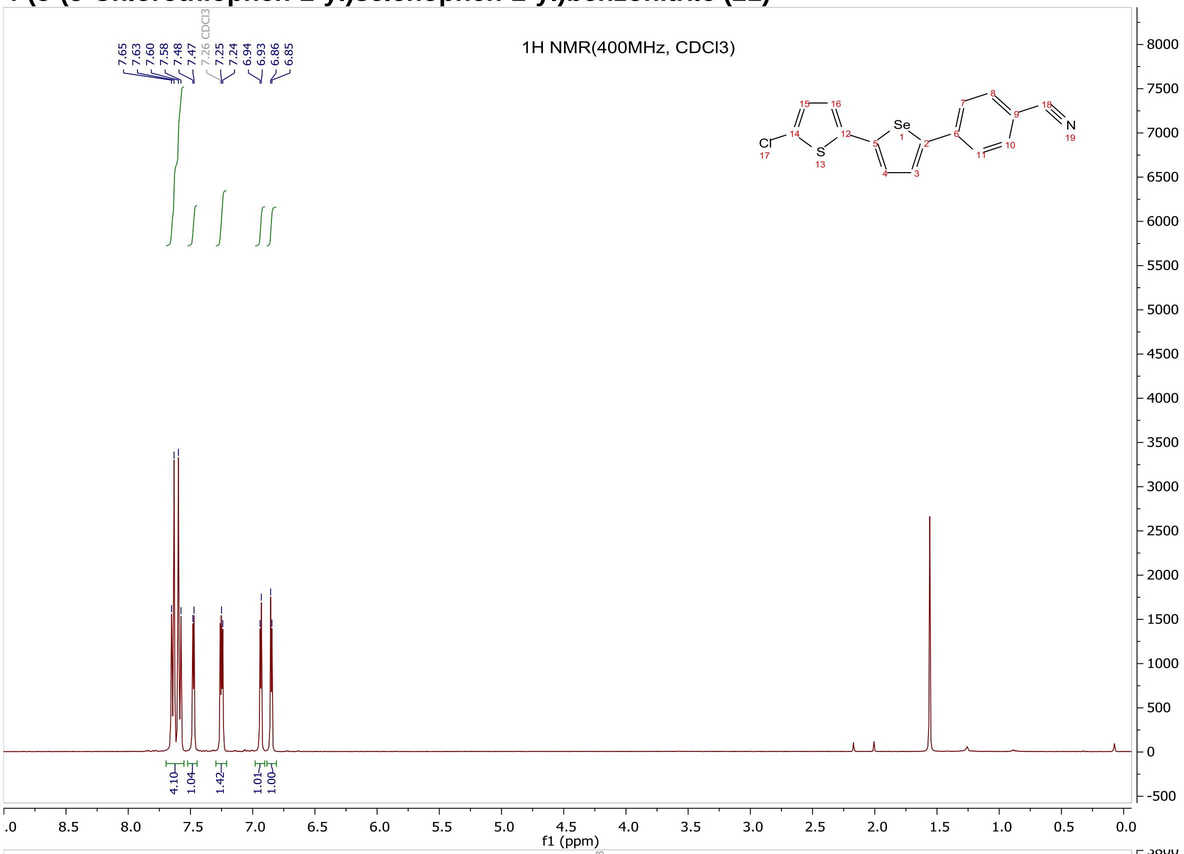

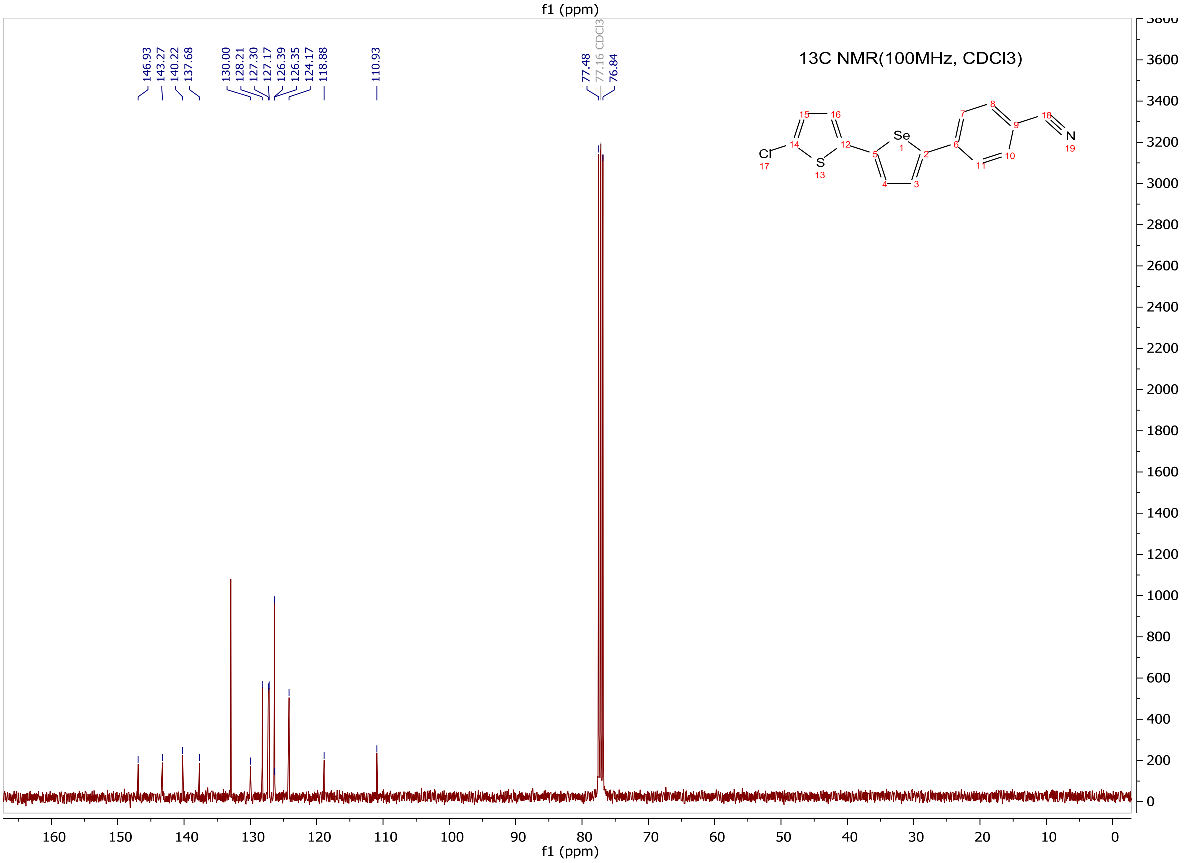

2-Chloro-5-(5-(4-chlorophenyl)selenophen-2-yl)thiophene (22)

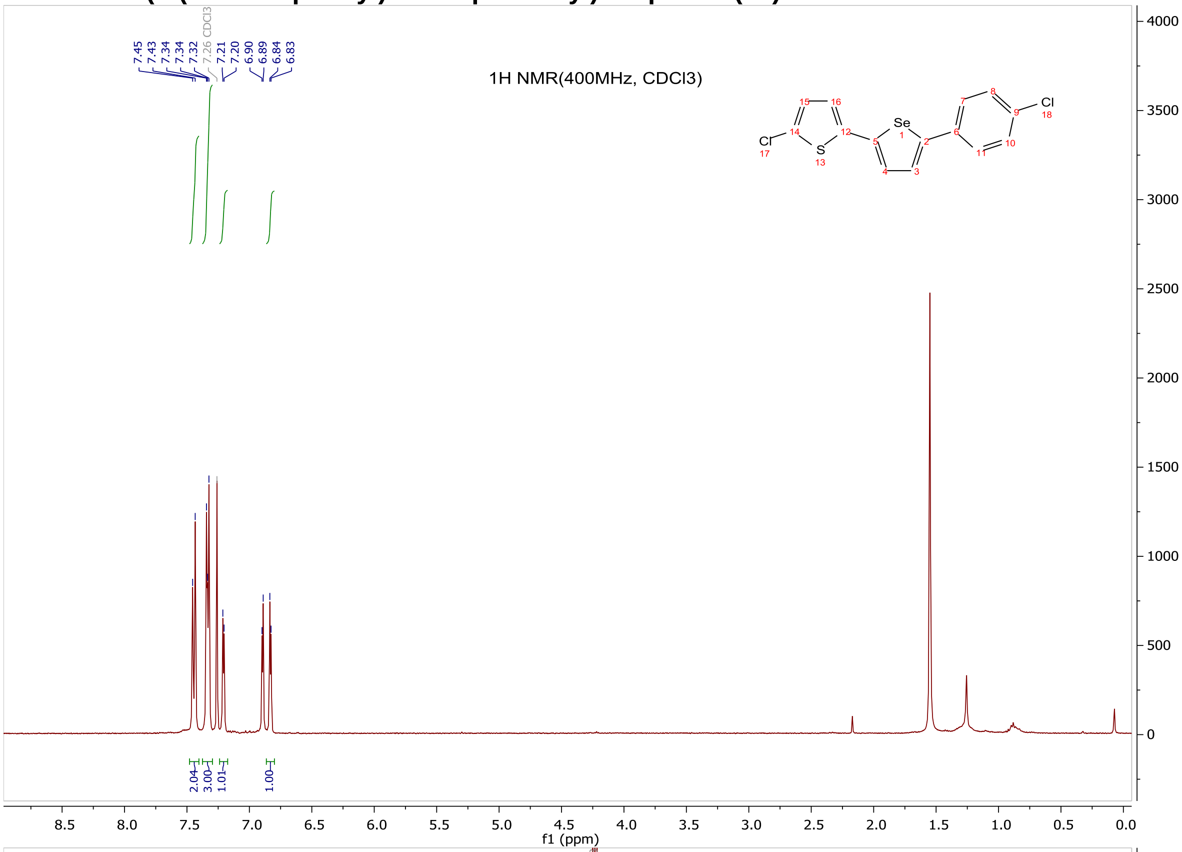

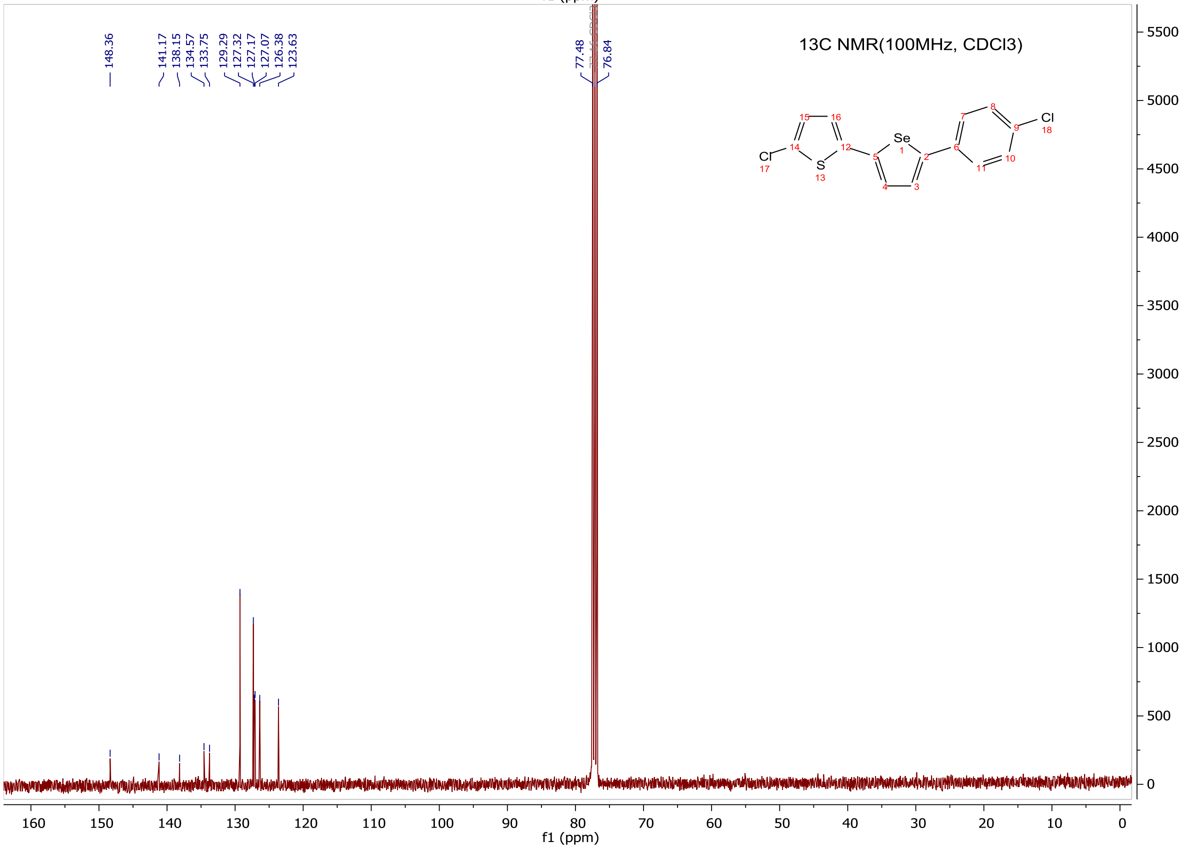

1-(4-(5-(5-Chlorothiophen-2-yl)selenophen-2-yl)phenyl)ethan-1-one (23)

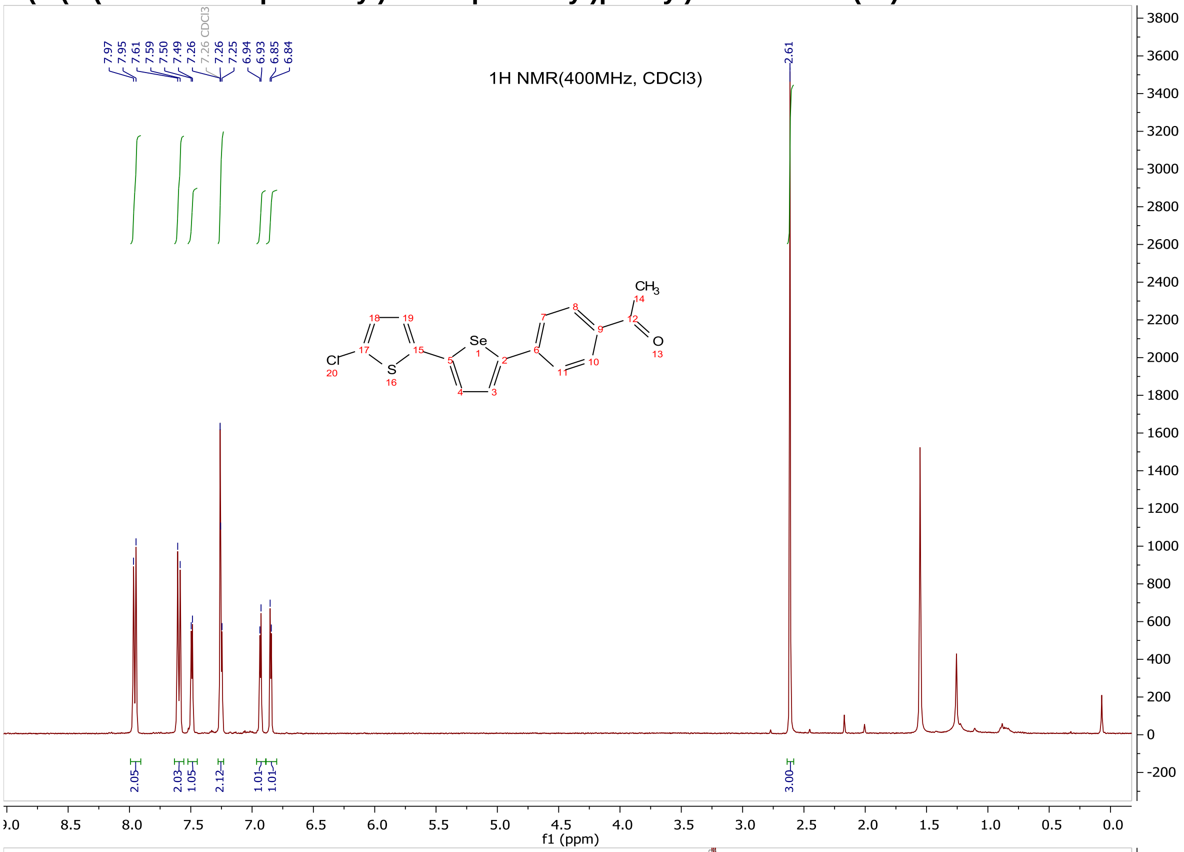

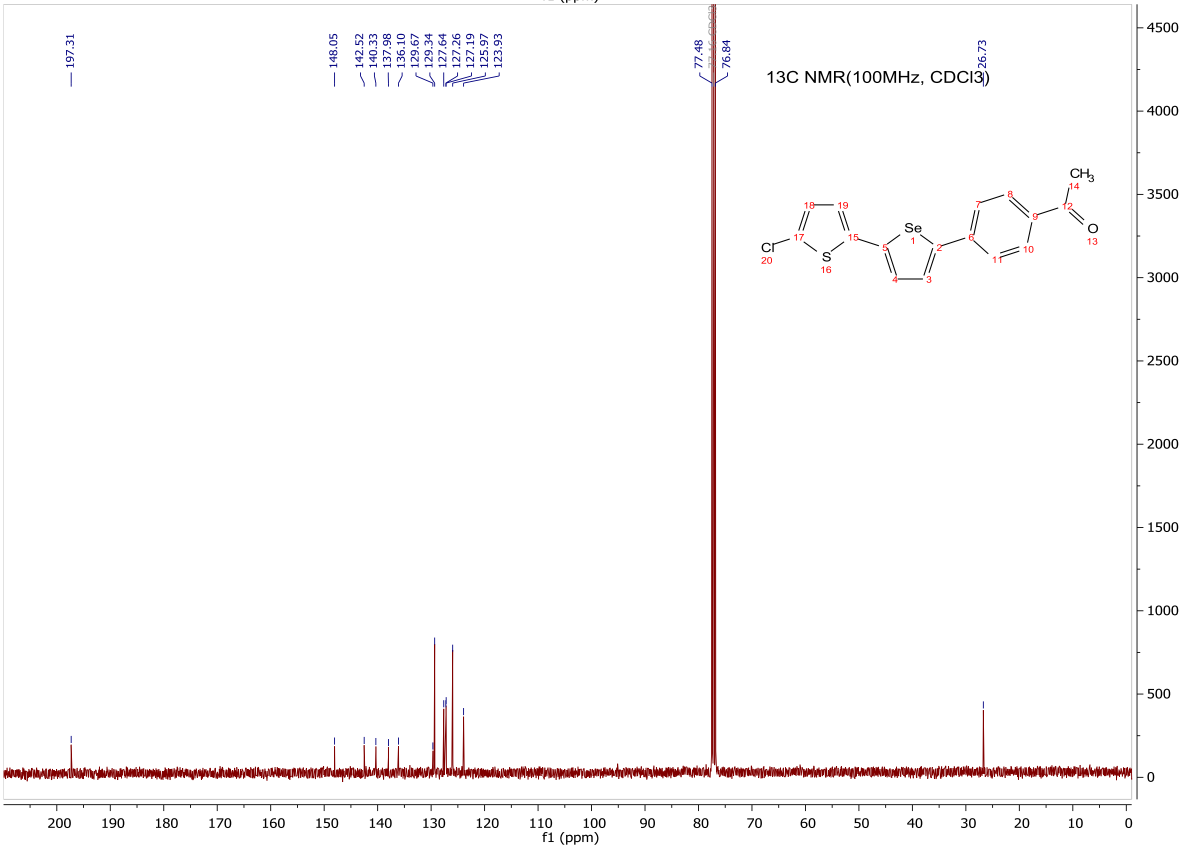

1-(4-(5-(5-Pentylthiophen-2-yl)selenophen-2-yl)phenyl)ethan-1-one (24)

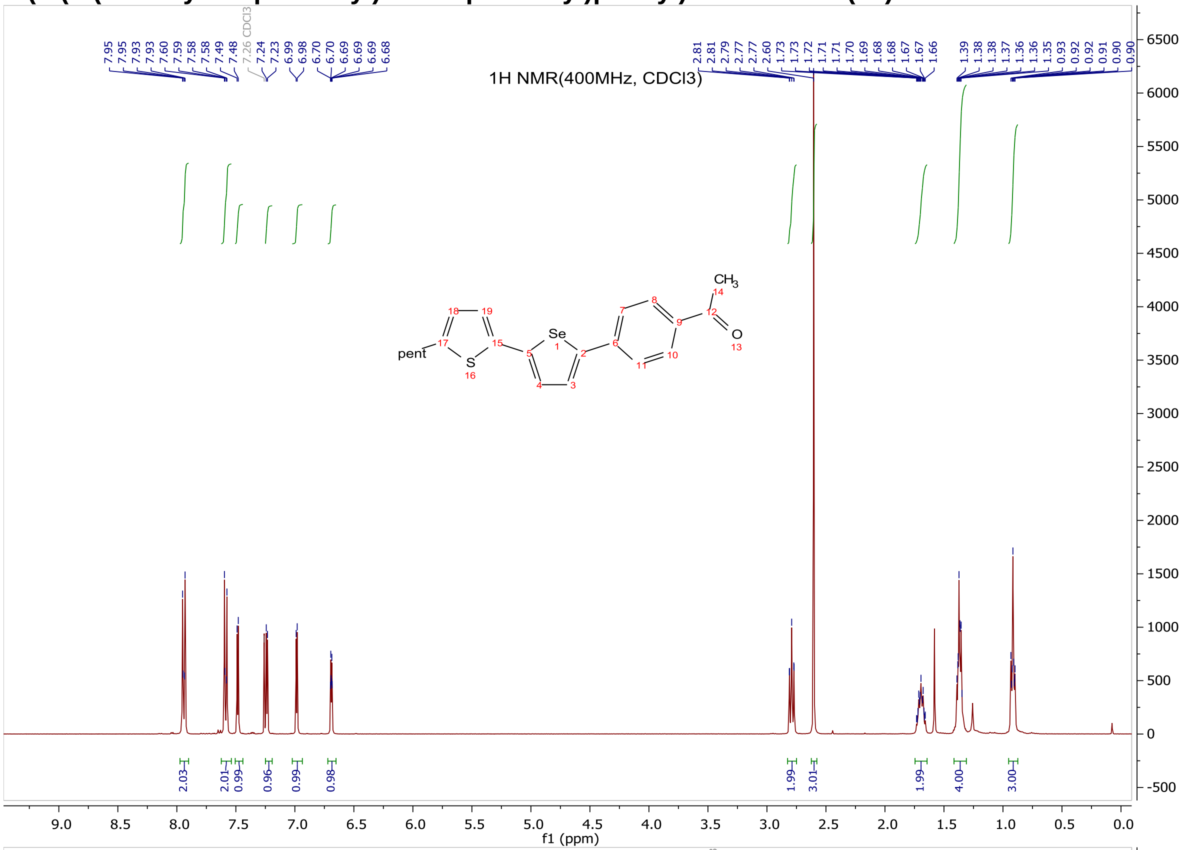

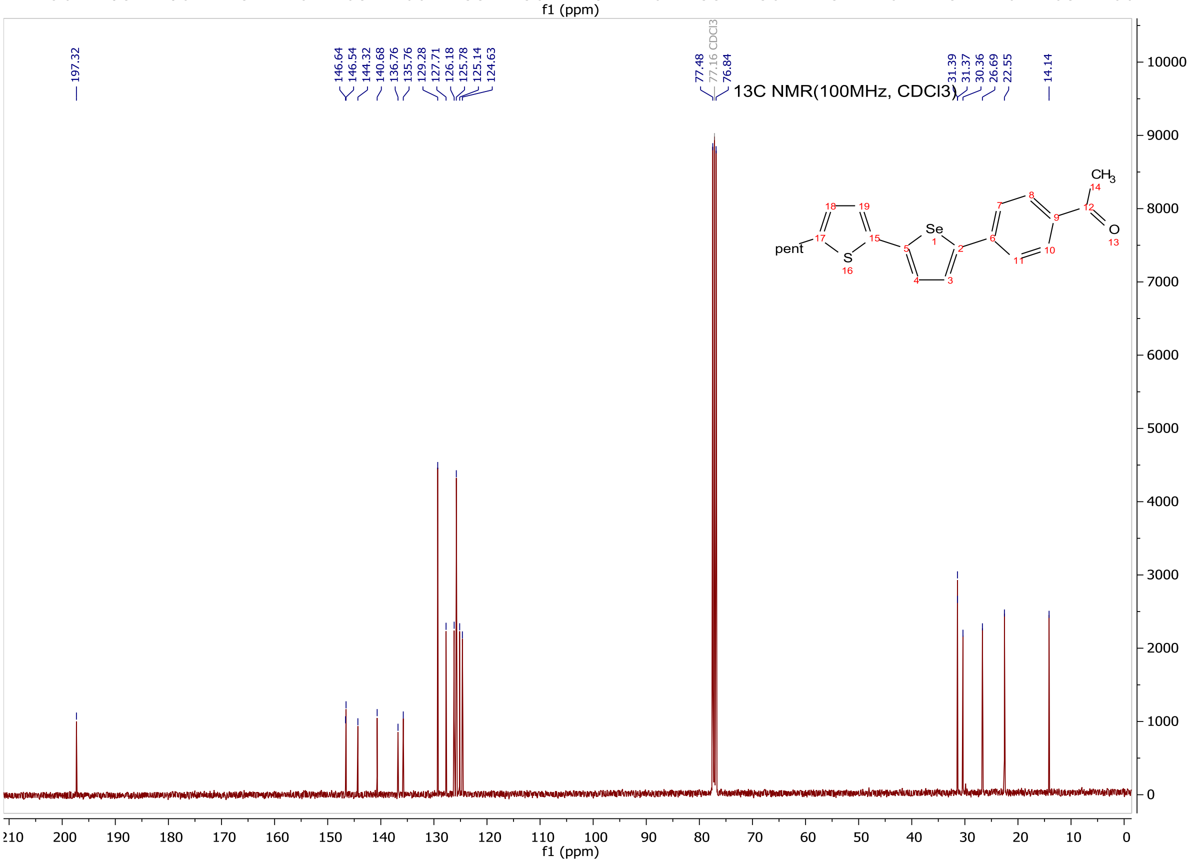

1-(4-(5-(5-Acetyl-3-chlorothiophen-2-yl)selenophen-2-yl)phenyl)ethan-1-one (25)

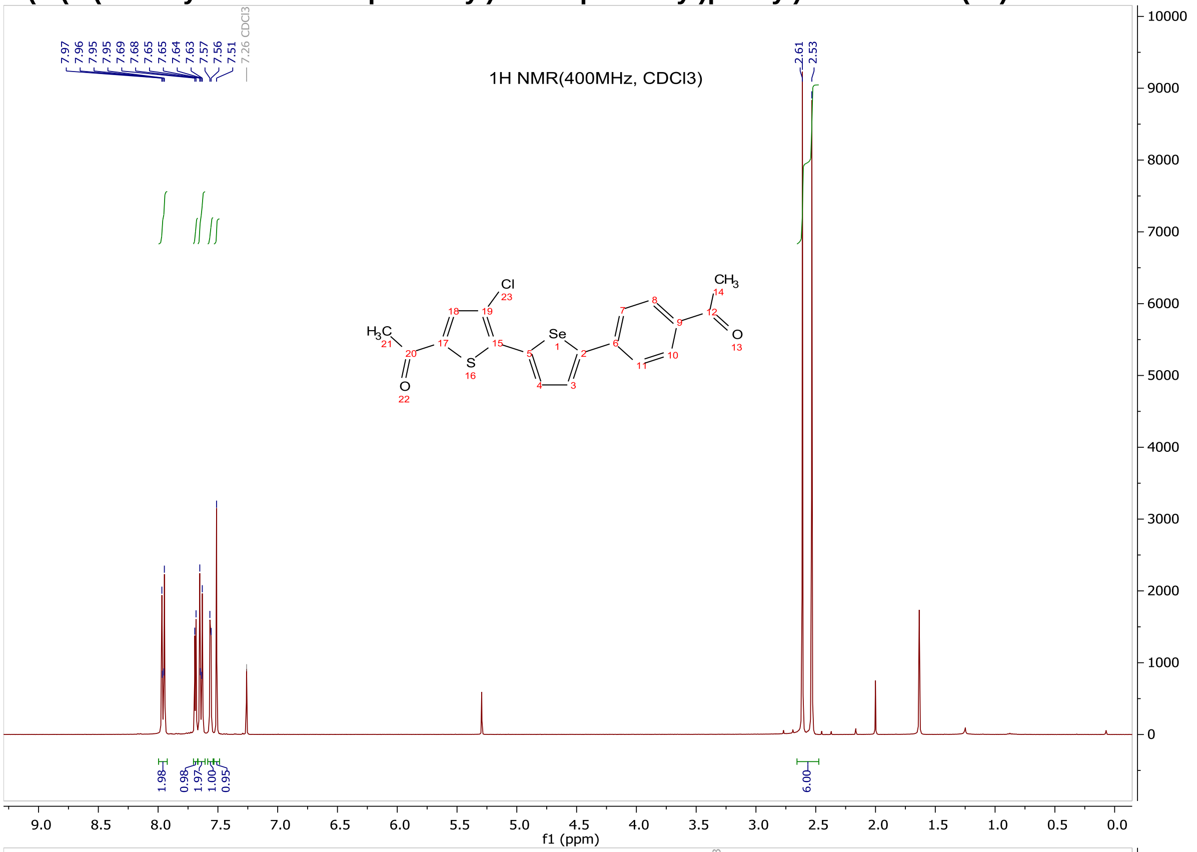

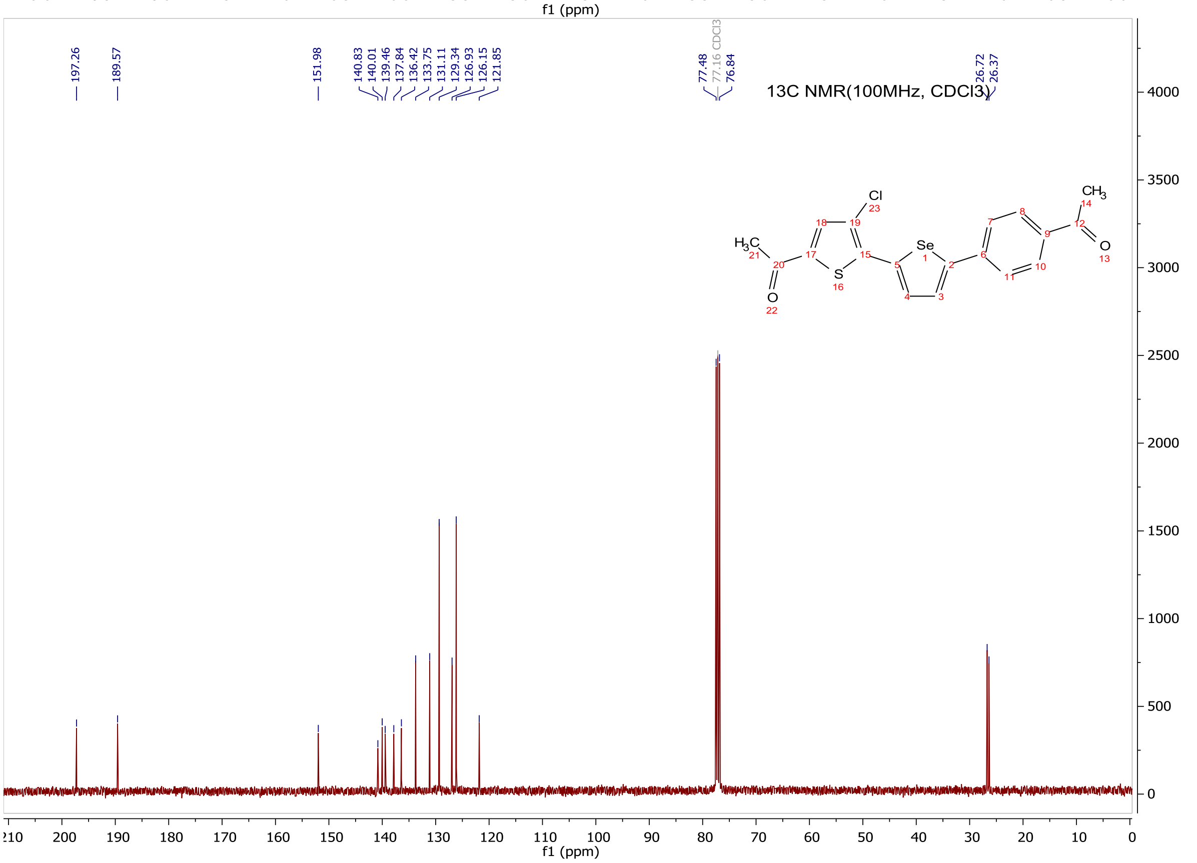

1-(4-(5-(3-Chlorothiophen-2-yl)selenophen-2-yl)phenyl)ethan-1-one (26)

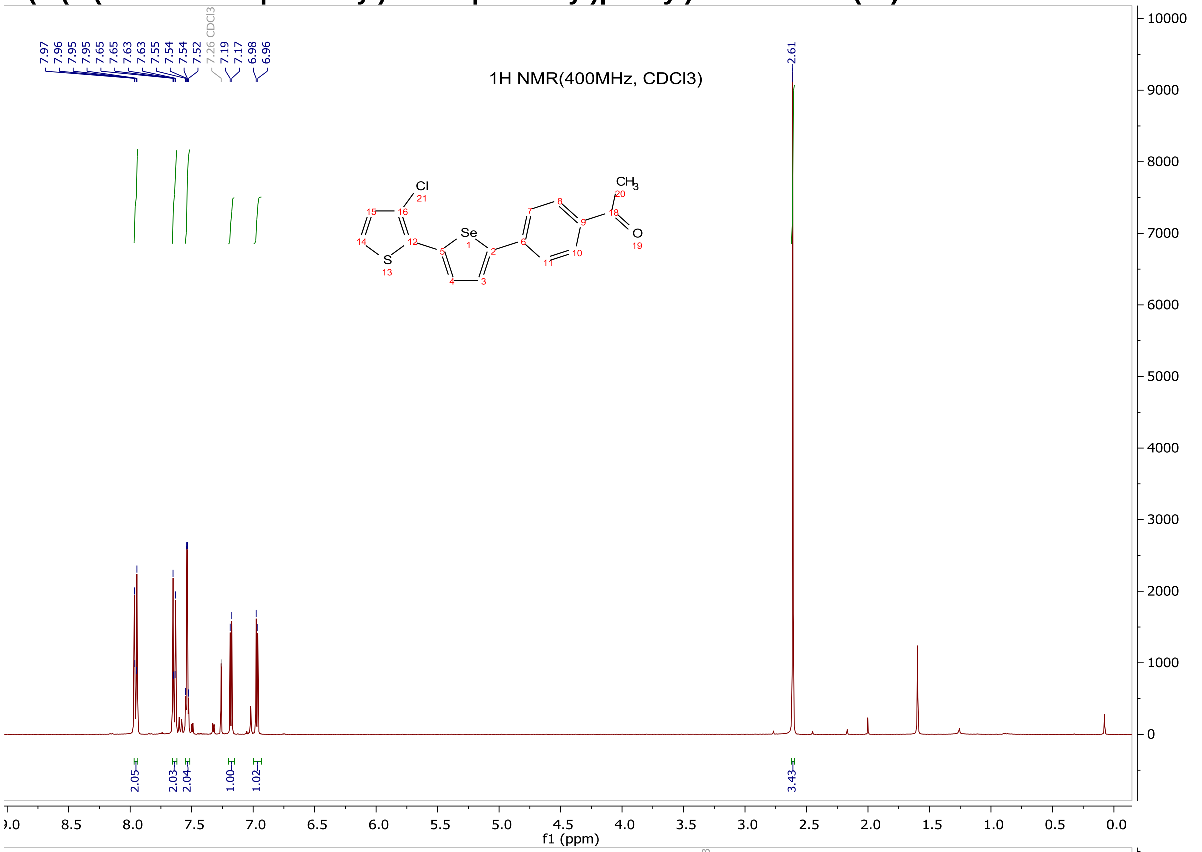

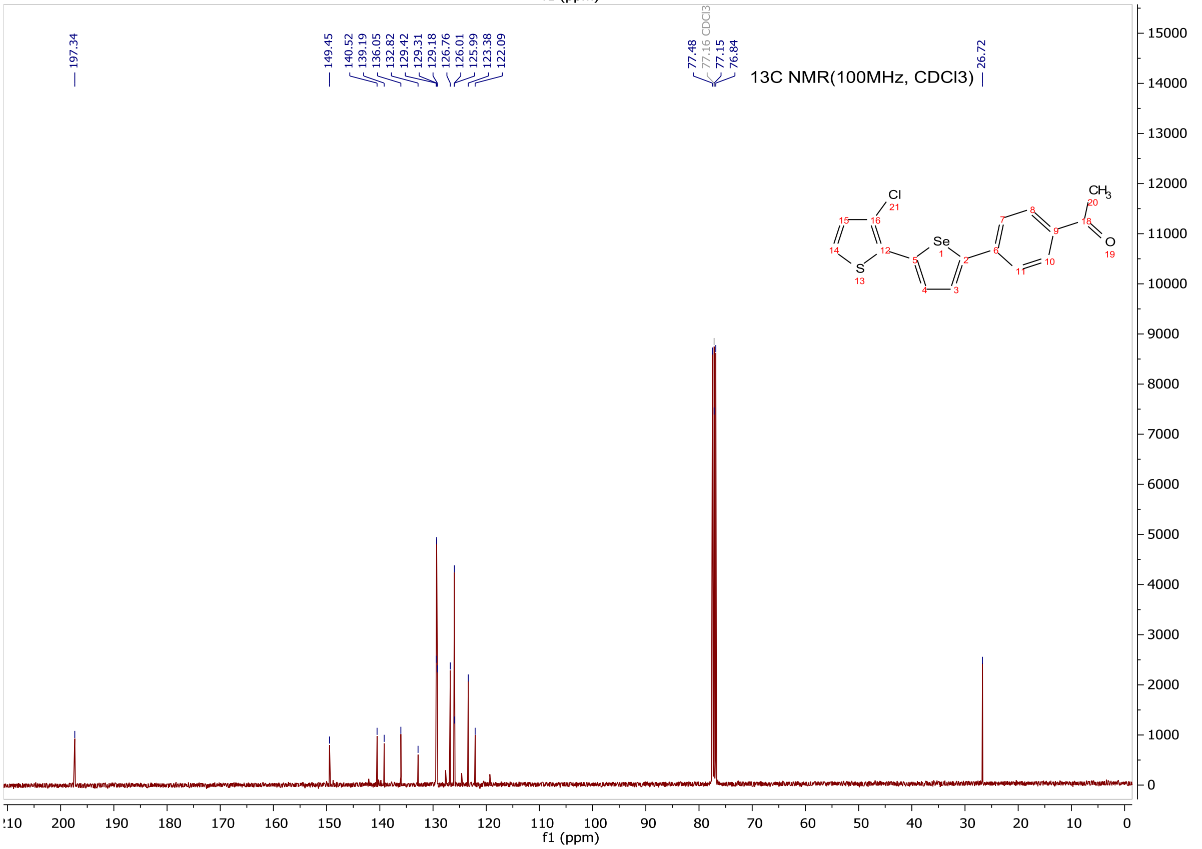

Supplement: File 1 — Additional experimental and analytical data and copies of NMR spectra. [file Beilstein_J_Org_Chem-13-2862-s001.pdf]
